# Assessment of a Precision Medicine Analysis of a Behavioral Counseling Strategy to Improve Adherence to Diabetes Self-management Among Youth: A Post Hoc Analysis of the FLEX Trial

**DOI:** 10.1001/jamanetworkopen.2019.5137

**Published:** 2019-05-31

**Authors:** Anna R. Kahkoska, Michael T. Lawson, Jamie Crandell, Kimberly A. Driscoll, Jessica C. Kichler, Michael Seid, David M. Maahs, Michael R. Kosorok, Elizabeth J. Mayer-Davis

**Affiliations:** 1 Department of Nutrition, University of North Carolina at Chapel Hill, Chapel Hill; 2 Department of Biostatistics, University of North Carolina at Chapel Hill, Chapel Hill; 3 School of Nursing, University of North Carolina at Chapel Hill, Chapel Hill; 4 Barbara Davis Center for Childhood Diabetes, University of Colorado Denver, Aurora; 5 Cincinnati Children’s Hospital Medical Center, University of Cincinnati Medical School, Cincinnati, Ohio; 6 Department of Pediatrics, School of Medicine, Stanford University, Stanford, California; 7 Stanford Diabetes Research Center, Stanford University, Stanford, California; 8 Department of Statistics and Operations Research, University of North Carolina at Chapel Hill, Chapel Hill; 9 Department of Medicine, University of North Carolina at Chapel Hill, Chapel Hill

## Abstract

**Importance:**

The Flexible Lifestyles Empowering Change (FLEX) trial, an 18-month randomized clinical trial testing an adaptive behavioral intervention in adolescents with type 1 diabetes, showed no overall treatment effect for its primary outcome, change in hemoglobin A_1c_ (HbA_1c_) percentage of total hemoglobin, but demonstrated benefit for quality of life (QoL) as a prespecified secondary outcome.

**Objective:**

To apply a novel statistical method for post hoc analysis that derives an individualized treatment rule (ITR) to identify FLEX participants who may benefit from intervention based on changes in HbA_1c_ percentage (primary outcome), QoL, and body mass index *z* score (BMIz) (secondary outcomes) during 18 months.

**Design, Setting, and Participants:**

This multisite clinical trial enrolled 258 adolescents aged 13 to 16 years with type 1 diabetes for 1 or more years, who had literacy in English, HbA_1c_ percentage of total hemoglobin from 8.0% to 13.0%, a participating caregiver, and no other serious medical conditions. From January 5, 2014, to April 4, 2016, 258 adolescents were recruited. The post hoc analysis excluded adolescents missing outcome measures at 18 months (2 participants [0.8%]) or continuous glucose monitoring data at baseline (40 participants [15.5%]). Data were analyzed from April to December 2018.

**Interventions:**

The FLEX intervention included a behavioral counseling strategy that integrated motivational interviewing and problem-solving skills training to increase adherence to diabetes self-management. The control condition entailed usual diabetes care.

**Main Outcomes and Measures:**

Subgroups of FLEX participants were derived from an ITR estimating which participants would benefit from intervention, which would benefit from control conditions, and which would be indifferent. Multiple imputation by chained equations and reinforcement learning trees were used to estimate the ITR. Subgroups based on ITR pertaining to changes during 18 months in 3 univariate outcomes (ie, HbA_1c_ percentage, QoL, and BMIz) and a composite outcome were compared by baseline demographic, clinical, and psychosocial characteristics.

**Results:**

Data from 216 adolescents in the FLEX trial were reanalyzed (166 [76.9%] non-Hispanic white; 108 teenaged girls [50.0%]; mean [SD] age, 14.9 [1.1] years; mean [SD] diabetes duration, 6.3 [3.7] years). For the univariate outcomes, a large proportion of FLEX participants had equivalent predicted outcomes under intervention vs usual care settings, regardless of randomization, and were assigned to the muted group (HbA_1c_: 105 participants [48.6%]; QoL: 63 participants [29.2%]; BMIz: 136 participants [63.0%]). Regarding the BMIz univariate outcome, mean baseline BMIz of participants assigned to the muted group was lower than that of those assigned to the intervention and control groups (muted vs intervention: mean difference, 0.48; 95% CI, 0.21 to 0.75; *P* = .002; muted vs control: mean difference, 0.86; 95% CI, 0.61 to 1.11; *P* < .001); this group also had a higher proportion of individuals with underweight or normal weight using weight status cutoffs (95 [69.9%] in muted group vs 24 [54.6%] in intervention group and 11 [30.6%] in control group; χ^2^_4_ = 24.67; *P* < .001). The approach identified subgroups estimated to benefit based on HbA_1c_ percentage (54 participants [25.0%]), QoL (89 participants [41.2%]), and BMIz (44 participants [20.4%]). Regarding the HbA_1c_ percentage outcome, participants expected to benefit from the intervention did not have significantly higher baseline HbA_1c_ percentages than those expected to benefit from usual care (9.4% vs 9.2%; difference, 0.2%; 95% CI, −0.16% to 0.56%; *P* = .44). However, participants in the muted group had higher mean HbA_1c_ percentages at baseline than those assigned to the intervention or control groups (muted vs intervention: 9.9% vs 9.4%; difference, 0.5%; 95% CI, 0.13% to 0.89%; *P* = .02; muted vs control; 9.9% vs 9.2%; difference, 0.7%; 95% CI, 0.34% to 1.08%; *P* = .001). No significant differences were found between subgroups estimated to benefit in terms of the composite outcome from the FLEX intervention (91 participants [42.1%]) vs usual care (125 participants [57.9%]).

**Conclusions and Relevance:**

The precision medicine approach represents a conceptually and analytically novel approach to post hoc subgroup identification. More work is needed to understand markers of positive response to the FLEX intervention.

**Trial Registration:**

ClinicalTrial.gov identifier: NCT01286350

## Key Points

**Question:**

Are there subgroups of participants in the Flexible Lifestyles Empowering Change (FLEX) trial for whom the intervention is estimated to be optimal, for whom usual care is estimated to be optimal, and for whom control conditions and intervention are estimated to be equivalent?

**Findings:**

In this post hoc analysis of the FLEX randomized clinical trial of 258 adolescents with type 1 diabetes, an individualized treatment rule showed that a large proportion of participants had equivalent predicted outcomes under intervention vs usual care settings, regardless of randomization.

**Meaning:**

The precision medicine approach is a conceptually and analytically novel method for post hoc subgroup analysis of randomized clinical trials, capturing data-driven response subgroups without relying on predefined categories.

## Teaser

This post hoc precision medicine analysis of the Flexible Lifestyles Empowering Change (FLEX) trial estimates an individualized treatment rule to uncover subgroups of responders for key outcomes in diabetes self-management among youth.

## Introduction

The Flexible Lifestyles Empowering Change (FLEX) trial, a National Institutes of Health–funded 18-month randomized clinical trial, tested the efficacy of an adaptive behavioral intervention to promote self-management and improve measures of blood glucose control in 258 adolescents aged 13 to 16 years with type 1 diabetes. The goal of the FLEX intervention was to increase adherence to type 1 diabetes self-management through a behavioral counseling strategy that integrated motivational interviewing and problem-solving skills training.^1^ Despite high retention and fidelity, the FLEX study did not show efficacy with respect to the primary outcome of hemoglobin A_1c_ (HbA_1c_) percentage 18 months after randomization.^1^ However, the intervention was associated with improvements in several secondary psychosocial outcomes, including motivation, problem-solving skills, diabetes self-management, and health-related and general quality of life (QoL).^1^

Within any randomized clinical trial, the average treatment effect across all participants may mask important heterogeneous treatment effects that exist among different study participants or subgroups of participants.^2^ Therefore, in the setting of significant heterogeneity in response, aggregate reporting of average treatment can also obfuscate whether some strategies helped some participants while harming others.^3,4^ For this reason, statistical analyses that estimate aggregate effects over time for all participants are limited because they do not account for the fact that treatment responders and nonresponders can exhibit vastly divergent patterns of response.^5^

Analysts who wish to account for treatment effect heterogeneity have several available tools. Analysis of differential response observed in a completed trial may take the form of an effect modification analysis, in which the observed effect of an intervention is examined across levels of a third, prespecified effect modifier variable. For example, effect modification analyses of the FLEX trial showed that the intervention effect was stronger among participants with high vs low baseline HbA_1c_ percentages at 12 months (effect, 0.69%; 95% CI, 0.07%-1.31%; *P* for interaction = 0.03) and for female vs male participants at the 18 months (difference, 0.62; 95% CI, 0.03-1.21; *P* for interaction = 0.04).^6^

In settings where more complex heterogeneity in participant profiles reliably predicts differential response to the efficacy of treatment, the precision medicine approach offers particular promise and addresses certain limitations of traditional effect modification.^7^ The precision medicine approach seeks to develop an individualized treatment rule (ITR), a mathematical function that gives recommendations for whether a participant should receive intervention or not. In the FLEX trial, treatment conditions (intervention vs usual care) were assigned at baseline, so an ITR could base its recommendations solely on participant characteristics available at baseline. As the goal of the FLEX trial was to optimize a participant’s improvement over the full 18-month course of the study, the ITR was estimated based on those 18-month improvements in outcome, also called *clinical rewards*. Once an ITR is estimated, it can be used to target intervention to those participants whom it estimates will benefit most. An ITR can be summarized based on its value, which is the average expected clinical reward that results from applying the ITR. That is, the value of an ITR represents the average clinical reward the participant cohort would have received if that ITR were followed rather than the observed randomization scheme. Individualized treatment rules that deliver the best achievable clinical reward are termed *optimal*. Estimating and applying optimal ITRs may lead to increases in efficiency of prevention and treatment while simultaneously reducing costs of care.^7,8^ As such, gaining a deeper understanding of the subgroups defined by an optimal ITR—ie, understanding which participants receive improved outcomes under an intervention and which do not—is critical to inform future tailoring of interventions.

In this study, we describe a method for the post hoc analysis of randomized clinical trials that leverages the full data set to identify subgroups defined by an estimated optimal ITR. We demonstrate how this method may be applied to data from the FLEX trial to quantify and describe the subgroups where key clinical outcomes were improved with the intervention, the subgroups where outcomes were improved with usual care, and the subgroups where outcomes were the same between intervention and usual care. To be consistent with the primary and secondary outcomes of the parent study, we characterized the effect of the FLEX intervention in terms of 18-month changes in HbA_1c_ percentage (primary outcome) as well as perceived QoL and body mass index *z* score (BMIz), a significant cardiovascular disease risk factor. We focused on baseline predictors, including sociodemographic characteristics, clinical variables, or psychosocial and behavioral measures, as these can serve as markers to clinicians in the future to guide optimal treatment recommendations regarding the FLEX intervention using the data available at that time.

## Methods

### Study Sample

We analyzed data from the baseline visit of the FLEX trial. This randomized clinical trial tested an adaptive, 18-month intervention that included interventions to improve behavioral skills and problem solving for adolescents with type 1 diabetes, with respect to HbA_1c_ percentage of total hemoglobin (primary outcome), glycemic variability, cardiovascular disease risk factors, health-related QOL, and cost-effectiveness.^1,9^ Eligible participants were adolescents aged 13 to 16 years with type 1 diabetes for 1 or more years with literacy in English, HbA_1c_ percentage of total hemoglobin of 8.0% to 13.0% (to convert to proportion of total hemoglobin, multiply by 0.01), at least 1 primary caregiver available to participate, and no other serious medical conditions or pregnancy.^9^ Those randomized to the control arm received usual diabetes care. All participants were mailed a health summary, a brief report of key clinical measures, including body mass index, laboratory values (HbA_1c_ and lipid levels), blood pressure, and select behavioral self-reported results, such as physical activity/inactivity, dietary intake, self-monitoring of blood glucose, and missed insulin. The full trial protocol is available in Supplement 1. All participants also received a copy of Pink Panther *Understanding Diabetes: A Handbook for People Who Are Living With Diabetes*,^10^ a type 1 diabetes educational book for patients and families. The FLEX study was reviewed by institutional review boards at all participating institutions. All participants and their caregivers provided informed written assent and consent, respectively. Detailed considerations of the FLEX design and baseline participant characteristics have been described elsewhere.^9^ As a randomized clinical trial, all reporting was standardized to the Consolidated Standards of Reporting Trials (CONSORT) reporting guideline. The present study was not prespecified in the FLEX trial protocol.

### Inclusion Criteria

**Figure 1.**
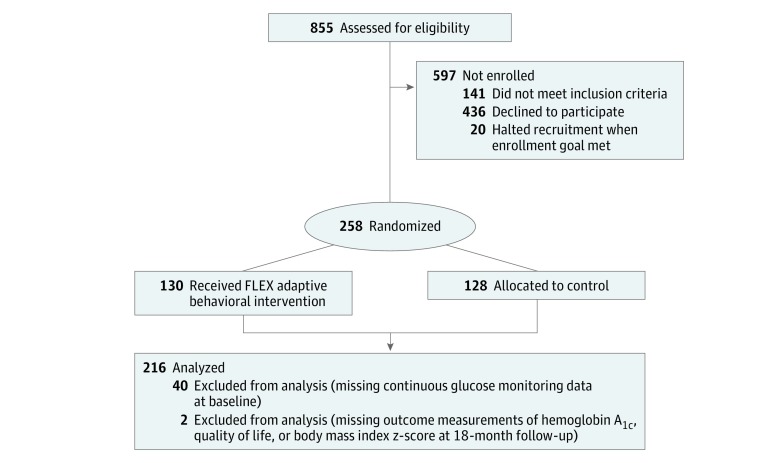
CONSORT Diagram for the Flexible Lifestyles Empowering Change (FLEX) Intervention Randomized Clinical Trial and Post Hoc Analysis

The FLEX trial enrolled 258 adolescents with type 1 diabetes who were instructed to wear masked continuous glucose monitoring systems for 7 days at baseline. Participants were excluded from the present analysis if they did not have complete continuous glucose monitoring data at baseline (40 participants [15.5%]) or were missing the outcomes of HbA_1c_ percentage, QoL, or BMIz measures at baseline or the 18-month measurement visit (2 participants [0.8%]) (Figure 1).

### Measures

All data collection was standardized per FLEX study protocol, and FLEX assessment staff were trained and certified to perform all study procedures. The full set of study measurements was obtained at baseline, 6 months, and 18 months postrandomization; a limited set of measurements was obtained at 3 months and 12 months postrandomization.^9^ Standardized measurements, laboratory data, clinical measures, and questionnaires from the FLEX study are described in detail in eAppendix 1 in Supplement 2. Standardized questionnaires were used to collect self-reported race/ethnicity, which was classified as non-Hispanic white, non-Hispanic black, Hispanic, and other, including Asian/Pacific Islander, Native American, or unknown.

### Outcome Measures

To assess the intervention’s efficacy for our outcomes of interest individually, we considered 3 univariate outcomes: change in HbA_1c _percentage, change in self-reported QoL score (measured by the Pediatric Quality of Life Inventory^11^; range, 0-100), and constrained change in BMIz. For each univariate outcome, we considered changes from baseline to the 18-month visit. For HbA_1c_ percentage and QoL, this change was directly equal to the difference between baseline and 18-month outcomes. Change in BMIz was constrained to reward patients who completed the study with a healthy BMIz or who improved their BMIz during the study. For explanations of the univariate outcomes, see Table 1; for full mathematical definitions, see eAppendix 1 in Supplement 2.

**Table 1.** Definition of Univariate Outcomes and Composite Outcome

Outcome	Measure	Type	Definition	Interpretation
Glycemic control	HbA_1c_ %	Univariate	HbA_1c_ % at 18 mo minus HbA_1c_ % at baseline	Negative values are better
QoL	PedsQL score	Univariate	QoL at 18 mo minus QoL at baseline	Positive values are better
Weight status	BMIz	Univariate	If BMIz at 18 mo ≤1.04, set equal to 1^a^	Higher values are better
			If BMIz at 18 mo >1.04 but lower than baseline, set equal to 1^a^	
			If BMIz at 18 mo >1.04 and higher than baseline, value scaled from 0 and 1^a^	
Composite	NA	Composite	See eAppendix 1 in Supplement 2	Higher values are better

Abbreviations: BMIz, body mass index *z* score; HbA_1c_, hemoglobin A_1c_; NA, not applicable; PedsQL, Pediatric Quality of Life Inventory; QoL, quality of life.

^a^ BMIz of 1.04 corresponds to the 85th percentile for age and sex.

To assess the intervention’s effect on all outcomes of interest simultaneously, we considered a composite outcome of change in HbA_1c _percentage, QoL score, and BMIz between baseline and 18-month study visits. The composite outcome approximates constrained optimization based on a hierarchy of the univariate outcomes, with HbA_1c_ percentage prioritized the highest and BMIz prioritized the lowest. In essence, participants with elevated HbA_1c_ levels would receive a low composite outcome regardless of their QoL score and BMIz; participants with acceptable HbA_1c_ levels but unacceptably low QoL scores would receive slightly higher composite outcomes regardless of their BMIz; and participants would receive the highest composite outcomes if they had acceptable HbA_1c_ and QoL levels, with the magnitude determined by their BMIz. Sensitivity analyses explored different hierarchies of the composite outcome; the final definition was selected based on consensus from the FLEX study team to be most consistent with the goals of the FLEX intervention (data not shown). For a full discussion of the composite outcome’s definition and properties, see eAppendix 1, eAppendix 2, eAppendix 4, eTable 1-6, and eFigures 1-3 in Supplement 2.

### Analysis

#### Imputation

Missing data in covariates not derived from continuous glucose monitoring were imputed via multiple imputation by chained equations, a flexible imputation method that can account for data of mixed types.^12,13^ We generated 11 imputed data sets with multiple imputation by chained equations, with which we used a modified version of multiple imputation. We chose 11 as the smallest odd number larger than 10, which precluded the possibility of ties in a majority vote. As a sensitivity analysis, we performed all analyses on the subset of patients with complete cases in all covariates and outcomes (197 participants [91.2%]; eAppendix 3, eTable 7, and eTable 9 in Supplement 2).

#### ITR Estimation

**Figure 2.**
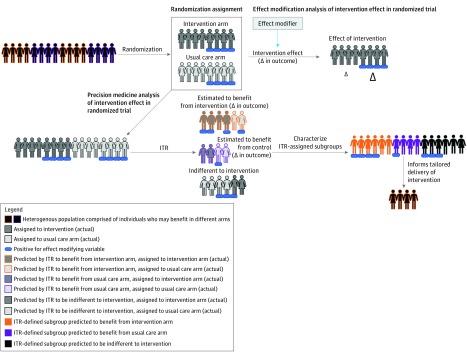
Overview of Post Hoc Analyses of Differential Response in Randomized Trial Data Effect modification analysis of intervention effect is when the observed effect of an intervention is examined across levels of a third, prespecified effect-modifier variable. Precision medicine analysis of the intervention effect is when an individualized treatment rule (ITR) is applied to the entire data set to estimate subgroups expected to benefit from the intervention, expected to benefit from usual care, and expected to be indifferent to treatment group. Δ indicates change.

We estimated the optimal ITR in our sample with reinforcement learning trees (RLT). Figure 2 visualizes a conceptual overview of our use of ITR estimation to determine subgroups. An extension of the random forest model, RLT uses reinforcement learning to better discriminate between signal and noise variables among the covariates.^14,15^ According to convention and to ensure numerical stability, continuous covariates were transformed to have a mean of 0 and an SD of 1 before analysis, and outcomes were transformed to range from 0 to 1 (univariate outcomes) or 0 to 3 (composite outcome), with higher values indicating better clinical outcomes. A noteworthy aspect of RLT is its numerical tendency to mute covariates, ie, to set their effect to 0, in subsets of the covariate space. The details of how and why muting occurs are best left for the technical discussion in the article by Zhu et al^14^; for this analysis, we simply note that the predicted outcome under the different values of a binary variable, such as intervention status, can be different for some patients but equal for others, representing either a lack of sufficient information to distinguish between levels of the binary variable or a differential effect of this binary variable that is small and difficult to distinguish from 0. Using RLT allowed us to pose a nonparametric model between the baseline covariates, *X*, and the observed clinical rewards, *R*, within each imputed data set. Using this model, we estimated the expected clinical reward for a given participant under both intervention and usual care. The ITR specific to the data set assigned a patient to 1 of 3 groups: (1) the intervention group, (2) the control group, or (3) the muted group. If the expected clinical reward was higher in the intervention group, then intervention was expected to benefit that participant and they were assigned to the intervention group. If the expected clinical reward was higher in the usual care group, then usual care was expected to benefit that participant and they were assigned to the control group. Finally, if the expected clinical reward did not differ between the intervention and usual care groups, then intervention status was not expected to have any large effect for that participant and they were assigned to the muted group. For each participant, we obtained 11 assignments, 1 assignment per imputed data set. The estimated optimal ITR assigned each patient to the group designated by a plurality vote of these 11 assignments. Once the groups were defined by the ITR, we examined their baseline demographic, clinical, and psychological and social characteristics.

#### ITR Evaluation

Once estimated, each ITR was evaluated based on its value, *V̂*, the expected clinical reward resulting from applying the ITR to the sample rather than the observed randomization scheme. We used the following estimate of *V̂*:
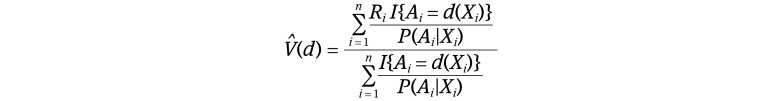
where *I{E}* is the indicator function that takes the value 1 when event *E* is true and 0 otherwise, *i* indexes patients, *A* is the vector of observed intervention assignments, and *d* is the estimated ITR. Point estimates of ITR values were computed using this formula, and CIs for ITR values and differences in ITR values were computed via bootstrapping, as described in eAppendix 3 in Supplement 2.

### Statistical Analysis

Descriptive statistics were used to describe characteristics of individuals in each post hoc, ITR-defined subgroup. Descriptive data are presented as mean and SD, number and percentage, or median and interquartile range for variables that are not normally distributed, such as measures of hypoglycemia. Comparisons were performed using χ^2^ or analysis of variance (Fisher exact and Wilcoxon-Mann-Whitney tests, where appropriate). Pairwise comparisons were performed using χ^2^ or *t* tests (Fischer exact or Wilcoxon signed-rank tests, where appropriate). As these analyses were exploratory, the Benjamini-Hochberg method was used to adjust for multiple comparisons; this method controls for the false discovery rate rather than the familywise error rate.^16^ A 2-tailed *P* value less than .05 was considered statistically significant. Imputation and ITR estimations were carried out in R version 3.4.1 (The R Foundation), using the packages missForest, RLT, and DTRlearn. Descriptive analyses were conducted using SAS version 9.4 (SAS Institute).

## Results

### Study Sample

The final study sample included 216 adolescents with type 1 diabetes in the FLEX trial (166 non-Hispanic white participants [76.9%]; 108 adolescent girls [50.0%]; mean [SD] age, 14.9 [1.1] years; mean [SD] type 1 diabetes duration, 6.3 [3.7] years at baseline). At baseline, the mean (SD) HbA_1c_ percentage of total hemoglobin was 9.6% (1.2%) (to convert to proportion of total hemoglobin, multiply by 0.01), mean (SD) QoL score was 81.2 (12.4), and mean (SD) BMIz was 0.73 (0.91).

### ITR Estimation and Evaluation

Table 2 depicts 2 measures of interest for evaluating the RLT ITR. The first measure is the estimated value of *V̂* across the composite outcome and each univariate outcome. The second is the comparison between the value of the estimated optimal ITR and the fixed treatment effects for intervention and usual care, which are computed as *V̂* with *d(X)* assigning intervention or usual care to all participants, respectively. Note that each column of this table has a different natural scale owing to the particular distribution of outcomes in question. All estimates of fixed treatment comparisons lie above 0, and all but 1 of the 95% CIs are greater than 0, indicating that the estimated optimal ITR achieved higher expected clinical rewards than blanket assignment of treatment or usual care.

**Table 2.** Value and Fixed Treatment Comparisons for Reinforcement Learning Tree Imputed ITR

Estimated Values^a^	Outcome Measure Used to Estimate Optimal ITR^b^			
	HbA_1c_	QoL	BMIz	Composite
V̂_opt_	0.6738	0.6739	0.9737	2.6985
V̂_opt_ − V̂_trt _(95% CI)^c^	0.0109 (−0.0028 to 0.0372)	0.0152 (0.0004 to 0.0766)	0.0233 (0.0018 to 0.0376	1.2085 (1.1058 to 1.3716)
V̂_opt_ − V̂_ctrl _(95% CI)^c^	0.0171 (0.0033 to 0.0433)	0.0225 (0.0077 to 0.0839)	0.0234 (0.0019 to 0.0377)	1.0067 (0.9040 to 1.1698)

Abbreviations: BMIz, body mass index *z* score; HbA_1c_, hemoglobin A_1c_; ITR, individualized treatment rule; QoL, quality of life.

^a^ V̂_opt _denotes the estimated value obtained by assigning patients according to the estimated optimal ITR. Higher values denote higher performance. V̂_trt _denotes the estimated value obtained by assigning all participants to intervention. Higher values denote higher performance. V̂_ctrl _denotes the estimated value obtained by assigning all participants to usual care. Higher values denote higher performance.

^b^ Range for HbA_1c_, QoL, and BMIz, 0 to 1; range for composite, 0 to 3. Each column has a different natural scale owing to the particular distribution of outcomes in question.

^c^ 95% CIs based on the bootstrap, described in eAppendix 3 in Supplement 2.

### Characteristics of ITR-Assigned Subgroups

Table 3 depicts the baseline characteristics found to be significantly different among participants in the subgroups assigned to intervention and control groups for the composite outcome and among participants in the subgroups assigned to intervention, control, and muted groups for the univariate outcomes. For full descriptive tables, see eTable 8 in Supplement 2. Except for the composite outcome, a large number of participants were assigned to the muted group, whose predicted outcomes under intervention and control conditions appeared equivalent.

**Table 3.** Baseline Characteristics of Flexible Lifestyles Empowering Change Participants by Reinforcement Learning Tree Individual Treatment Rule–Assigned Subgroups^a^

Baseline Characteristic^b^	All	Composite Outcome			HbA_1c_				QoL				BMIz			
		Intervention Group (n = 91)	Control Group (n = 125)	*P* Value^c^	Intervention Group (n = 54)	Muted Group (n = 105)	Control Group (n = 57)	*P* Value^c^	Intervention Group (n = 89)	Muted Group (n = 63)	Control Group (n = 64)	*P* Value^c^	Intervention Group (n = 44)	Muted Group (n = 136)	Control Group (n = 36)	*P* Value^c^
HbA_1c_ %, mean (SD)	9.6 (1.2)	9.7 (1.3)	9.6 (1.2)	.72	9.4 (1.0)	9.9 (1.4)^d^	9.2 (0.9)	.01^e^	9.8 (1.2)	9.4 (1.3)	9.6 (1.2)	.52	9.6 (1.2)	9.6 (1.3)	9.9 (1.2)	.68
HbA_1c_ >9.0%, No. (%)	140 (64.8)	63 (69.2)	77 (61.6)	.68	31 (57.4)	81 (77.1)^d^	28 (49.1)	.01^e^	67 (75.3)	34 (54.0)	39 (60.9)	.52	26 (59.1)	85 (62.5)	29 (80.6)	.43
BMIz score, mean (SD)	0.73 (0.91)	0.79 (0.82)	0.68 (0.97)	.72	0.65 (0.89)	0.80 (0.89)	0.67 (0.98)	.67	0.64 (0.91)	0.73 (0.94)	0.86 (0.87)	.91	0.97 (0.73)	0.49 (0.94)^d^	1.3 (0.6)^d^	<.001^e^
Weight category, No. (%)																
Underweight or normal weight	130 (60.2)	52 (57.1)	78 (62.4)	.72	38 (70.4)	59 (56.2)	33 (27.9)	.57	57 (64.0)	37 (58.7)	36 (56.3)	.93	24 (54.6)	95 (69.9)^d^	11 (30.6)^d^	<.001^e^
Overweight	54 (25.0)	27 (29.7)	27 (21.6)		8 (14.8)	29 (27.6)	17 (29.8)		23 (25.8)	14 (22.2)	17 (26.6)		12 (27.3)	30 (22.1)^d^	12 (33.3)^d^	
Obese	32 (14.8)	12 (13.2)	20 (16.0)		8 (14.8)	17 (16.2)	7 (12.3)		9 (10.1)	12 (19.1)	11 (17.2)		8 (18.2)	11 (8.1)^d^	13 (26.1)^d^	
CGM measures^f^																
Hypoglycemic episodes with blood glucose <70 mg/dL, median (IQR), No.	2 (1-6)	2 (1-6)	3 (1-6)	.97	1 (0-5)	4 (1-6)	2 (0-6)	.07	2 (0-5)	3 (1.0-5.5)	3.5 (1-7)	.91	4 (1-5)	3 (1-7)	2 (0.5-4.5)	.60
Hypoglycemic episodes with blood glucose <54 mg/dL, median (IQR), No.	1 (0-2)	1 (0-2)	1 (0-2)	.97	0 (0-2)	1 (1-3)^d^	0 (0-2)	<.001^e^	1 (0-2)	1 (0-2)	1 (0-3)	.93	0 (0-1.5)	1 (0-3)	0 (0-2)	.39

Abbreviations: BMIz, body mass index *z* score; CGM, continuous glucose monitoring; HbA_1c_, hemoglobin A_1c_; IQR, interquartile range; QOL, quality of life.

SI conversion factor: to convert to proportion of total hemoglobin, multiply by 0.01.

^a^ The imputed data set–specific individualized treatment rule assigned a patient to 1 of 3 groups. If the expected clinical reward was higher in the intervention group, then intervention was expected to benefit that participant and that participant was assigned to intervention. If the expected clinical reward was higher in the usual care group, then usual care was expected to benefit that participant and that participant was assigned to usual care. If the expected clinical reward was identically equal in the usual care and intervention groups, then intervention status was expected to have no association with the outcome for that participant and they were assigned to the muted group. No participants were muted for the composite outcome.

^b^ Only characteristics with statistically significant differences across individualized treatment rule–assigned subgroups are shown.

^c^ *P* values are from χ^2^ or Fisher exact test for categorical variables and *t* tests or Kruskal-Wallis test for continuous variables. The Benjamini-Hochberg procedure was used to control for the false discovery rate in multiple comparisons.

^d^ Significant test of differences (*P* < .05).

^e^ Significant pairwise comparison, compared with intervention group (*P* < .05).

^f^ Measures derived over 7-day period of masked continuous glucose monitor wear time. Episodes defined as lasting 15 minutes or more. Data were right skewed.

Regarding the composite outcome, 91 participants (42.1%) were assigned to the intervention group, while the remaining 125 participants (57.9%) were assigned to the control group. There were no significant differences in other participant characteristics according to the ITR-assigned subgroups (Table 3).

Regarding the HbA_1c_ percentage univariate outcome, 105 participants (48.6%) were assigned to the muted group, 54 participants (25.0%) were assigned to the intervention group, and 57 participants (26.4%) were assigned to the control group. Participants assigned to the intervention group did not have significantly higher baseline HbA_1c_ percentages than those assigned to the control group (9.4% vs 9.2%; difference, 0.2%; 95% CI, −0.16% to 0.56%; *P* = .44), but participants in the muted group had higher mean HbA_1c_ percentages at baseline than those assigned to the intervention or control groups (muted vs intervention: 9.9% vs 9.4%; difference, 0.5%; 95% CI, 0.13% to 0.89%; *P* = .02; muted vs control; 9.9% vs 9.2%; difference, 0.7%; 95% CI, 0.34% to 1.08%; *P* = .001). Participants in the muted group also had a higher incidence of clinically serious hypoglycemia at baseline (χ^2^_2_ = 29.87;* P* < .001), with no significant differences between participants in the intervention group vs the control group (Table 3).

Regarding the QoL univariate outcome, 63 participants (29.2%) were assigned to the muted group, 89 participants (41.2%) were assigned to the intervention group, and 64 participants (29.6%) were assigned to the control group. There were no significant differences in participant characteristics according to the ITR-assigned subgroups (Table 3).

Regarding the BMIz univariate outcome, 136 participants (63.0%) were assigned to the muted group, 44 participants (20.4%) were assigned to the intervention group, and 36 participants (16.7%) were assigned to the control group. Mean BMIz at baseline of participants assigned to the muted group was lower than that of those assigned to the intervention and control groups (muted vs intervention: mean difference, 0.48; 95% CI, 0.21-0.75; *P* = .002; muted vs control: mean difference, 0.86; 95% CI, 0.61-1.11; *P* < .001); this group also had a higher proportion of individuals with underweight or normal weight using weight status cut-offs (95 [69.9%] in muted group vs 24 [54.6%] in intervention group and 11 [30.6%] in control group; χ^2^_4_ = 24.67; *P* < .001). Mean baseline BMIz was also significantly higher in the intervention group compared with the control group (mean difference, 0.38; 95% CI, 0.09-0.67; *P* < .001) (Table 3).

## Discussion

In this article, we presented a method to identify subgroups of participants in a randomized clinical trial for whom the intervention would have been beneficial, for whom usual care would have been beneficial, and for whom neither option would have made a difference regarding key clinical outcomes. We then applied this method to reanalyze data from the FLEX trial, which initially showed no effects of the intervention on the primary and secondary study outcomes, to demonstrate that there are distinct subgroups with different optimal treatment assignments. The discussion focuses first on the findings from the post hoc analysis of the FLEX trial and then turns to a more general discussion of the method itself.

The application of a method to find distinct subgroups (intervention, control, and muted) within a single randomized clinical trial sample is appropriate given previous reports of heterogeneity in response to behavioral interventions,^17^ including heterogeneity of response to the same intervention in different samples of adolescents with type 1 diabetes.^18,19^ The relative proportions of these subgroups, especially regarding the large muted group for HbA_1c_ percentage as a univariate outcome, highlight the challenges of glycemic control in this age range.^1^ By contrast, a larger group was estimated to benefit from the FLEX intervention regarding QoL, which agrees with the main trial’s findings that the FLEX intervention had a positive aggregate effect on multiple measures of psychosocial well-being.^1^ The large proportion of participants assigned to the muted group for the univariate BMIz outcome likely reflects both the relatively short time period of follow-up (18 months) as well as the challenges of using BMIz to evaluate change in adiposity.^20^

The results also reveal interesting dynamics in the interplay among the 3 univariate outcomes. Clinical markers that link interventions to subgroups of participants that are likely to benefit take a central role in precision application of interventions;^8^ as such, it would be ideal to have a large variety of markers corresponding to differential estimated response patterns.^8,21^ Although we considered a range of participant characteristics, only a limited subset of characteristics emerged to distinguish the ITR-assigned subgroups in the FLEX study. Furthermore, the markers were not consistent across the 3 univariate outcomes, in which no markers emerged that were consistent across different outcomes.

A finding of interest was the lack of baseline features to distinguish groups based on estimated response patterns. We believe the antagonistic associations among the univariate outcomes may contribute to the paucity of reliable factors for the subgroups governed by the composite outcome, even among covariates that helped predict subgroups for subgroups governed by univariate outcomes. This finding speaks to the importance of using and optimizing a composite outcome according to the study of interest and research question. Using a composite outcome may help avoid logical inconsistencies that occur from considering univariate outcomes in isolation, as it has here. In this analysis, the composite outcome was designed to reflect the primary and secondary goals of the trial, but additional analyses could explore other outcomes, such as improvement in glycemic control constrained by the incidence of hypoglycemia, which represents a major, potentially life-threatening adverse effect of insulin intensification.

The results suggest that RLT estimated an optimal ITR that performed well for the FLEX data. As Table 2 shows, the estimated optimal ITR achieved a high *V̂* for each outcome. For each outcome, the estimated optimal ITR nominally outperformed the 2 fixed treatment effects. All bootstrapped 95% CIs corresponding to these fixed treatment comparisons except 1 were greater than 0, suggesting that the estimated ITRs outperformed the fixed treatment regimes. The size of the differences in Table 2 suggests the treatment effect in this trial is generally small, although this table may slightly overstate the situation owing to the scaling of outcomes.

While we chose RLT to estimate the optimal ITR for the FLEX data because of its performance, an additional philosophical advantage of using RLT is its specification of a muted group. Conceptually, this group represents a phenomenon that is distinct from the other 2 assignments; the muted group can be thought to contain true nonresponders because they do not show a response to the intervention or control conditions (which in this case was usual care). Alternatively, this group may represent adolescents for whom the estimated response was significant but equivalent under intervention and control conditions. In this study, the large size of the muted group for each univariate outcome is likely a reflection of a marginal treatment effect, combined with noise in the data. It is interesting that the muted group showed significant differences from the other 2 groups, particularly with the HbA_1c_ percentage univariate outcome, where this subgroup comprised participants with the highest mean HbA_1c_ level and incidence of hypoglycemia and could be interpreted as the patients at the highest risk with the poorest glycemia at baseline, defined by both high and low blood glucose excursions. These characteristics suggest that adolescents for whom diabetes management is particularly challenging or outcomes are particularly poor may require more intensive interventions than the FLEX intervention. More work is needed to understand the subgroup of adolescents with type 1 diabetes who fall into this category, as they represent the population who may be the most difficult to reach via intervention work and may be at the highest risk of long-term complications of the disease.

Despite the nonparametric nature of the ITR estimation, the ITR-assigned subgroups are generally consistent with findings from effect modification analyses of the FLEX trial showing that baseline HbA_1c_ percentage and sex were markers of differential response to the intervention. However, the existence of the muted group, in addition to other differences in methodology, such as exclusion criteria not shared between the present analysis and the effect modification analysis, obscures direct comparisons. Moreover, this analysis is conceptually and analytically distinct from standard subgroup analysis methods, representing a novel approach to subgroup determination with several main differences. First, the proposed method is more directly prescriptive than traditional methods—subgroups are based on a participant’s assignment according to an ITR built to maximize the overall average clinical reward in the entire participant sample. Second, the proposed method places very few restrictions compared with many traditional methods. Common ITR estimation methods, including our choice of RLT, are free of distributional assumptions, giving the proposed approach a level of robustness to model misspecifications. There are several related advantages to this method for post hoc analysis of randomized clinical trial data. As the response subgroups are not prespecified based on a hypothesized mechanism of disease, they may represent previously uncharacterized subgroups that are nevertheless relevant to the optimal delivery of intervention. Moreover, the data-driven nature of this method may help remove researcher degrees of freedom that can hinder reproducibility.^22,23^ Third, estimating an optimal ITR pools information from the entire study sample, not just the arm randomized to intervention. Additionally, the use of RLT allows us to model the intervention effect with a remarkably small number of assumptions, and the ability of RLT to handle high dimensionality allows us to consider a broad range of participant characteristics as suitable clinical markers, including aspects of clinical care, sociodemographic characteristics, and behavioral measures at baseline that may reinforce or challenge the efficacy of a given therapy over time.^21,24^

Finally, a salient advantage of the ITR-based approach is its ability to translate directly to clinical implementation. Consider an ITR that is estimated in a diverse and representative patient population and externally validated in a similarly diverse and representative patient population. Once estimated, the ITR can be applied as follows: a medical professional gathers a patient’s demographic, clinical, and psychosocial covariates at the time of treatment decision, enters them into a computer program that stores the ITR decision rule, and then receives a group assignment of intervention, control, or muted. In the former 2 cases, the ITR gives a clear recommendation; in the latter case, the 2 are estimated to be equivalent in value based on the outcomes used to estimate the ITR, suggesting that other factors that were not represented in the ITR, such as cost, might be considered. Additionally, assignment to the muted group may signal that a patient belongs to a group for which the standard intervention options may not be particularly effective, flagging these patients as key candidates for future studies exploring novel intervention options.

### Limitations

This study had limitations. A significant limitation is the lack of data available for external validation purposes. We suggest that the present analysis offers proof of principle for the feasibility and application of the analytic method, and future studies are warranted in larger trials with greater intervention effects. In addition, the limitations of this analysis include the small sample size and high degree of noise in the data, especially compared with the small effect size of the intervention. Future work may explore the application of ITR-based subgroups to other trial data of different sample sizes with a range of intervention effect magnitudes.

## Conclusions

The results of this post hoc analysis of the FLEX trial are important groundwork to expand the available tools for matching participants and subgroups of participants to existing and newly studied interventions. There remains a lack of consensus about the best approach to promote adherence and improve glycemic control among adolescents with type 1 diabetes. The precision delivery of interventions, based on a diverse breadth of data, as modeled in this study, offers a promising approach.

## Notes

Supplement 1. **FLEX Study Protocol**

Supplement 2. **eAppendix 1.** Introduction and Measures **eAppendix 2.** Properties of Composite Outcome **eAppendix 3. **Sensitivity Analyses **eAppendix 4.** Future Methodological Work **eTable 1.** Subgroup Recovery Sensitivity and Specificity in the Simple Numerical Simulation, Synergistic Setting, for Various True Univariate Treatment Effects, by True Subgroup Status **eTable 2. **Treatment Effect MSE in the Simple Numerical Simulation, Synergistic Setting, by the Values of the True Univariate Treatment Effects **eTable 3. **Treatment Effect MSE in the Simple Numerical Simulation, Antagonistic Setting, by the Values of the True Univariate Treatment Effects **eTable 4. **Subgroup Recovery Sensitivity and Specificity in the Trial-Like Numerical Simulation, Synergistic Setting, for Various True Univariate Treatment Effects, by ITR Estimation Method and True Subgroup Status **eTable 5. **Treatment Effect MSE in the Trial-Like Numerical Simulation, Synergistic Setting, by the Values of the True Univariate Treatment Effects **eTable 6. **Treatment Effect MSE in the Trial-Like Numerical Simulation, Antagonistic Setting, by the Values of the True Univariate Treatment Effects **eTable 7. **Estimated Value and Bootstrap 95% CIs for the Value Achieved by the Estimated ITR in Each Univariate Outcome and the Composite Outcome, by Dataset and ITR Estimation Method **eTable 8. **Baseline Characteristics of FLEX Participants by RLT, ITR-Assigned Subgroups for the Composite Outcome and Univariate Outcomes (HbA_1c_ Level, Quality of Life, and BMIz) **eTable 9. **Baseline Characteristics of FLEX Participants by Outcome Weighted Learning ITR-Assigned Subgroups for the Composite Outcome and Univariate Outcomes (HbA_1c_ Level, Quality of Life, and BMIz) **eFigure 1. **Summary of Simple Numerical Experiment, Synergistic Setting **eFigure 2. **Summary of Simple Numerical Experiment, Antagonistic Setting **eFigure 3.** Comparison to Univariate Outcome, Simple Numerical Experiment, Antagonistic Setting **eReferences**
