# Supplementary material for: Assessment of a Precision Medicine Analysis of a Behavioral Counseling Strategy to Improve Adherence to Diabetes Self-management Among Youth: A Post Hoc Analysis of the FLEX Trial
Source: JAMA Netw Open. 2019 May 31;2(5):e195137. doi: 10.1001/jamanetworkopen.2019.5137 (PMC6547107; doi:10.1001/jamanetworkopen.2019.5137)
Supplement: Supplement 1. — FLEX Study Protocol [file jamanetwopen-2-e195137-s001.pdf]

STUDY PROTOCOL

Study Title: **FL3X: Randomized Clinical Trial**

Principal Investigators: **Elizabeth J Mayer-Davis, PhD**
The University of North Carolina at Chapel Hill
Chapel Hill, NC

Michael Seid, PhD
Cincinnati Children's Hospital Medical Center
Cincinnati, OH

David Maahs, MD, PhD
Stanford University*
*as of September 2016

Version: November 2017

Funding:
National Institutes of Health/NIDDK, 1UC4DK101132
Helmsley Charitable Trust support for continuous glucose monitoring data collection

Contents

1. Executive Summary 1
2. Background and Significance 5
3. Study Objectives 7
4. Site Descriptions 8
5. Selection & Recruitment of Subjects 9
6. Process of Obtaining Consent 11
7. Randomization 12
8. Standardized Measurements 13
   8a. Standardized Measurements for Primary Outcome 13
   8b. Standardized Measurements for Secondary Outcomes 13
   8c. Potential mediators 13
   8d. Intervention Acceptability 14
   8e. Cost Effectiveness 14
   8f. Other 14
9. FL3X Intervention 18
   9a. Importance and Relevance 18
   9b. Adaptive Design 18
      FL3X Basic 19
      FL3X Check-in 19
      FL3X Regular 19
      Session-by-session intervention outline 19
   9c. Intervention Components 21
      Motivational Interviewing 21
      Problem-Solving Skills Training 22
      Behavioral Family Systems Therapy 23
      Toolboxes. 24
      Fidelity 25
10. Control Condition 26
11. Extended Follow-up 27
   11a. Ancillary Study Aim 27
   11b. Consent 27
   11c. Randomization 28
   11d. Standardized Measures 28
   11e. Extended Intervention 29

12. Data management 30
13. Statistical Considerations 31
Sample Size and Power 31
Statistical Analysis 32
Missing Data 33
14. Potential Risks, Discomforts, Inconveniences, & Precautions: 34
Protections against Risk 34
15. Risk/Benefit Analysis 37
16. Data Safety & Monitoring: 38
Data Monitoring 38
Population 38
Adverse Events 39
Study Stopping Rules and Related Issues 39
16. Privacy & Confidentiality 41
17. Study Timeline 43

1. EXECUTIVE SUMMARY

Effective diabetes self-management is key to maintaining euglycemia and reducing the morbidities associated with type 1 diabetes mellitus (T1D). Despite this, adherence to the complex regimen required to achieve optimal hemoglobin A1C (HbA1C) is inadequate in adolescents with T1D. Attempts to improve diabetes adherence in adolescents through technology[1], education [2], specific counseling approaches[3] including motivational interviewing (MI) [4-6 7], and diabetes specific behavioral family systems therapy (BFST-D)[8], have had mixed results or are not immediately applicable to clinical practice. Thus, there is a *critical need* for a diabetes self-management intervention approach that integrates the key elements of proven strategies in a way that is of practical importance to clinical management and immediately applicable and that is tailored to barriers to adherence for youth living with T1D. Our specific aims are:

Aim 1: **To test the efficacy of the FL3X intervention on the primary outcome of change in HbA1c, and evaluate secondary outcomes of motivation and problem solving skills, hypoglycemia derived from continuous glucose monitoring, self-management behaviors, risk factors for diabetes complications, and health-related quality of life (HRQOL).** Our *hypothesis,* based on our preliminary data, is that the FL3X intervention will be clinically and statistically superior to usual diabetes care with respect to our primary outcome at 18-month follow up.

Aim 2: **To evaluate the cost of intervention delivery and, from the societal perspective, the cost effectiveness of the FL3X intervention.** We will measure the total cost of FL3X and, in a comparison of FL3X to usual diabetes care, we will estimate cost per unit change in HbA1c and per quality adjusted life year (QALY) using the Health Utilities Index (HUI). Our *hypothesis* is that FL3X will be cost-effective.

Aim 3: **To ensure practicality and immediate applicability of the FL3X intervention.** We will make available user-friendly versions of all intervention materials and related instructional manuals, ensure the FL3X intervention is adaptable in a variety of clinical care settings, and develop strategies to address reimbursement in the rapidly changing health care payment environment.

Figure 1.1 below shows our conceptual framework in which both motivational factors and information lead to acquisition of the skills needed for adherence to diabetes self-management recommendations. Further, a wide range of barriers to adherence exist that need to be addressed in a pragmatic manner, with the youth able to problem-solve on a real-world, day-to-day basis. The FL3X intervention is designed to address these key components, with an approach that takes into account contextual factors at play that also can impact adherence and outcomes.

Figure 3.1.1. Conceptual Framework

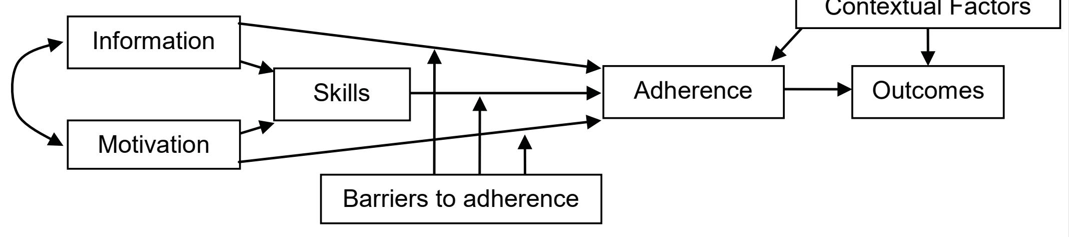

<u>The FL3X Adaptive Intervention</u> (Figure 1.2) is designed to increase adherence to T1D self-management including medical management (blood sugar testing and insulin dosing), diet, and physical activity. FL3X relies on MI, and problem-solving skills training (PSST) as the basis for

the counseling strategy, and creates a coherent integration across three key components of 1) behavior family systems therapy focused on family communications and teamwork; 2) individualized diabetes education in response to knowledge gaps relevant to behavioral goal attainment; and 3) use of currently available communications technology to support behavioral goal attainment through participant-defined reminders and motivational boosters, and/or peer support. Expected outcomes include improved HbA1c, cardiovascular disease (CVD) risk factors, and quality of life, mediated by improved diabetes self-management behaviors, and improved family communications related to diabetes.

All FL3X intervention participants will receive “FL3X-Basic”, which is the initial 3-month intervention that includes 4 sessions (40-60 min), supplemented with short additional contacts (via text, email, or web-based communication) as needed. Thereafter, applying principles of adaptive interventions, based on a decision rule using A1c values measured at defined intervals, participants are iteratively assigned to “FL3X-Check-in” or “FL3X-Regular”, both of which continue with MI and PSST for the underlying counseling strategy. In FL3X-Check-in, participants who are doing well (“responders”) will receive minimal ongoing support to reinforce successful strategies through brief monthly “touch-base” contacts. In FL3X-Regular, those who are “Regular-responders” will have a minimum of 3-4 in-person full-length sessions (40-60 min) over each 6-month interval, with additional brief contacts as needed (e.g., text, voice, or internet). These sessions will utilize modules that expand upon the three key components of FL3X-Basic (BFST, individualized diabetes education, and communication technologies in support of behavioral goal attainment) and additional brief contacts as needed (e.g., texting). **This approach allows implementation of the best of the proven strategies, specific to the needs of individual youth living with T1D, when the need exists.**

To rigorously evaluate the efficacy of the FL3X Adaptive Intervention, we will conduct a randomized clinical trial (RCT) of FL3X. Coordinated from the University of North Carolina at Chapel Hill (UNC), the FL3X RCT will include a total of 250 individuals, randomized within each of 2 sites that participated in the FL3X Basic Pilot (University of Colorado Denver, Barbara Davis Center for Childhood Diabetes; and Cincinnati Children’s Hospital Medical Center, Division of Pediatric Endocrinology). Participants randomized to intervention will receive the FL3X intervention as described. Those randomized to the control condition will receive their usual diabetes care.

All participants will also receive a Health Summary that is comprised of key clinical (e.g., HbA1c, lipid panel, BP, weight status) and behavioral (e.g., dietary intake, physical activity) information collected during the standardized measurement visit at baseline, 6 and 18 months. Control participants will be encouraged to share this information with their providers and to go to their providers with any specific questions. Control and intervention participants will be followed for 18 months.

Figure 1.2 Summary of the Flexible Lifestyles (FL3X) Adaptive Intervention

All study participants receive standardized measurements at baseline and 3, 6, 12, and 18 months after the baseline visit. Participants will be assigned to "FL3X Regular" or "FL3X Check-In" based on results of the HbA1c test obtained at the 3, 6, and 12 month measurement visits, and thus can move between these intervention intensities throughout the intervention period.

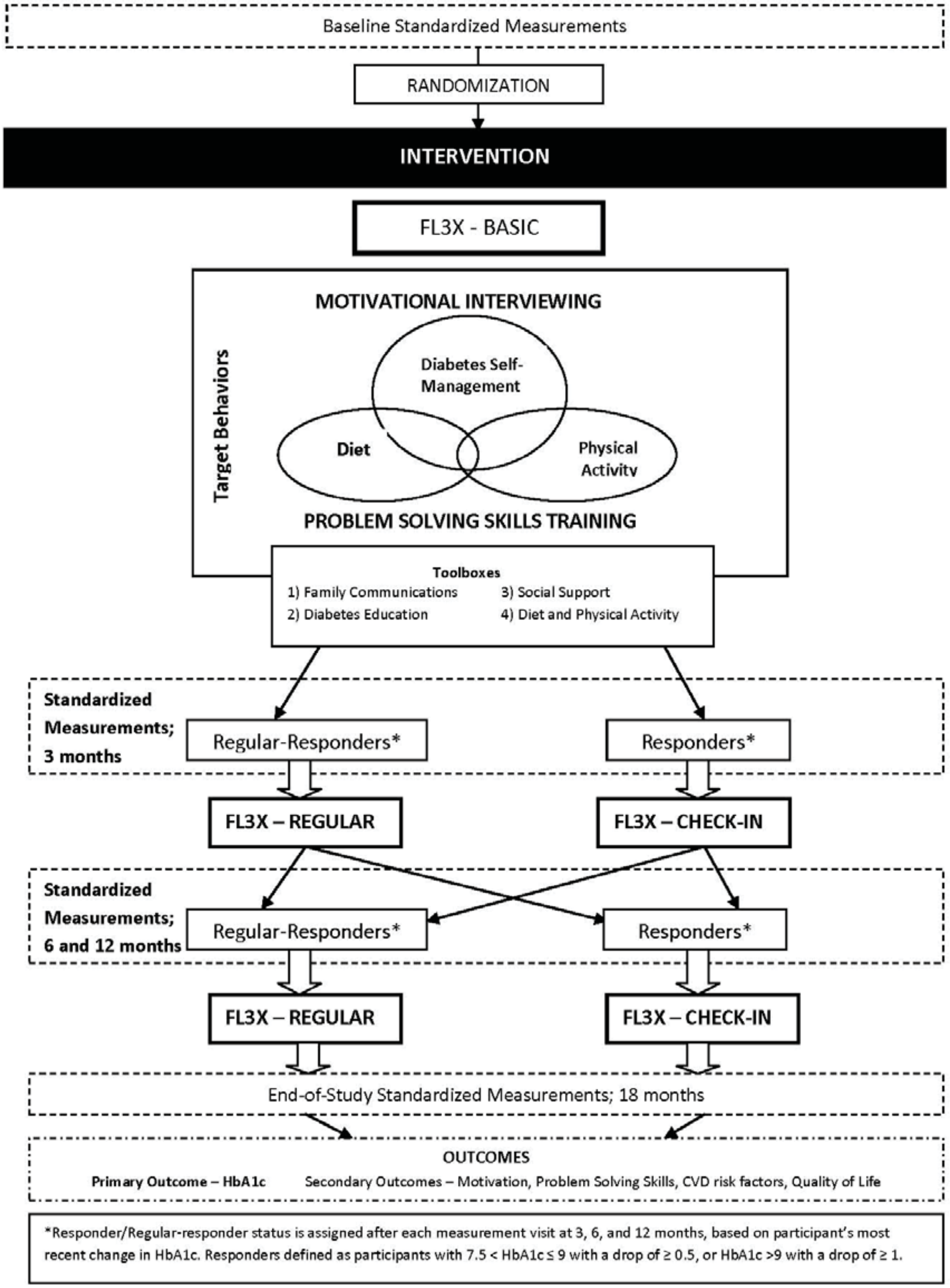

The primary outcome will be HbA1c as a measure of average glycemia over the prior 6-8 weeks. Secondary outcomes will include additional measures of glycemia, CVD risk profile, motivation, problem solving skills and health-related quality of life. Potential mediators include the diabetes self-management knowledge and behaviors and diabetes-related family communication. We will also evaluate the intervention cost and, from a societal perspective, cost effectiveness in terms of dollars per quality-adjusted life year gained.

Details of all study procedures are documented in the Manuals of Procedures (MOPs):

- Recruitment and Retention MOP
- Standardized Measurement MOP
- Intervention MOP
- Control MOP

Study organization and governance The Study Steering Committee is comprised of the Executive Committee plus the chairs of the Working Groups. Decisions are made by consensus of the Steering Committee, with formal approval needed for any modification to the protocol, and for approval of papers using FL3X data. Formal approval is established by majority vote, in which voting members of the Steering Committee include the Principal Investigator of the Coordinating Center and Principal Investigator of each clinical site, the NIDDK project scientist, and the lead statistician. Other roles of the Steering Committee include maintaining quality control and streamlining data management.

Figure 1.3 Overview of executive committee and workgroups (Voting members denoted with an asterisk (*))

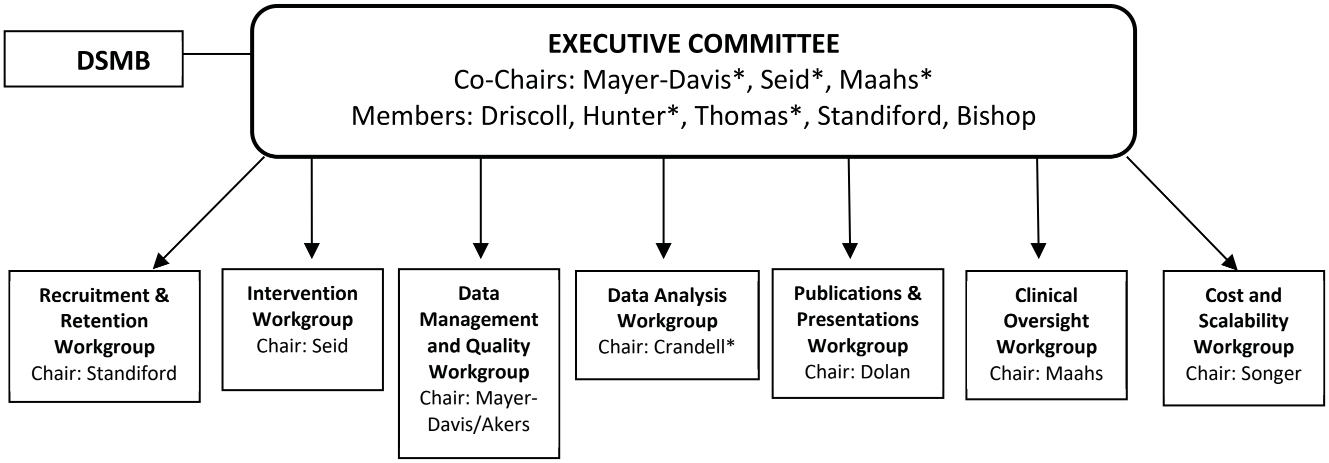

2. Background and Significance

Interventions to improve metabolic control of T1D are important to address risk for debilitating, microvascular complications[9]. Moreover, among youth with T1DM, coronary artery disease occurs earlier and is associated with higher mortality than in the general population [10 11]. Daily diabetes self-management presents a tremendous challenge. The proposed work will build upon our successful R21 pilot of FL3X Basic to advance a clinically applicable approach towards sustainable improvement in diabetes self-management behaviors and clinical outcomes, potentially improving the health and extending the lives of many tens of thousands of youth.

Table 1. Percent* of T1D Youth (n=3947) with poor glycemic control (HbA1c ≥9.5%)

**Race / Ethnicity**	
NHW (n=2983)	12.3
AA (n=355)	35.5
Hispanic (n=440)	27.3
**Insurance**	
Private (n=3127)	13.7
Medicaid / Medicare (n=666)	28.5
**Family Structure**	
Two-parent (n=2623)	12.2
Single-parent (n=1126)	24.6

*Percents do not sum to 100 because only selected sub-groups of interest are shown

Adolescents with T1D have poor glycemic control, especially those socio-demographically at risk. In our SEARCH for Diabetes in Youth study (Mayer-Davis, SEARCH co-chair), among 3947 T1D youth, 17% had poor glycemic control (A1c≥ 9.5%) [12] and disparities were striking according to several socio-demographic characteristics.[13] (Table 1). Youth of minority race/ethnicity also had worse CVD risk profiles than non-Hispanic white (NHW) youth [14]. Youth using insulin pumps had the lowest A1C and fewest hospitalizations. However, youth of minority race/ethnicity and those from single parent households or from families with lower income, or whose parents had lower educational attainment were significantly less likely to be on insulin pump therapy (all p<0.001[15]). Worse health-related quality of life (HRQOL) was associated with an A1c value of at least 9%, more comorbidities[16], and with depressed mood [17]. Finally, minority youth and those with lower income reported more barriers to care than their counterparts.

Diabetes Management Requires High Levels of Adherence. Insulin delivery is either via multiple insulin injections daily or insulin pump technology[18, 19], informed by self-monitored blood glucose (SMBG). Average glycemia correlates inversely with the number of blood glucose (BG) checks daily [20, 21]. Mayer-Davis et al have shown that dietary intake of youth with T1DM is high in fat, and low in fiber, fruits and vegetables compared to recommendations [22]. Although higher physical activity has been associated with lower HbA1c and lower cholesterol [23], less than 50% of T1D youth meet current standards for physical activity[24]. Others have similarly shown sub-optimal health behaviors among youth with T1D[25 26]. **There is a substantial gap between ideal and actual adherence to the complex regimens required for optimal health in T1D.**

Attempts to improve adherence in adolescents through technology[27], education [28 29], specific counseling approaches[30] including motivational interviewing (MI) [31-33 7], and diabetes-specific behavioral family systems therapy (BFST-D)[34], have had mixed results or are not immediately applicable to clinical practice. Figure 1.1 showed our conceptual framework in which both motivational factors and information lead to acquisition of the skills needed for adherence to diabetes self-management recommendations. Further, a wide range of barriers to adherence exist that need to be addressed in a pragmatic manner, with the youth able to problem-solve on a real-world, day-to-day basis. The FL3X intervention is designed to address these key components, with an approach that takes into account contextual factors at play that also can impact adherence and outcomes.

Implementation as an Adaptive Intervention Sapienza and Masten [35] recently highlighted the importance of individual differences among youth in their response to stimuli, including interventions, and emphasized the need for research related to strategic timing and targeting of interventions. Adaptive interventions use *a priori* decision rules to define participants who, at pre-determined points during the course of the intervention period, are assigned to receive alternative treatment components. In this way, the intervention is designed to address the specific needs of those defined as "responders" or "non-responders" [36]. Initially, all participants are exposed to a "first-line therapy". Then, after a defined period of time, "tailoring variables" are measured and used to define the "responders" and "non-responders". Typically, responders are assigned to maintenance of the first-line therapy, while non-responders are assigned to some type of intensified therapy. This process mimics what commonly occurs in clinical practice (sometimes termed "stepped care" [37]). Adaptive interventions have been successfully implemented in the context of youth [38, 39] and adult behaviors [40-42] [43]. There are three major advantages to adaptive, rather than traditional fixed, interventions [44] . First, the adaptive intervention may reduce potential negative effects of an inappropriate intervention schedule. For example, for youth who are doing well, continuing the same intensity as that needed for other youth may result in boredom and may generate a negative view of the intervention. Second, adaptive interventions may optimize cost effectiveness by reducing unnecessary expenditures on intervention components that are not needed by youth doing well. Third, adaptive interventions may yield increased overall potency because youth who are not progressing well are identified efficiently and systematically for enhanced intervention. **The FL3X RCT will be the first rigorous evaluation of a formal, replicable <u>adaptive</u> intervention for self-management of T1D in adolescents.**

3. Study Objectives

Our objective is to rigorously evaluate the efficacy and sustainability of the innovative FL3X adaptive intervention by the conduct of a two-year randomized clinical trial (RCT). Coordinated from the University of North Carolina at Chapel Hill (UNC), the FL3X RCT will include a total of 250 individuals, randomized within each of 2 sites that participated in the FL3X Basic Pilot (University of Colorado Denver, Barbara Davis Center for Childhood Diabetes and Cincinnati Children's Hospital Medical Center, Division of Pediatric Endocrinology). To ensure at least 200 individuals at the end of the 18 month follow-up period we will recruit and randomize a total of 250 individuals. Participants randomized to intervention will receive the FL3X intervention. Those randomized to the control condition will receive their usual diabetes care. All participants will also receive a Health Summary that is comprised of key clinical (e.g., HbA1c, lipid panel, BP, weight status) and behavioral (e.g., dietary intake, physical activity) information collected during the standardized measurement visit at baseline, 6 and 18 months. Control participants will be encouraged to share this information with their providers and to go to their providers with any specific questions. Control and intervention participants will be followed for 18 months.

The primary outcome will be HbA1c as a measure of average glycemia over the prior 6-8 weeks. Secondary outcomes will include additional measures of glycemia, CVD risk profile, motivation, problem solving skills and health-related quality of life. Potential mediators include the diabetes self-management knowledge and behaviors and diabetes-related family communication. We will also evaluate the intervention cost and, from a societal perspective, cost effectiveness in terms of dollars per quality-adjusted life year gained.

4. Site Descriptions

The two clinical sites include 1) the Cincinnati Children's Hospital and Medical Center (CCHMC); 2) the Barbara Davis Center for Childhood Diabetes (BDC) at the University of Colorado-Denver. These sites provide access to patient populations with substantial diversity in terms of race/ethnicity, inclusion of patients with Medicaid/Medicare insurance and those living in urban or rural communities. Both CCHMC and the BDC are well established academic medical centers with vast experience in pediatric clinical research. Coordination of the project is accomplished from the University of North Carolina, Department of Nutrition in collaboration with the UNC Cecil G. Sheps Center for Health Services Research. Laboratory testing will be performed at the Northwest Lipid Metabolism & Diabetes Research Laboratories at the University of Washington in Seattle, Washington.

5. Selection & Recruitment of Subjects

Youth with a clinical diagnosis of T1D will be age 13-16 yrs. at study entry, with HbA1c $\geq$ 8 and ≤ 13 based on the most recent A1c in the medical record. Diabetes duration will be > 1 year. One primary caregiver who helps with diabetes management must also be willing to participate. Participants must not be planning to move within the next 18 months. Spanish-speaking youth and parents are eligible (typically youth will be bilingual, although a parent may speak only Spanish). Youth will be excluded if they have another serious condition that would render participation unwise, including serious physical or psychiatric conditions. Females who are pregnant will also be excluded. Recruitment will be targeted to ensure an appropriate range in HbA1c. We will aim to recruit 50% of participants with most recent clinical HbA1c of 8.0-9.0% and 50% with most recent clinical HbA1c of 9.1-13%. (The median HbA1c in SEARCH participants limited to our study inclusion criteria was 9.1%). Recruitment will use a combination of targeted mailings with phone follow-up and in-person clinic-based strategies. We established an enrollment goal of 250, given our end-of-study sample size (conservative) requirement of 200. This assumes 80% retention, based on a meta-analysis of Wifley [45] of retention in behavioral intervention studies in youth.

Medical records will be used to identify patients who potentially meet the eligibility criteria for this study. Medical records will also be used to collect information related to the adolescent's diabetes care, as well as demographic information.

Strategies to Optimize Recruitment and Retention Individual sites will be responsible for recruitment. Recruitment involves providing and promoting awareness of the existence of FL3X to clinic physicians, staff and other health care professionals caring for individuals with diabetes who meet eligibility criteria at that site. The primary recruitment strategy will be to send an informative recruitment letter to parents of eligible youths, followed by a phone call by FL3X research staff. Recruitment materials will also be prominently displayed in the pediatric diabetes clinic waiting area and exam rooms. The FL3X website (www.type1fl3x.org), study recruitment posters and brochures can be used as recruitment aids for interested parties. FL3X study personnel may also utilize in-person recruitment of eligible participants during their usual care visits. Social media may also be used to promote awareness of FL3X.

The recruitment process will include a series of conversations with potential participants and their parents, should they be interested in participating. Following the initial mailing, study staff will follow-up with a brief telephone call to ascertain interest in participation. If there is interest, the staff person will briefly explain the study. The staff will also set-up a second telephone call to discuss the study in more detail with both the parent and participant individually. The parent and participant will be sent an additional document detailing "Frequently Asked Questions" about the FL3X study. This document will include items that may be frequent barriers or issues which could impede participation in the study. The staff will review this document and specific barriers or concerns for the potential participant during the pre-arranged 2nd phone call. The conversation will be individualized to the specific potential participant and will incorporate strategies of motivational interviewing as a way to allow the potential participant to identify, express and discuss any concerns about participation. This two-step recruitment process will help ensure the participant/parent understand FL3X fully before deciding to participate.

FL3X will aim to retain 90% of enrolled participants. The successful approach used in the R21 will be used including participation incentives for attending the standardized measurement

visits ($120 for baseline, $200 for 6-month, and $250 for 18-month; and $50 for the brief visits at 3 and 12 months). Participants will receive an additional incentive for completion of CGM data collection at baseline, 6 months, and 18 months ($50 for wearing the CGM device and $25 for returning the device). Parents will receive a $20 incentive for each of the 5 measurement visits. From vast experience in the SEARCH study, we know that maintaining updated contact information is the key to retention, particularly for youth living in low SES settings. We will update contact information at each in-person visit. FL3X participants will also continue with usual diabetes care within their clinical practice so updated contact information may be obtained from clinical sources as well. Home visits or travel incentives will be offered if transportation is a barrier to completion of the standardized measurement visits.

6. Process of Obtaining Consent

Consent will be obtained following the two-step recruitment process detailed above. For participants who agree to participate and schedule a baseline visit, the study consent/assent form will be mailed to the participant and parent prior to the scheduled baseline visit. At the time of the visit, study staff will review the consent in detail before standardized data collection begins. The study participant and parent will be given as much time as needed to review the consent/assent form and ask any questions prior to signing the consent/assent form. Participation in this study is voluntary and the participant is free to withdraw their consent/assent and discontinue participation in this research at any time. If a participant withdraws from the study, they will have the choice as to whether or not they allow the study team to use all information gathered in future analysis.

Following consent, participants may be involuntarily terminated from the study if they develop a serious condition that would render participation unwise, including serious physical or psychiatric conditions. Females who become pregnant may also be excluded from study participation.

7. Randomization

Randomization will be managed within the study website, with oversight from UNC, Chapel Hill. Participants will be randomized at the level of the individual, stratified by site, then by HbA1c categories (8.0-9.0% and 9.1-13.0%) using a block size of 4 within each of the strata. Participants will be assigned to intervention or control groups according to a preset schedule based on date of enrollment in a 1:1 ratio. Upon completion of the baseline standardized measurements, the randomization assignment will be opened by the research staff and the participant will be informed as to whether they have been assigned to the FL3X intervention or control. For those assigned to intervention, the first intervention visit will be scheduled to occur within 1-2 weeks, and the intervention delivery schedule will proceed as shown in Table 9.1 below. Both intervention and control participants will be scheduled to return for repeat standardized measurements 3, 6, 12, and 18 months post-randomization.

8. Standardized Measurements

FL3X Standard Measures capture data on the primary outcomes, secondary outcomes, potential mediators, cost effectiveness, intervention acceptability and a variety of other subjects. Specific details are outlined below. A summary of standard measures are shown in tables 1-3 below. The baseline study visit is expected to take approximately 2 hours. Participant forms are expected to require about 60 minutes and Parent forms are expected to require about 50 minutes to complete.

8a. Standardized Measurements for Primary Outcome *The primary outcome* will be HbA1c as a measure of average glycemia over the prior 6-8 weeks [46,47]. Although this variable has the limitation of not quantifying episodic hypo- or hyperglycemia, it has been used in a large number of clinical trials aimed at demonstrating efficacy of lowering HbA1c to reduce risk for chronic complications of diabetes [48, 49 50 51] and in studies examining efficacy of interventions to improve diabetes self-management [52 53 54 55].

8b. Standardized Measurements for Secondary Outcomes FL3X will include additional measures of glycemia, glycemic variability and time with hypoglycemia (≤70 mg/dl, ≤60 mg/dl, ≤50 mg/dl) and hyperglycemia (via CGM), CVD risk profile, quality of life, motivation, intention, and problem solving skills. Secondary outcomes derived from CGM data will include number of times per day with hypoglycemia (≤70 mg/dl, ≤60 mg/dl, ≤50 mg/dl), total time spent in hypoglycemia per day based on area under the curve summed across each occasion, and percent of days monitored in which criteria were met at least one time). Analyses will use data derived from “acceptable days,” defined as days in which at least 75% of readings are available for that day. Additional outcomes derived from acceptable days will include time spent in hyperglycemia [> 180 mg/dl (or > 150 mg/dl)] based on area under the curve for each occasion summed over the day, number of occasions of hyperglycemia per day, time spent in range per day, mean daily glucose, mean daily peak glucose, mean daily glucose nadir, number of occasions of hypoglycemia from 11pm to 7am, and percent of time in hypoglycemia from 11pm to 7am. Telephone 24-hour dietary and physical activity recalls will be collected from a sample of study participants wearing the CGM devices in order to ascertain dietary intake, insulin bolusing, and physical activity. CVD risk profile will include weight status, blood pressure and lipid profile. Weight status will be based on age- and gender-specific body mass index z-scores [56] using height and weight measured according to the SEARCH study protocol (available online, www.searchfordiabetes.org); blood pressure and natural waist measurements will also be measured using the SEARCH protocol. General quality of life will be measured using the PedsQL 4.0 generic core module for youth and parents, also implemented in SEARCH [57] Diabetes specific quality of life will be measured using the Pediatric Diabetes Quality of Life Scale (PDQ) developed by Wysocki et al. Motivation and intention will be measured using a 10 point scale, modified from an asthma intervention done by Seid et al [58]. Problem solving will be measured by the Social Problem-Solving Inventory-Revised :Short (SPSI-R:S). Diabetes related eating problems will be assessed using the Diabetes Eating Problem Survey (DEPS-R). Self-efficacy and outcome expectations will be assessed using scales developed by Iannotti. [59 60]

8c. Potential mediators include the Diabetes Self-Management Profile: Self-Report (DSMP-SR)[61-64 65], diabetes-specific family conflict [66] and shared responsibility[67], and diabetes knowledge (Diabetes Knowledge Assessment). We will administer the Hypoglycemia Fear Survey (HFS) [68 69, 70], and general health habits (e.g., sleep, tobacco) using SEARCH methods. Food security issues will be assessed through use of the USDA Food Security questionnaire.

8d. Intervention Acceptability For intervention participants' only at 18 months, subjects and their parents will be individually interviewed using a semi-structured format, regarding intervention acceptability. The interviewer will be someone other than the FL3X Coach who was the interventionist. Subjects will be told that the interview is to help us to improve the intervention and will be asked to comment on each part of the intervention (length, format, content, phone calls, messaging, alerts, etc.), suggesting aspects that could be improved, as well as noting aspects that are acceptable as is. These interviews will be documented using audio-recordings and extensive notes.

8e. Cost Effectiveness *Measures required by cost and cost effectiveness analyses* will be based on the surveys and time logs employed by the TODAY study [71] . Relevant costs include those related to the development, provision, and adherence of the FL3X and control conditions. Specific costs will include training of study personnel and personnel costs related to the provision of the treatment arms (including direct time with participants, preparation for visits, and time for other participant contacts (e.g., text messaging). Participant (adolescent and parent) costs will also be assessed, including direct medical costs (e.g., strips for BG testing), and health care utilization including emergency department and hospital visits for DKA or severe hypoglycemia. We recognize the potential limitations of self-reported data. However, Ritter et al [72] found that recall error is similar between individuals in intervention and control groups, thus systematic bias is unlikely. To evaluate the self-reported data, we will conduct a medical record review on a 10% sample of participants for medical care received in the 6 months prior to the study visit. Direct, non-medical costs (e.g., fees for participation in sports for participant, parent costs related to participation such as travel) will be assessed. Indirect costs will also be considered: absenteeism and loss of earnings related to intervention visits [73]. The cost-effectiveness analysis will also require a standard preference-based QOL measure (to identify change in quality adjusted life year (QALY)) as a way to express benefit. QALYs will be determined from the administration of the Health Utilities Index (HUI) Mark 3. The HUI was developed for use in children and is an accepted instrument for economic analyses [74]. The HUI will be completed by the parents (providing a proxy assessment of their child's general health attributes).

8f. Other We will also request contact information, demographics, health history and diabetes care information from the participants and their parents. We will administer the Center for Epidemiologic Studies-Depression (CES-D) scales [75] and have the participants self-report tanner stage. Control participants and their parents will be individually interviewed using a semi-structured format, regarding recruitment and retention. These interviews will be documented using audio-recordings.
We will also administer two questionnaires to understand impulsivity and eating behavior, including the short version of the Barratt Impulsivity Scale (BIS-15) will be used to assess trait impulsivity (15 items, 2-3 minutes).[1] and the full Dutch Eating Behavior Scale (33 items, 10 minutes)[2] to measure three different eating behaviors: emotional eating (eating in response to emotional arousal states such as fear, anger or anxiety), externality (eating in response to external food cues such as sight and smell of food), and restrained eating behavior/cognitive restraint (conscious efforts to limit and control dietary intake).[2] Note that External Eating is measured at different time points than Emotional Eating and Restrained Eating.

Additionally, to understand the relationships between dispositional mindfulness, impulsivity, self-efficacy for diabetes self-management, and diabetes self-management, dispositional mindfulness will be measured with the Child and Adolescent Mindfulness Measure (CAMM), a 10-item, single-factor self-report questionnaire.

Table 8.1 Overview of Standardized Measures for Parents
(forms self-administered unless otherwise noted)

**Parent**					
	**Baseline**	**3 mos**	**6 mos**	**12 mos**	**18 mos**
**Contact Information**	X	X	X	X	X
**Peds QL – Generic Quality of Life**	X	X	X	X	X
**Diabetes Self-Management Assessment (DSMP-SR)**	X	X	X	X	X
**Diabetes Family Conflict Scale**	X	X	X	X	X
**Diabetes Family Responsibility**	X	X	X	X	X
**Diabetes-Specific Quality of Life (PDQ)**	X	X	X	X	X
**Specific Health Care Use** *(Interviewer Administered)*	X		X	X	X
**Low Blood Sugar Survey**	X		X		X
**FL3X Cost Survey** *(Interviewer Administered)*	X		X		X
**Demographics** *(Interviewer Administered to parent and participant)*	X		X		X
**Health History and Diabetes Care**	X		X		X
**Food Security Questionnaire**	X				X
**Intervention Acceptability Interview** (for intervention group only)					X
**Recruitment/Retention Interview** (for control group only)					X

Cells shaded in black denote measure not completed at that time.

Table 8.2 Overview of Standardized Measures for Participants					
**Participant**					
	**Baseline**	**3 mos**	**6 mos**	**12 mos**	**18 mos**
**Specimen Collection Form**	X	X	X	X	X
**HbA1c**	X	X	X	X	X
**Lipids**	X		X		X
**Continuous Glucose Monitoring**	X		X		X
**Physical Exam**	X		X		X
**Intention and Motivation**	X	X	X	X	X
**Social Problem-Solving Inventory-Revised : Short** *(In person only)*	X	X	X	X	X
**Peds QL – Generic Quality of Life**	X	X	X	X	X
**Diabetes Self-Management Assessment (DSMP-SR)**	X	X	X	X	X
**Diabetes Family Conflict Scale**	X	X	X	X	X
**Diabetes Family Responsibility**	X	X	X	X	X
**Diabetes-Specific Quality of Life (PDQ)**	X	X	X	X	X
**Outcome Expectations for Diabetes Self-Management**	X	X	X	X	X
**Self-Efficacy for Diabetes Self-Management**	X	X	X	X	X
**Health Utilities Index (Mark 3)**	X		X		X
**Diabetes Knowledge Assessment**	X		X		X
**Low Blood Sugar Survey**	X		X		X
**Diabetes Eating Problem Survey**	X		X		X
**Health Behaviors and Home Environment**	X		X		X
**Health History and Diabetes Care**	X		X		X
**Dutch Eating Behavior Questionnaire (DEBQ) External Eating**	X		X		X
**Depression symptoms (CES-D)** *(In person only)*	X	X	X	X	X
**Tanner Stage**	X		X		X
**24-hour Diet & Physical Recall** *(Telephone recall)*	X		X		
**Intervention Acceptability Interview** (for intervention group only)					X
**Barratt Impulsivity Scale (BIS-15)**				X	X
**Dutch Eating Behavior Questionnaire (DEBQ) Emotional and Restrained Eating**				X	X
**Child and Adolescent Mindfulness Measure (CAMM)**				X	X

Cells shaded in black denote measure not completed at that time.

Table 8.3 Overview of FL3X standardized measurement visits for Staff	
**Forms**	**Notes**
**Staff Time Survey**	Staff will record for periods of 1 week, starting 4 months after study initiation, and continue every 4 months for the

	first two years, then every 6 months for the remainder of the study
**Intervention Encounter forms**	Coaches will complete an encounter form for each intervention session (whether missed or completed) in order to track session completeness and content.

9. FL3X Intervention

9a. Importance and Relevance The FL3X Adaptive Intervention is designed to increase adherence to diabetes self-management including medical management (blood sugar testing and insulin dosing), diet, and physical activity. FL3X relies on motivational interviewing, and problem-solving skills therapy as the basis for the counseling strategy, and creates a coherent integration across three key components of 1) behavior family systems therapy focused on family communications and teamwork; 2) individualized diabetes education in response to knowledge gaps relevant to behavioral goal attainment; and 3) use of currently available communications technology to support behavioral goal attainment through participant-defined reminders and motivational boosters, and/or peer support.

Expected outcomes include improved HbA1c, CVD risk factors, and quality of life, mediated by improved diabetes self-management behaviors, and improved family communications related to diabetes. Further, as done in the FL3X Basic pilot, intervention delivery is possible in the setting of an active diabetes clinic, by trained diabetes educators who do not need to be experienced psychotherapists.

Delivering the intervention from the participant's usual diabetes care provider's office effectively extends the impact of the health care team to support the participant's day-to-day actions to manage blood sugar, to optimize CVD risk profile, and to do so with improved quality of life. Because of the individualized nature of the MI process, the intervention is designed to be culturally relevant across a broad range of race/ethnicity, gender, socio-economic status and region.

9b. Adaptive Design All FL3X intervention participants will be encouraged to continue their usual diabetes care. In addition, they will receive "FL3X-Basic", which is the initial 3-month intervention that includes 4 sessions (40-60 min), supplemented with short additional contacts (via text, email, or web-based communication) as needed. Thereafter, applying principles of adaptive interventions, based on a decision rule using HbA1c values measured at defined intervals, participants are iteratively assigned to "FL3X-Check-in" or "FL3X-Regular", both of which continue with MI and PSST for the underlying counseling strategy. In FL3X-Check-in, participants who are doing well ("responders") will receive minimal ongoing support to reinforce successful strategies through brief monthly "touch-base" contacts. FL3X-Regular will include participants not meeting the A1c targets ("Regular-responders"). They will continue to have a minimum of 3-4 in-person full-length sessions (40-60 min) over a 6-month interval, with additional brief contacts as needed (e.g., text, voice, or internet). These sessions will utilize modules that expand upon the three key components of FL3X-Basic (BFST, individualized diabetes education, and communication technologies in support of behavioral goal attainment) and additional brief contacts as needed (e.g., texting). This approach allows implementation of

the best of the proven strategies, specific to the needs of individual adolescents living with T1D, when the need exists.

Responders will be defined as those whose A1c meets the target at the next measurement visit. The target HbA1c is based on the most recent A1c and is re-defined at each measurement visit. The target is reached if any of the following criteria are met: 1) HbA1c is $\leq$ 7.5%; or 2) previous HbA1c was > 7.5% and $\leq$ 9.0% with a decrease of $\geq$ 0.5%; or 3) previous HbA1c was > 9 with a decrease of $\geq$ 1.0%. This is consistent with ADA clinical practice guidelines [76] and with other clinical trials that use HbA1c change of $\geq$ 0.5 in youth with T1D as a marker of success [77]. Measurements will occur as part of the study standardized measurement schedule (Tables 8.1 & 8.2) and so tailoring will occur for the first time shortly following delivery of FL3X Basic (at 3 months post intervention initiation), then again at 6 and 12 months.

The overall interventions schedule is given in Table 9.1.

FL3X Basic FL3X Basic is the first-line treatment and is designed to address a variety of specific barriers to adherence (e.g. social support, education, family issues, specific concerns such as needle anxiety, pragmatics of avoiding missed insulin doses and insulin dosing decisions). Four sessions (each ~40-60 min) are delivered over three months, generally with the second session occurring about 2-3 weeks after the first session. Core content of each session is given in Table 9.1.

FL3X Check-in FL3X Check-in will be assigned for youth classified as "Responders". Upon assignment to FL3X Check-in, an updated Health Summary will be reviewed with youth, based on the most recent standardized measurements taken. Youth will be encouraged to continue with the problem-solving processes and strategies they have found to be helpful and to continue to identify and implement approaches to address barriers. There will be monthly "touch base" contacts made by the coach using telephone or internet (Skype) depending on what the participant prefers. The MI counseling style will continue through these brief contacts. Additional such contacts can occur as needed (e.g., texting for support or quick questions).

FL3X Regular FL3X Regular will be assigned for youth classified as "Regular-Responders". These participants will have a minimum of 3-4 in-person full-length sessions (40-60 min) over each 6-month interval, with additional brief contacts as needed (e.g., text, voice, or internet). Full-length sessions will typically be in-person, however as we learned from FL3X R21, Skype is a viable alternative particularly for youth who live a considerable distance from the clinical site. FL3X Regular is designed to address identified barriers to adherence in more depth. This increased focus will be accomplished through use of modular 'toolboxes.' The coach will review data obtained during the most recent standardized measurement visit related to family communications (Diabetes Family Conflict Scale), the Diabetes Self-Management Profile, the Pediatric Diabetes Quality of Life scale, and the Diabetes Knowledge Assessment, along with the updated Health Summary. Informed by this material, the coach and participant will collaboratively determine the specific direction for the upcoming FL3X Regular sessions. Modules can be selected from the Toolboxes (see below) related to the following four areas: 1) family communication and teamwork; 2) diabetes education; 3) social support; and 4) diet and physical activity.

Session-by-session intervention outline The intervention will be conducted separate from normal diabetes clinic visits. Presented below is a brief outline of intervention described as specific visits.

Table 9.1. Behavioral Strategies to be Delivered by FL3X Coach

Baseline standardized measurements – (week 0)
FL3X Basic – All intervention participants
Session 1- (40-60 minutes, week 1)
* *Teen and Parent together:* Establish rapport and provide overview of intervention approach and explain confidentiality<br>* *Teen Alone: Provide FL3X Guide to Living* Elicit teen's perceptions of current functioning and self-management barriers based on PDQ. Use brief MI script and FL3X Guide to 1) aid teen in creating a frame for motivation and to begin thinking about change, 2) introduce Bright Ideas problem-solving framework, and 3) (if appropriate) guide the teen in utilizing the problem-solving framework to select a goal, specify the behavior(s) targeted for intervention, to develop a behavior change plan, and to define the implementation of the plan and its ongoing evaluation on a written problem-solving worksheet<br>* Establish cell phone calendar reminders or motivational boosters as appropriate.<br>* Negotiate a behavioral homework activity/exercise related to the use of the problem-solving guide and worksheet<br>* *For Parent:* provide materials to read including *FL3X Guide to Living* and *How You Can Help Brochure* while teen is engaged in the intervention session. Materials will orient parent to the intervention and relate to family communications, MI, and problem-solving skills.
Session 2- (40-60 minutes, week 3)
* *Teen alone:* Review and advance problem-solving skills from Session 1<br>* Revise or refine specification of goals and target behavior(s)<br>* Continued use of Agenda Setting, Asking Permission, Open-Ended Questions, Reflecting, and Summarizing<br>* Teen and Parent together: Enlist parent in supporting teen's change efforts by integrating parent into intervention. Identify ways the parent can support teen. Use BFST tools to specify what 'support' means….a) how to be part of the diabetes team b) role-play common communication techniques c) use technology to improve teamwork and communication d) Bright IDEAS PSST (see *Supplemental Material 2-8* for materials)<br>* Negotiate a behavioral homework assignment related to the communication training materials such as self-assessment of positive and negative communication interaction.
Session 3- (40-60 minutes as needed, week 8)
* *Teen only:* Review and advance problem-solving/MI skills from behavioral homework in Session 2. Conduct target review of original motivation to change. Review FL3X brochure page 3 feedback and Heath Summary to ensure progress is being made toward participant defined goals/objectives. Revise or refine specification of goals and target behavior(s).<br>* *Teen and Parent:* Assess for communication conflict and teamwork. Depending on assessment, will implement one of three interventions: (1) if there is conflict, complete family communication strategies, (2) if there was little conflict but also not successful at completing behavioral goal, then complete enhances PSST together, or (3) if parent and child appear to be working as a team and met the behavioral goal with no conflict, then implement intervention elements using the Toolbox resources more in-depth. Consider a referral if the families in #1 continue to have significant conflict that is not responding to FL3X strategies.<br>*Negotiate a behavioral homework assignment related to the communication training materials such as self-assessment of positive and negative communication interaction.
Session 4 (40-60 minutes as needed, week 12)
**Teen only:* Review and advance problem-solving/MI skills from behavioral homework in Session 3.<br>**Teen and Parent:* There are two options of interventions based on whether the family received the Toolbox resources last time or not: (1) If the parent and child dyad did not receive the Toolbox information last time (i.e., had conflict or unsuccessful behavioral change achievement), they receive Toolbox resources more in-depth this time. (2) If the parent and child dyad did receive the Toolbox resources more in-depth last time, then enhance joint PSST.<br>*Negotiate a behavioral homework assignment related to the communication training materials such as self-assessment of positive and negative communication interaction.<br>* Determine schedule for upcoming optional sessions. If participant opts out, establish a time for placing a follow-up telephone call in approximately 3 weeks' time

Follow up standardized measurements – (month 3)	
FL3X Check-In – Responders	FL3X Regular - Non-Responders
* Months 4, 5, 6<br>* Conduct monthly "touch base" outreach (~15min.)<br>* Additional outreach via text, phone or internet if needed (youth preference)<br>* Encourage youth to continue with problem solving processes and strategies. Continue to identify and implement approaches to address barriers.	* Months 4, 5, 6<br>* In-person sessions (40-60min. per month)<br>* Continue working on family communication, PSST, education, or diet/physical activity<br>* Revise or refine specification of goals and target behavior(s) with toolbox modules as needed based on participant standardized measurement responses and the Health Summary.
Follow up standardized measurements – (month 6)	
FL3X Check-In – Responders	FL3X Regular - Non-Responders
* Months 7, 8, 9, 10, 11, 12<br>* Conduct monthly "touch base" outreach (~15min.)<br>* Additional outreach via text, phone or internet if needed (youth preference)<br>* Encourage youth to continue with problem solving processes and strategies. Continue to identify and implement approaches to address barriers.	* Months 7, 8, 9, 10, 11, 12<br>* Monthly contacts with a minimum of 3-4 in-person full-length sessions (40-60 min * Continue working on family communication, PSST, education, or diet/physical activity<br>* Revise or refine specification of goals and target behavior(s) with toolbox modules as needed based on participant standardized measurement responses and the Health Summary.
Follow up standardized measurements – (month 12)	
FL3X Check-In – Responders	FL3X Regular - Non-Responders
* Months 13, 14, 15, 16, 17, 18<br>* Conduct monthly "touch base" outreach (~15min.)<br>* Additional outreach via text, phone or internet if needed (youth preference)<br>* Encourage youth to continue with problem solving processes and strategies. Continue to identify and implement approaches to address barriers.	* Months 13, 14, 15, 16, 17, 18<br>* Monthly contacts with a minimum of 3-4 in-person full-length sessions (40-60 min * Continue working on family communication, PSST, education, or diet/physical activity<br>* Revise or refine specification of goals and target behavior(s) with toolbox modules as needed based on participant standardized measurement responses and the Health Summary.
Post-intervention standardized measurements – (month 18)	

9c. Intervention Components

Motivational Interviewing MI [78] is a collection of patient-centered interviewing techniques that can be used as the context for virtually any psychological or behavioral intervention. While MI is not itself an intervention, it can bolster the effectiveness of behavioral and psychological interventions by promoting the client's engagement in the behavior change process and motivation for change.

MI is a patient-centered method of counseling designed to create a collaborative environment in which patients can develop arguments for change, understand and resolve their ambivalence to change, develop strategies for change, and increase their confidence in their coping abilities [78, 79]. The goal of MI is to help individuals express their own reasons for change

or lack of change and understand how their behavior might affect achievement of their goals. In this way, MI is useful in helping patients to create a motivational frame – an appreciation of the need to change, an understanding of why they want to change, and a strategy for change. MI recognizes that how intervention content is provided to clients and patients is as important as actual intervention content [80]. MI uses non-judgmental and non-confrontational communication [78]. There are four general principles of MI: express empathy (e.g., acceptance facilitates change), develop discrepancy (e.g., let the client present arguments for change), roll with resistance (e.g., avoid arguing), and support self-efficacy (e.g., belief in the possibility of change is an important motivator) [78]. Because MI emphasizes a collaborative process in which people are active participants, it ensures cultural appropriateness and acceptability for adolescents with chronic conditions, who tend to respond more positively to a motivational approach [81-85] and to prefer an interactive versus didactic style [86].

In the proposed intervention, MI is expected to help the adolescent to create a motivational frame and, thus, to increase the probability that the intervention content (problem-solving skills, diabetes education as needed) will be well received and used effectively by adolescents.

MI Training Program All FL3X coaches who will implement the intervention in this study will receive extensive training in the MI approach. FL3X coaches will be trained by a highly experienced, certified MI Trainer. Coaches will be taught how to effectively use specific motivational enhancement skills and techniques to work effectively with adolescents with poorly controlled T1D. MI techniques and interventions will be demonstrated using instructional discussions, participatory role-playing, videotaped clinical vignettes, case material, and fish bowl exercises. Examples of correct and incorrect skills will be presented and contrasted. Motivational techniques can be used to enhance the participant's commitment to change. FL3X coaches will practice using these tools in a motivationally enhancing manner. FL3X coaches will learn how to use decisional balancing to evaluate and promote readiness for change and how to roll with resistive individuals.

Problem-Solving Skills Training In managing a chronic illness on a daily basis, individuals face a variety of challenges for which there are no single, standard remedies and for which they must identify and implement solutions [87]. Problem solving is the behavior process through which individuals accomplish this [88, 89]. While disease-specific knowledge is necessary for this kind of self-management [90-92], it is not enough [93]. Problem-solving skills enable individuals to manage daily barriers to adherence and to make appropriate adjustments to the self-care regimen [94]. Thus, problem-solving is the process by which patients translate knowledge into action for disease management on a daily basis [95]. In pediatrics, problem-solving interventions have been widely used with parents of children with chronic health conditions [96-100]. Among adolescents with T1D, problem solving interventions have been used to improve self-efficacy [101], metabolic control through increased frequency of blood glucose testing [102], anxiety, stress, and coping [103], and quality of life [104, 105].

Participants will receive training in problem-solving based on methods used in previous studies by Dr. Seid and Dr. Wysocki. Problem-solving skills building uses a structured framework that includes problem definition, goal setting, generation of possible solutions, negotiated selection of a consensus problem solving plan often in the form of a behavioral contract, planning of implementation of the problem-solving plan and identification of likely barriers, putting the problem-solving plan into action, evaluating the effectiveness of the plan and negotiating refinements to the plan as needed.

FL3X Basic materials were based on Varni's 'Bright Ideas' problem-solving skills training (an extension of Nezu and D'Zurilla's problem-solving skills intervention) and D'Amico's Project CHOICE MI intervention, as integrated in Seid's *In Vivo* intervention for adolescents with asthma [106]. Concepts and materials are consistent with the MI process, to support participants in identifying and implementing their own agenda for change. During FL3X Basic Session 1, A "Wheel of Change" will be introduced first as a way to normalize the change process for participants. Participants will be shown the wheel and described the process of change as it is stated according to the Transtheoretical Model/Stages of Change used to assess behavior change readiness. It states that change occurs in a series of steps from not identifying the need to change (pre-contemplation) to performing the behavior routinely (maintenance). As part of exploring change, participants will be given the opportunity to assess their current attitude toward change using self-efficacy rulers. These rulers represent the participant's willingness and confidence to make changes that will positively impact their diabetes management. These rulers fall on a 1-10 scale where 1 represents not at all willing/confident and 10 represents master level implementation. The Bright IDEAS concept will be used next to present PSST as it provides an easy way to conceptualize the problem solving process: identify, define, evaluate, act, see (IDEAS).

Behavioral Family Systems Therapy Considerable research demonstrates the important role of family communication, parent-adolescent teamwork and cooperative problem solving in effective family management of T1D [107-111.] Parents perceived to be over-involved in diabetes care, and adolescent-parent disagreement regarding responsibility for diabetes care have been associated with poor metabolic outcomes among adolescents with T1D [112]. Also, parent responsiveness was associated prospectively with diabetes-related quality of life among youth with T1D[113] . An intensive, home-based family psychotherapy approach was found to improve metabolic control among T1D youth with chronically poor metabolic control, however the intervention effect was attenuated among those who were overweight, perhaps reflecting the need to address underlying behavioral and physiologic factors (e.g., insulin resistance) related to overweight, particularly for minority T1D youth [114].

Wysocki et al conducted two randomized controlled trials of Behavioral Family Systems Therapy for Diabetes (BFST-D), a family communication and problem solving training intervention that incorporates elements of behavior therapy and systemic family therapy. In the first trial [115-118] significant and durable treatment effects were obtained in favor of BFST-D on measures of family conflict, communication skills and parent-adolescent relations, but these benefits did not yield corresponding improvements in glycemic control or treatment adherence. In the second trial of a revised intervention that included more diabetes-specific components [119-121], significant BFST-D treatment effects were obtained on A1c levels and treatment adherence.

Parent-adolescent teamwork and cooperative problem solving to facilitate the youth's behavioral goal attainment is based on empirical evidence and is further supported by qualitative feedback from our own focus group work. As part of the FL3X intervention, adolescents along with the parent(s) who provide support to diabetes self-management will receive a condensed exposure to the fundamentals of healthy parent-adolescent communication based on the materials and procedures employed successfully in Dr. Wysocki's trials of BFST-D [116, 122-124 125-127]. Our approach respects the adolescent as he/she progress towards independence in diabetes self-management, while facilitating the important positive support that parent(s) can provide in support of the youth's behavioral goal attainment.

Communication training includes standard behavior therapy techniques of instructions, feedback, modeling, rehearsal and individualized behavioral homework. Implementation will

pay particular attention to parental support of the goals established by the adolescent to improve diabetes self-management.

Toolboxes. Materials are available to support content areas across FL3X Basic, FL3X Check-in and FL3X Regular. The most intensive use of Toolbox materials will be during FL3X Regular. Toolbox content is briefly described below. All specific information related to diabetes self-management will be consistent with American Diabetes Association clinical practice guidelines [128]. The toolboxes include materials that are expected to be needed commonly by study participants, but may also include materials that are consistent with community and clinical practice materials utilized locally. Referrals to appropriate health professionals are made as needed if the scope of a problem is beyond the expertise and capacity of the FL3X coach.

- *Family Communications Toolbox*. This toolbox includes specific components from the BFST-D intervention of Wysocki et al [129-131]. Consistent with implementation of the original BFST-D, modules within the Toolbox will be selected by the coach based on individual youth and family needs. Modules include activities designed to address family-based problem-solving skills and family communication, including family systems issues such as cross-generational coalitions (parents taking sides with the teen against the other parent), hierarchical reversal (teen holds power over parents via misbehavior), and triangulation (family member caught between two other family members).
- *Diabetes Education Toolbox*. There are four components to this Toolbox. First, materials are available from the widely-used and recently updated (Chase and Maahs) Pink Panther series, developed at the UCD-BDC site [132] [133]. Second, a series of vignettes are provided related to pragmatics of BG management; these provide a basis for problem-based learning and reinforcement of skills (e.g., insulin dosing decisions). Topics range from basics of insulin regimens, essentials of carbohydrate counting, and sick day guidelines, to more complex topics related to use of correction factors, use of specific functions on insulin pumps, etc. Third, information regarding reliable websites and other available resources can be provided (e.g., ADA website). Fourth, technologies that can support diabetes education will be identified (e.g., cell phone apps to assist with insulin dosing), facilitated by the UNC CDTR Access with Technology Core.
- *Social Support Toolbox.* Based on youth feedback, capacities and activities will include: 1) guidance for participants to use problem solving skills to identify a Diabetes Buddy from among current peers or family members, and determine the specific role the Buddy will play in their diabetes self-management; 2) referral to reliable online resources, such as JDRF or the Type 1 Exchange GLU, for further social support; 3) guidance for use of cell-phone messaging to include communications with the coach, family members or friends, or use of messages “from you to yourself” as reminders or motivational support.
- *Diet and Physical Activity Toolbox.* This toolbox addresses issues of adherence to healthy diet and physical activity recommendations beyond glycemic control. Weight management and heart healthy lifestyle habits are the focus of this Toolbox. Supports to diet include guidance on selection of healthy foods and avoiding energy dense foods considering settings such as the school cafeteria and fast food restaurants. Supports to physical activity include information on local capacities (e.g., community and school sports teams), guidance on use of pedometers, on-line or other technology-based supports to increasing physical activity (e.g., computer games that involve physical activity such as Wii Fit, should the participant choose to purchase such items on their own). All materials will be modified to address the need to integrate daily blood glucose management with longer term health objectives for youth with T1D.

Fidelity All full-length intervention sessions will be audio-taped. As done in the Pilot, intervention fidelity will include: 1) assessment of adherence to MI principles using the Motivational Interviewing Treatment Integrity (MITI) system[134]; and 2) review of content fidelity using a content checklist. Throughout the intervention, the first 2 of each session type in FL3X Basic (i.e., session 1, 2, 3, 4) by each coach and a random 10% sample of all sessions thereafter for each coach, will be reviewed with feedback to all coaches provided in a timely fashion.

10. Control Condition

Those randomized to the control condition will receive their usual diabetes care. Control participants will also receive a Health Summary that is comprised of key clinical (e.g., HbA1c, lipid panel, BP, weight status) and behavioral (e.g., dietary intake, physical activity) information collected during the standardized measurement visit at baseline, 6 and 18 months. *(Please note: both intervention and control participants receive the same Health Summary document after the major measurement).* They will be encouraged to share this information with their providers and to go to their providers with any specific questions.

11. Extended Follow-up

11a. Additional Study Aim To explore the durability of the FL3X intervention effects up to 30 months post-randomization. Upon completion of the end-of-study 18-month measurement visit, a subset of participants will be invited to continue with extended follow-up. In order to allow for adequate time to analyze study results, all extended follow-up visits will need to be completed by January 2018. Therefore, eligibility for extended follow-up will be based on the participant's target date for the 18-month visit as follows:

- Target date before January 1, 2017 – eligible for two follow-up measurement visits at 24 and 30 months post-randomization
- Target date after December 31, 2016 and before July 1, 2017 – eligible for one follow-up measurement visit at 24 months post-randomization
- Target date after June 30, 2017 – not eligible for extended follow-up

Upon completion of the 18-month study visit, eligible participants and their parents will be invited to continue with extended follow-up. There will be 3 follow-up groups:

- Current Control – will remain as Control
- Current Intervention – approximately 50% randomized to follow-up intervention/coaching
- Current Intervention – approximately 50% randomized to no follow-up intervention/coaching

Current Intervention participants who agree to the extended follow-up will be re-randomized to continued FL3X coaching or no continued coaching. Continued coaching will entail quarterly coaching sessions (FL3X regular or FL3X check-in, following the same adaptive decision rules as for the main trial) until their final measurement visit.

At the 24-month follow-up visit, all participants will have the following assessments: HbA1c, quality of life, intention and motivation, problem solving, family conflict, diabetes self-management and symptoms of depression. Those participants who are eligible for two follow-up visits will have these same assessments performed at the 30-month visit as well.

11b. Consent Teen/Parent consent for extended follow-up will be obtained following a two-step recruitment process similar to the process used for initial recruitment into the study. A study staff member will call the parent (or teen if >18 yrs. of age) approximately 3 weeks prior to the 18-month measurement visit to invite the parent and teen to participate in the extended follow-up. The staff member will explain the purpose of extended follow-up, the requirements for the 24- and 30-month (if applicable) measurement visits, and the group assignments. Parents of Intervention participants will be given an explanation of the randomization process and what it would mean to be randomized to additional coaching sessions vs. no additional coaching. The staff member will mail a flyer to parents who express interest in the extended follow-up. Parents will be encouraged to discuss the extended follow-up option with their teens prior to the 18-month measurement visit.

At the end of the 18-month visit, parents/teens who expressed interest in the extended follow-up will be given an opportunity to review the study requirements with a staff member and to ask any questions they might have. If they choose to participate, the staff member will obtain written consent from parents and participants >18 yrs. of age, and assent from teens who are <18 yrs. of age.

11c. Randomization Current control participants will remain in the control group. Only current intervention participants will be randomized. Randomization will be managed within the study website, with oversight from UNC, Chapel Hill. Participants will be randomized at the level of the individual, stratified by site, then by HbA1c categories (8.0-9.0% and 9.1-13.0%) using a block size of 4 within each of the strata. Current Intervention participants will be assigned to continued intervention/coaching or control (no continued intervention/coaching) according to a preset schedule based on date of enrollment in a 1:1 ratio. Upon completion of the 18-month standardized measurement visit, the randomization assignment will be opened by the research staff and the participant will be informed as to whether they have been assigned to the FL3X continued intervention/coaching group or control group after they have completed all measurements including wearing the CGM.

11d. Standardized Measures Participants from all three groups will be seen for a measurement visit at 24 months; and those who are eligible for two follow-up measurement visits will also be seen at 30 months post-baseline visit. Standardized measures at these visits will be identical to the standardized measures administered at the 12-month visits with the addition of the physical exam. See tables below.

Participants will receive an incentive of $50 for each of the measurement visits; and parents will receive an incentive of $20 incentive for each visit.

Table 11.1 Overview of Standardized Measures for Parents
(forms self-administered unless otherwise noted)

**Parent**		
	**24 mos**	**30 mos**
**Contact Information**	X	X
**Peds QL – Generic Quality of Life**	X	X
**Diabetes Self-Management Assessment (DSMP-SR)**	X	X
**Diabetes Family Conflict Scale**	X	X
**Diabetes Family Responsibility**	X	X
**Diabetes-Specific Quality of Life (PDQ)**	X	X
**Specific Health Care Use** *(Interviewer Administered)*	X	X

Table 11.2 Overview of Standardized Measures for Participants
(forms self-administered unless otherwise noted)

**Participant**		
	**24 mos**	**30 mos**
**Contact Information**	X	X
**Specimen Collection Form**	X	X
**HbA1c**	X	X
**Physical Exam**	X	X
**Intention and Motivation**	X	X

**Social Problem-Solving Inventory-Revised : Short** *(In person only)*	X	X
**Peds QL – Generic Quality of Life**	X	X
**Diabetes Self-Management Assessment (DSMP-SR)**	X	X
**Diabetes Family Conflict Scale**	X	X
**Diabetes Family Responsibility**	X	X
**Diabetes-Specific Quality of Life (PDQ)**	X	X
**Outcome Expectations for Diabetes Self-Management**	X	X
**Self-Efficacy for Diabetes Self-Management**	X	X
**Depression symptoms (CES-D)** *(In person only)*	X	X

11e. Extended Intervention Participants who are randomized to continued intervention will use the same adaptive design as described in Section 9b. They will be assigned to Regular coaching or Check-in coaching based on A1c measurements at the 18-month visit. Participants who are eligible for two follow-up measurement visits will also be re-assigned to Regular coaching or Check-in coaching based on A1c measurements at the 24-month visit. Regular and Check-in coaching sessions will be scheduled every 3 months during the period of extended follow-up until the final follow-up measurement visit has been completed.

12. Data management

The project will utilize the Sheps Integrated Research System (SIRS), a secure, enterprise database and programming framework specifically designed to meet the needs of health related research projects at the Cecil G. Sheps Center at UNC Chapel Hill. This web-based system will be used to track study participants, prompt study staff for participant follow-up, and enter study data collection forms. All system login procedures and data submissions will be transported encrypted via the Secure Sockets Layer (SSL) protocol to a secure central database at the Sheps Center. User-level permissions will be based on user roles and defined within the project system to limit a user's access to only those records an individual is authorized to see. Sheps Center programmers and research staff who work with sensitive data are required to complete appropriate HIPAA training with periodic updates, complete Sheps Center internal training, comply with the UNC IT Security Policies, and agree to the provisions of the Sheps Center's Rules of Behavior and Sanction Policy. The Sheps Center strives to implement reasonable security controls guided by FISMA, HIPAA, and OMB Circular A-130, Appendix III.

Upon entry into the study, a unique identifying number will be assigned to each participant. This number will be used to identify the information and specimens collected and stored during this study. Specimens collected and transferred to the central laboratory will have no identifier other than the unique identifying number assigned to the participant. The roster containing the unique identifying number and direct participant identifiers will be kept in a local password-protected database on a secure network drive at the clinical sites.

13. STATISTICAL CONSIDERATIONS

Sample Size and Power Our power analysis is based on detecting a clinically desirable effect size (ES) for our primary outcome of HbA1c. We also considered secondary outcomes, potential mediation and effect modification. Use of an adaptive intervention has no impact on the approach to sample size estimation or analysis [135]. The Type 1 error rate for each test is 0.05. We determined a final sample size of 200 would be optimal, considering the totality of our power analysis described below.

Primary outcome: Although the statistical analysis will utilize all data collected at multiple time points as described below, we took the conservative approach to employ a simple two-sample t-test to estimate the detectable intervention effect on HbA1c at the 24 month time point. Table 12.a. shows that a **total n of 200 will allow detection of a clinically meaningful intervention effect on HbA1c of 0.60%; this translates to an ES of 0.40.** This is a considerably smaller ES than that observed by Wysocki et al [136] (0.53) and smaller than that observed in our "high fidelity" group (0.57). Using results from our Pilot study, we also considered power to detect the proportion of intervention versus control participants who experience a decline in HbA1c of ≥0.5%. For a sample size of 200, we can detect an ES of 0.41, which compares to the actual result of the FL3X Pilot study of ES=0.37 for the study overall, and ES=0.65 for the "high fidelity" group. **Thus a sample size of 200 total participants at the end of the FL3X study will allow detection of a clinically meaningful change in HbA1c with appropriate statistical power.**

Table 12.a. Estimated change in HbA1c (and corresponding effect size) detectable with 80%, 85%, and 90% power at N=100, 150, and 200.

Total N completing the two-year follow-up (half in control and half in intervention)	Detectable intervention effect on HbA1c (effect size)		
	80% power	85% power	90% power
100	0.71% (0.47)	0.74% (0.49)	0.80% **(0.53)**
150	0.57% (0.38)	0.60% (0.4)	0.65% (0.43)
200	0.50% (**0.33**)	0.53% (0.35)	0.56% (0.37)

Table 12.b.

	Power for total N=200					
% HbA1c decline of ≥ 0.5	73%	99%	80%	85%	90%	95%
Intervention	41	54	43	44	46	49
Control	24	24	24	24	24	24

41%: observed in Pilot Intervention group (all)
54%: observed in Pilot Intervention (fidelity > median)
24%: observed in Pilot Control group (similar to SEARCH – 28% had HbA1c decline ≥ 0.5 between 12 and 24 mo follow up visit)

Secondary Outcomes Again, using the conservative two-sample t-test approach, Table 12.c shows the detectable intervention effects for secondary outcomes for a total N of 200, showing reasonable ability to detect clinically

**Table 12.c.** Detectable differences for FL3X secondary outcomes, for a total n=200 (100 intervention, 100 control); conservative effect size, 0.40	**Least detectable difference corresponding to effect size**
Motivation	0.65
Problem Solving Skill	0.39
Quality of Life Child (PedQL)	5.42
BMI	1.7 kg/m^2
SBP	4.3
LDL-c	11.2 mg/dl
HDL-c	5.1 mg/dl
Triglycerides (log-transformed)	0.21 log-mg/dl

meaningful effects. We note that in the In Vivo trial by Seid et al [137], the ES for motivation using the same measurement instrument was 0.67 and observed change in the motivation score was 1.08, confirming that the FL3X RCT is appropriately powered given the underlying framework for behavior change.

Mediation Using Baron and Kenny's [138] causal steps, mediation analysis involves confirming the effect of the intervention on the potential mediator and the effect of the mediator on the outcome. The effect of the mediator on the outcome must remain significant after controlling for intervention status. The effect of the intervention on the outcome must be reduced (partial mediation) or disappear (total mediation) when the mediator is added to the model. Fritz and MacKinnon [139] performed simulations to estimate the required sample size for Baron and Kenny's method. These numbers are summarized in Table 12.d. for various effect sizes in a partial mediation setting. Data from SEARCH indicate that the effect of the mediators on HbA1c is likely to be in the 0.20-0.40 range (for example, the estimated ES for checking blood glucose at least 4x/day was 0.41). A total n of 200 provides reasonable opportunity to detect clinically relevant mediation.

Table 12.d.

**Effect of intervention on mediator**	**Effect of mediator on outcome**		
	**.26**	**.39**	**.59**
**.26**	224	179	153
**.39**	178	118	88
.59	147	84	53

Effect Modification: Cakan et al. [140] suggested that the multi-systemic intervention for youth with T1D had differential results based on weight status. In order to achieve adequate power to detect moderate effect modification, we would need to recruit more than 500 participants, which is a prohibitive number to add beyond what we need for the primary outcome. The sample size of 200 gives moderate power to detect very strong effect modification, where there is nearly no effect in one subgroup and a very large effect in another (Table 12.e.). As we don't expect such extreme effect modification, we choose instead to examine effect modifiers in an exploratory way, estimating treatment effects in subgroups to inform future studies.

Table 12.e. Power for modification of effect by sociodemographic risk: N=150, 200, or 250

Total N completing the two-year follow-up	Power to detect effect modification by socio-demographic risk, assuming the sample is about 50% high risk		Power to detect effect modification by weight status, assuming the sample is about 33% overweight or obese	
	The size of the intervention effect is .6 in one group and .2 in the other	The size of the intervention effect is .7 in one group and .1 in the other	The size of the intervention effect is .6 in one group and .2 in the other	The size of the intervention effect is .7 in one group and .1 in the other
150	29%	60%	26%	55%
200	38%	74%	33%	68%
250	47%	84%	42%	79%

Randomization. Participants will be assigned at random to the intervention and control groups in a 1:1 ratio, stratified by site and HbA1c at enrollment and using a block size of 4. Randomization will be managed via the UNC-based data management system and will not be revealed to staff or participants until completion of baseline measurements.

Statistical Analysis We note that the statistical analysis approach for evaluation of an RCT using an adaptive intervention is unchanged from the usual intent-to-treat analysis [141]. Briefly, to test the effect of the FL3X intervention on *primary and secondary outcomes*, we will use a mixed effects model [142], incorporating all available data. Fixed effects will include timepoint (categorical), intervention group, indicator x timepoint, and controlling for the baseline level of the outcome. A random intercept will be included to account for within-participant correlation. The estimates and tests of the coefficients of the interaction term will provide

evidence for the effectiveness of the intervention at each timepoint, thus allowing careful evaluation of the timing and sustainability of intervention effects. The process of randomization will be evaluated and variables that may confound analysis of change over time found, by chance, not to be randomly distributed, will be included in the models. Site will be included in the models, and potential site differences in intervention effect will be explored using the appropriate interaction terms. *Effect modification* related to socio-demographic and weight status will be explored using the relevant interaction terms. *Mediation* will be evaluated using the method described above. Further, in post-hoc analyses of the intervention group, we will explore time spent in FL3X-Check-in versus FL3X-Regular as a potential mediator.

Missing Data

The ITT (intent to treat) approach has been designed following EMEA's guidelines for missing data for confirmatory clinical trials. We expect the main efficacy analysis to include all available data from participants on an ITT basis, assuming the rest of the data are MCAR.

However, the effect of missing data will be assessed through analyses of dropout and possible sensitivity analyses. The details will depend heavily on the amount and type of missing data, but the general principles follow here.

We will initially take steps to examine the patterns of the missing data. Though there is no way to know for sure whether data are MAR or non-ignorably missing, we will examine missing data as it relates to:

Internal validity: Dropout in control vs. intervention arms- overall rate and characteristics of those who dropped out. This will help us assess the risk of threats to internal validity, the case when imputation would be most necessary. If there is evidence of differential missingness, missing data will be imputed as a sensitivity analysis- the imputation method will depend on the amount and nature of the missing data.

External validity: If missingness is related (in both treatment groups) to certain participant characteristics, we will assess the results of the efficacy analysis in light of these underrepresented individuals, perhaps through subgroup analysis or upweighting of remaining participants like those who are missing.

14. Potential Risks, Discomforts, Inconveniences, & Precautions:

Physical risks to the participants are minimal and limited to risks associated with fasting and collection of blood via venipuncture. Drawing blood from a vein in the lower arm may cause mild pain and, occasionally, minor bruising at the site of the blood draw. Fainting can also occur. Because the study subjects have diabetes, the requirement of a fasting blood sample may precipitate a temporary blood glucose level that is somewhat lower or a higher than usual. Psychological and social risks are expected to be minimal (e.g., potential embarrassment about answering questions related to adherence). One risk of the current project is breach of confidentiality. This is highly unlikely as data are used exclusively for research purposes, and youth will be provided with information on how risks to confidentiality are minimized, including use of de-identified study documents and locked, and/or password-protected files.

There may be an inconvenience for parents and children because of extra visits with research.

Technology use is encouraged and different modalities of web communication may be used throughout this study to support participant efforts to adhere to the diabetes care plan. However, it is unrealistic and unfeasible to monitor all study participant activities on the web and the security of all website cannot be verified by study staff. Staff will not ask participants to disclose personal information over websites that are not secure. To minimize the potential risk to participants, a handout will be provided to all intervention participants highlighting safe internet practices (see Appendix of the Intervention MOP).

There may be unknown or unforeseen risks associated with study participation.

Vulnerable Population: Children and adolescents will be included in this study. Special consideration for their vulnerable status will be made by ensuring their written assent to participate, by keeping their information confidential, and by reminding them of the voluntariness of the study and their right to withdraw their assent without penalty at any time.

Protections against Risk

All institutions associated with this study, including UNC at Chapel Hill, the two clinical sites, and the Central Laboratory are experienced with handling sensitive and confidential data. Certain routine administrative, personnel, physical security, information management, and computer system or network security practices are always in place given the policies and the requirements for safeguards consistent with the management of PHI at both clinical sites. All PHI will be used or disclosed in compliance with the Health Insurance Portability and Accountability Act (HIPAA). Forms will be kept in locked files when not in use. Subjects will be assigned identification numbers in lieu of names on data management and data analysis files. Participant name and telephone contact will be shared with UNC The roster containing the unique identifying number and direct participant identifiers will be kept in a local password-protected database on a secure network drive at the clinical sites. Upon completion of the study, these files will be destroyed. No individual PHI will be used in reports or manuscripts. Data entered and stored on the microcomputer will be archived daily. The data collectors will follow a strict written protocol that describes study measures for protecting data privacy. Patients and parents always have the right to refuse to participate or to refuse to answer any individual question they might find objectionable. Adolescents and parents will be informed, as part of the informed consent process, regarding the limitations of confidentiality.

To minimize the possibility of risks associated with the blood draw, experienced medical staff will obtain the blood samples. A local numbing medicine may be placed on the skin before the blood is drawn to decrease any pain. Subjects who have a history of fainting or who develop symptoms of light-headedness will be placed in the supine position.

The minimum amount of blood/plasma necessary to conduct these tests will be collected and the total amount collected will not exceed standard, weight-specific guidelines. Blood volumes will range from 2 ml to 9 ml. per visit. Stored plasma will be saved for 5 years after the end of the study. The following table describes the amount of blood required for the laboratory tests.

MEASUREMENT	Blood Volume
**Hemoglobin A1c**	2 ml
**Lipid profile**	3 ml
**Stored plasma**	4 ml
**Total maximum blood volume**	9 ml

Study personnel will be trained to identify the signs and symptoms of a blood glucose level that is low or high. They will also be trained to check the blood glucose level, using a glucometer. If low blood glucose occurs during a study visit (< 70 mg/dL), study personnel will be trained to administer 15 grams of an oral carbohydrate, and to repeat as needed every 10 minutes until the blood glucose level is > 70 mg/dL. If the blood glucose level is above 300 mg/dL during a study visit, study personnel will be trained to check for blood or urinary ketones. After fasting blood specimens have been obtained and the blood glucose level has been measured, subjects will be instructed to take their usual dose of insulin or other diabetes medication as prescribed; and the subject will then be given breakfast. In cases of low or high glucose levels (with or without the presence of ketones), additional medical interventions may sometimes be needed. Local policies dictate these procedures, which may include a one-time adjustment in the dose of insulin taken and/or the administration of glucose gel, glucagon, or intravenous glucose. If participants experience hypoglycemia or hyperglycemia outside of study visits, they will be advised to treat these situations per their usual diabetes care plan.

Individual participant blood pressures will be compared to a table of blood pressure levels at the 95th percentile (per the NHLBI guidelines table), based on the participant's gender, age, and height percentile. If the participant's blood pressure (systolic and/or diastolic) is greater than the 95th percentile, the participant or the parent (if the participant is <18 years of age) will be informed that the participant's blood pressure is higher than expected. If the participant is not already being monitored and treated for high blood pressure, study personnel will recommend that they follow-up with their healthcare provider. If the blood pressure is greater than the 99th percentile plus 5, study staff will assist the participant in obtaining immediate medical attention as needed based on local guidelines and procedures.

Participants or their parents (if the participant is <18 years of age) will receive the results for study HbA1c and fasting lipid measurements. Their providers will also receive these results, if the parent/participant agrees to this during the informed consent process. Participants and their parents will be encouraged to discuss their lab results with their providers. If a participant's triglycerides ever exceed 1000 mg/dL, the Central Lab will notify the local PI or his designee, who will then follow-up per local guidelines and procedures.

Participants will be completing a CES-D form at all the measurement visits. Although the CES-D is not considered a diagnostic tool for the identification of clinical depression, it is designed to identify people who may be suffering from a depressed mood. Whenever a participant scores high (> 24) on the CES-D, study personnel will inform the participant or the parent (if the participant is <18 years of age) that the CES-D score is "high" compared to what would be expected for most adolescents, indicating that the participant may be clinically depressed. If the participant is currently receiving mental health counseling or treatment, study personnel will recommend follow-up with his/her mental health provider. If the participant is not being treated by a mental health provider, study personnel will make a referral, based on local guidelines and procedures.

Suicidal ideation will be assessed at each measurement visit. If the subject indicates that he/she thinks about killing him/herself or wants to kill him/herself, each site will follow-up based on local clinic standards. Specific follow-up for each site will include:

Ohio

If a subject responds he/she is thinking about killing him/herself, assessment staff will contact the Psychiatric Intake Referral Center (PIRC), a 24/7 hotline run by CCHMC Division of Psychiatry and staffed with trained suicide assessment personnel with backup from licensed pediatric psychiatrists. The family will receive appropriate referral (including to CCHMC ER), depending on the PIRC recommendations. If a subject responds he/she wants to kill him/herself, they will be referred to the CCHMC ER.

Colorado

If a subject responds he/she is thinking about killing him/herself and a clinic social worker is available, the clinical social worker would make an assessment and decide on the appropriate referral. If the clinic social worker is not available or the subject responds he/she wants to kill him/herself, assessment staff will call the CHC ED and send the child and parent over directly to be evaluated.

At both sites, assessment staff will be trained how to communicate with the family the need for further assessment or referral and assure the proper referral is made and a caregiver is involved. Assessment staff will immediately notifies the study doctor or designee. The study doctor or designee will follow up within 24 hours to assess the status of the patient and referral.

<u>Spontaneous reporting of suicidality during interventions</u>

If a subject spontaneously reports suicidality during an in-person intervention session, the coach will respond by asking the subject to clarify, using Item 9 from the CDI (administered verbally). The response of thinking about killing him/herself or wanting to kill him/herself will be as for assessment staff above.

If the intervention session is not in person (i.e. by phone or Skype), the coach will attempt to contact the parent while the subject is still on the phone and will recommend to the parent that they take the subject to the nearest ER. If the coach is unable to contact the parent, they will leave a message and contact the local police to be dispatched to the subject's location.

At both sites, intervention staff will be trained how to communicate with the family the need for further assessment or referral and assure the proper referral is made and a caregiver is involved. Intervention staff will immediately notifies the study doctor or designee. The study doctor or designee will follow up within 24 hours to assess the status of the patient and referral.

15. Risk/Benefit Analysis

Participation in this research study may help to improve glycemic control in some participants.

Potential risks for the participants are minimal and associated with possible discomfort with blood draw, responding to surveys and loss of confidentiality. We have instituted provisions to minimize risk by assuring participants of the voluntariness of their participation and their right to withdraw participation at any time, and by taking appropriate steps to safeguard confidentiality. We would suggest that this study falls under the “Minimal Risk” category.

16. DATA SAFETY & MONITORING:

The NIDDK will convene a 5-member Data and Safety Monitoring Board to meet twice yearly during the trial. Drs. Mayer-Davis, Seid, and Maahs, along with Ms Joan Thomas, will represent the FL3X team in interactions with the DSMB. The DSMB will review the final study protocol and any significant changes to the protocol over the course of the study especially as related to participant burden or safety. The DSMB will monitor and advise on study participant accrual and retention, progress and completeness for all standardized measurement visits and attendance at intervention and control sessions. Data quality will be reviewed and staff training and certification will be monitored. Adverse events will be monitored. A summary of the DSMB report will be sent to both the local site IRBs and NIDDK as part of the annual progress reports.

Data Monitoring

There are three types of data to be monitored:

1. Recruitment and retention
2. Data from the 5 standardized data collection time-points: Questionnaires, administered to both participants and caregivers (usually a parent); physical examination data (anthropometry, blood pressure); and laboratory data (HbA1c, lipid profile)
3. Intervention fidelity measures based on audio-tapes of actual intervention sessions.

Internally, all aspects of data collection and data storage will be carefully monitored to ensure rapid detection of errors, inconsistencies or other problems. The data collectors will follow a strict written protocol that describes study measures for protecting data privacy. They will explain to each participant that s/he has the right to refuse to participate or to refuse to answer any individual question that s/he finds objectionable, and emphasize the importance of telling the truth. All institutions associated with this application are experienced in training data collection fieldwork personnel how to handle, store, and process sensitive and confidential data. Certain routine administrative, personnel, physical security, information management, and computer system or network security practices are always in place. These practices include building and audio-tape vault security, non-disclosure pledges, and account/keyword security on computer networks. In addition, we take multiple project-specific steps to protect subjects from the risk of a breach in confidentiality. All data will be collected using study identification numbers. Thus, no questionnaire will contain identifying information, and the list that links identification numbers to names will be kept in a password-protected file that is accessible only to authorized staff at the respective clinical sites. Only aggregate data that cannot be used to identify individuals will be included in any reports released to other agencies or for publication.

Twice yearly, we will provide the DSMB with data to review; attached are table shells for review and discussion. We will be happy to modify the tables to accommodate DSMB requests.

Population

The intervention will involve youth with type 1 diabetes who are 13-16 years of age who are patients at either the Barbara Davis Center for Childhood Diabetes (Denver, CO) or Cincinnati Children's Hospital and Medical Center pediatric endocrinology clinic, with diabetes duration of at least one year and most recent HbA1c of 8-13% based on medical records from the previous year.

Adverse Events

For purposes of monitoring and reporting adverse events, the following NIH definitions will be used:

Adverse Event (AE): any unanticipated, untoward medical occurrence that may present itself during treatment or administration of an intervention, and which may or may not have a causal relationship with the treatment. Adverse events could arise from the study (e.g., breach of confidentiality) or could arise due to the population under study.

Serious Adverse Event (SAE): Any medical occurrence that results in death; is life-threatening; requires inpatient hospitalization or prolongation of existing hospitalizations; creates persistent or significant disability/incapacity, or a congenital anomaly/birth defect.

It is unlikely that participation in our intervention will cause youth to experience an "adverse event," however; trained staff will be conducting the intervention sessions to address any potential issues. Any youth experiencing hypo/hyperglycemia or an otherwise potentially expected symptom related to diabetes will be encouraged to consult their diabetes care provider or to go to the emergency room immediately as needed.

Should an AE occur, however, the coach will immediately report any AE's connected to the implementation of the intervention to the study investigators, who will keep a log of AE's and SAE's. The log will also be used to provide information about AE's in annual progress reports to participating IRBs and NIDDK.

Any AEs will be reviewed by the study Executive Committee to determine if an AE is related to the research project. Our team will file a written report to the site IRBs in accordance with local IRB requirements, and to NIDDK. Outcomes for AE's will be monitored by the DSMB and outcome information will be entered into a log for inclusion in reports to participating IRBs and NIDDK as required.

Anticipated events relative to the population under study (e.g., low or high blood glucose) or relative to study activities (e.g., fainting associated with venipuncture) will be noted as potential risks on the informed consent form. High blood glucose accompanied by DKA needing emergency response, low blood sugar requiring assistance for treatment (requiring glucagon, or causing seizure or loss of consciousness), or hospitalizations (for any reason) will be included as a reportable event.

Should any other problems or concerns arise with the study recruitment or retention, data collection or intervention program, the PI or local clinical PI will be available to address these. In addition, resource and referral listings for community services will also be provided on a routine basis as needed. Drs. Mayer-Davis, Seid, Dolan, and Maahs will also work extensively with the staff at each of the clinics to ensure that if adolescents need to be referred that these situations are managed in a manner consistent with clinic preferences/policies.

Study Stopping Rules and Related Issues

The study has not proposed early stopping rules or associated interim analyses, thus our sample size and power calculations do not account for loss of power associated with interim analyses. Reasons for this are noted below.

Recruitment and retention: FL3X has developed a very rigorous plan for recruitment and retention, including a two-stage recruitment process that engages the potential participant using motivational interviewing strategies to ensure that the agreement to participate is made thoughtfully. Incentives are as generous as possible and many strategies are in place to facilitate ongoing participation (including capacity for online entry of survey data by participants to minimize time in clinic, and for intervention participants, capacity for Skype for some sessions to lesson transportation burden). We will monitor recruitment and retention weekly at each site, with periodic discussions on the weekly Executive call to share ideas, plus review and discussion monthly on study-group calls. The DSMB will monitor recruitment and retention regularly to ensure that the study is progressing appropriately and that the study will be adequately powered to accomplish the study aims.

Interim analyses and stopping rules:
Reasons for un-blinded interim analyses are to evaluate results relative to established stopping rules to address safety, benefit, or futility. Interim analyses impact on statistical power and so require adjustment to sample size estimations. The FL3X team has not established stopping rules, and therefore we have not planned on interim analyses for the following reasons:

1. *Safety*. As a behavioral intervention aimed to improve diabetes self-management, we do not expect any issues with safety related to the intervention. The DSMB will regularly monitor adverse events.

2. *Benefit*. As indicated in the grant, we are interested in two subgroups, namely the subgroup of those at socio-demographic “high risk” (namely, those with low income, minority race/ethnicity, or single parent household) versus all others, and those overweight or obese versus those of normal or underweight. Therefore we would not want to stop the study early due to benefit in the whole sample because that would limit capacity to consider potential differential effects within these subgroups. Further, we have a number of secondary outcomes, and potential mediators – an early stop could substantially limit capacity to conduct those analyses.

3. *Futility*. Here, the issue is to ensure that money is not being spent on an intervention with no hope of benefit. For example, if we looked at the data and determined to stop early for futility after 50% of the sample had completed the intervention, we would save about 10 months’ worth of study expenses (for measurements and for intervention delivery). Given that is not a tremendously long time (compared to multi-year interventions rather than an 18 month intervention), and would substantially limit capacity to consider potential benefit on secondary outcomes (e.g., quality of life), we would not wish to stop early for futility. Importantly, this study is not considered to be unduly burdensome to participants, relative to the usual care of patients with type 1 diabetes and the day-to-day demands of diabetes self-management.

16. Privacy & Confidentiality

To protect the right of the participant's privacy, the patients' disease status will not be included on the mailing envelope of any recruitment materials for the study. In-person recruitment will occur in the diabetes clinics by study personnel. Study visits will occur in private settings.

To minimize the risk of loss of confidentiality, all information related to study subjects will be confidential and kept in secure cabinets or password-protected computer files, in compliance with NIH and HIPAA requirements as detailed in NIH Notice OD-020 issued December 30, 2004.

Each participant will be assigned a unique study identification (ID) number. Participants will be identified on all study-related documents only by the study ID numbers. No personally identifiable information (e.g. name, birth date, address, phone number, medical record number, insurance number, social security number) will be used on the data collection forms. The roster containing the unique identifying number and direct participant identifiers will be kept in a local password-protected database on a secure network drive at the clinical sites.

All hard copy data collection forms will be secured in a locked file cabinet or office. Data will be entered onto a secure Web-based data management system. Only the study coordinator and relevant research study staff will have access to this study database. Secure access will be assured by use of individual login codes and password protection. Entered data will be stored securely and accessed in accordance with current HIPAA standards, the HCFA's Internet Security Policy, and other state and local requirements. Data entered onto the database will be identified only by the study ID numbers and the participants' first names and last initials.

All data interactions for the study by all users – including participants, coaches, clinical staff, research staff, and investigators – are web-based communications between the given user's web browser and the web server running the study website and/or data management software. All of these web-based communications are configured to use the standard HTTPS protocol with 128-bit encryption. This includes online forms submitted by participants as they are transferred to the servers at UNC.

The project will utilize the Sheps Integrated Research System (SIRS), a secure, enterprise database and programming framework specifically designed to meet the needs of health-related research projects at the Cecil G. Sheps Center at UNC Chapel Hill. This web-based system will be used to track study participants, prompt study staff for participant follow-up, present data collection forms and allow remote sites to key data directly into the central database. All system login procedures and data submissions will be transported encrypted via the Secure Sockets Layer (SSL) protocol to a secure central database at the Sheps Center. User-level permissions will be based on user roles and defined within the project system to limit a user's access to only those records an individual is authorized to see.

This study will utilize a HIPAA compliant external commercial vendor for transcription services (MT-Stat; www.mt-stat.com). The digitally recorded interview is sent directly to the vendor over the internet and a document containing the transcription is returned to the study staff over the internet by the vendor.

The intervention does not require transmission of PHI by any cell phone. We recognize that participants or parents may take the initiative to send PHI by cell phone. As is the case with all

behavior intervention utilizing current technology, we will explain to participants and parents that transmission of such information is not secure.

Data will be obtained and stored based on structured interviewing of intervention acceptability, and audio-tapes of session delivery. All aspects of data collection and data storage will be carefully monitored to ensure rapid detection of errors, inconsistencies or other problems. The data collectors will follow a strict written protocol that describes study measures for protecting data privacy, explain to each participant that s/he has the right to refuse to participate or to refuse to answer any individual question that s/he finds objectionable, and emphasize the importance of telling the truth. All institutions associated with this study are experienced in training data collection fieldwork personnel how to handle, store, and process sensitive and confidential data. Certain routine administrative, personnel, physical security, information management, and computer system or network security practices are always in place. These practices include building and audio-tape vault security, non-disclosure pledges, and account/keyword security on computer networks.

In addition, we take multiple project-specific steps to protect subjects from the risk of a breach in confidentiality. All data will be collected using study ID numbers. Thus, no questionnaire will contain identifying information, and the roster containing the unique identifying number and direct participant identifiers will be kept in a local password-protected database on a secure network drive at the clinical sites. Finally, only aggregate data that cannot be used to identify individuals will be included in any reports released to other agencies or for publication.

17. Study Timeline

	COMPLETED PRIOR TO 09/01/2013	Year 1 09/01/2013-08/31/2014				Year 2 09/01/2014-08/31/2015				Year 3 09/01/2015-08/31/2016				Year 4 09/01/2016-08/31/2017				Year 5 09/01/2017-6/30/2018			
		Sep-Nov	Dec-Feb	Mar-May	June-Aug	Sep-Nov	Dec-Feb	Mar-May	June-Aug	Sep-Nov	Dec-Feb	Mar-May	June-Aug	Sep-Nov	Dec-Feb	Mar-May	June-Aug	Sep-Nov	Dec-Feb	Mar-May	June
ADMINISTRATIVE ACTIVITIES																					
IRB review																					
IRB approval and renewals																					
Hiring staff																					
Training staff																					
Finalizing detailed MOPs																					
DATA COLLECTION																					
Recruitment																					
Standardized measurements visits																					
INTERVENTION																					
FL3X-Basic																					
FL3X-Check-in																					
FL3X-Regular																					
DATA MANAGEMENT AND QUALITY CONTROL																					
Data quality monitoring																					
Data analysis																					
PRESENTATIONS AND PUBLICATIONS																					
Baseline Cohort paper																					
Abstract(s) to ADA Sci Sessions																					
Publications and presentations																					

Reference List

(1) Tamborlane WV, Beck RW, Bode BW et al. Continuous glucose monitoring and intensive treatment of type 1 diabetes. *N Engl J Med* 2008 October 2;359(14):1464-76.

(2) Wang YC, Stewart SM, Mackenzie M, Nakonezny PA, Edwards D, White PC. A randomized controlled trial comparing motivational interviewing in education to structured diabetes education in teens with type 1 diabetes. *Diabetes Care* 2010 August;33(8):1741-3.

(3) Wysocki T, Harris MA, Buckloh LM et al. Randomized trial of behavioral family systems therapy for diabetes: maintenance of effects on diabetes outcomes in adolescents. *Diabetes Care* 2007 March;30(3):555-60.

(4) Channon SJ, Huws-Thomas MV, Rollnick S et al. A multicenter randomized controlled trial of motivational interviewing in teenagers with diabetes. *Diabetes Care* 2007 June;30(6):1390-5.

(5) Channon SJ, Huws-Thomas MV, Rollnick S et al. A multicenter randomized controlled trial of motivational interviewing in teenagers with diabetes. *Diabetes Care* 2007 June;30(6):1390-5.

(6) Carroll AE, Marrero DG, Downs SM. The HealthPia GlucoPack Diabetes phone: a usability study. *Diabetes Technol Ther* 2007 April;9(2):158-64.

(7) Franklin VL, Greene A, Waller A, Greene SA, Pagliari C. Patients' engagement with "Sweet Talk" - a text messaging support system for young people with diabetes. *J Med Internet Res* 2008;10(2):e20.

(8) Wysocki T, Harris MA, Buckloh LM et al. Randomized, controlled trial of Behavioral Family Systems Therapy for Diabetes: maintenance and generalization of effects on parent-adolescent communication. *Behav Ther* 2008 March;39(1):33-46.

(9) The effect of intensive treatment of diabetes on the development and progression of long-term complications in insulin-dependent diabetes mellitus. The Diabetes Control and Complications Trial Research Group. *N Engl J Med* 1993 September 30;329(14):977-86.

(10) Dorman JS, LaPorte RE, Kuller L et al. The Pittsburgh insulin-dependent diabetes mellitus (IDDM) morbidity and mortality study. Mortality results. *Diabetes* 1984;33(3):271-6.

(11) Krolewski AS, Kosinski EJ, Warram JH et al. Magnitude and determinants of coronary artery disease in juvenile-onset, insulin-dependent diabetes mellitus. *Am J Cardiol* 1987;59(8):750-5.

(12) Petitti DB, Klingensmith GJ, Bell RA et al. Glycemic control in youth with diabetes: the SEARCH for diabetes in Youth Study. *J Pediatr* 2009 November;155(5):668-72.

(13) Petitti DB, Klingensmith GJ, Bell RA et al. Glycemic control in youth with diabetes: the SEARCH for diabetes in Youth Study. *J Pediatr* 2009 November;155(5):668-72.

(14) Rodriguez BL, Fujimoto W, Mayer-Davis E et al. Prevalence of Cardiovascular Disease Risk Factors in U.S. Children and Adolescents with Diabetes: The SEARCH for Diabetes in Youth Study. *Diabetes Care* 2006;29(8):1891-6.

(15) Paris CA, Imperatore G, Klingensmith G et al. Predictors of Insulin Regimens and Impact on Outcomes in Youth with Type 1 Diabetes: The SEARCH for Diabetes in Youth Study. *Journal of Pediatrics* 2009.

(16) Naughton MJ, Ruggiero AM, Lawrence JM et al. Health-related quality of life of children and adolescents with type 1 or type 2 diabetes mellitus: SEARCH for Diabetes in Youth Study. *Arch Pediatr Adolesc Med* 2008 July;162(7):649-57.

(17) Lawrence JM, Standiford DA, Loots B et al. Prevalence and correlates of depressed mood among youth with diabetes: the SEARCH for Diabetes in Youth study. *Pediatrics* 2006 April;117(4):1348-58.

(18) Steck AK, Klingensmith GJ, Fiallo-Scharer R. Recent advances in insulin treatment of children. *Pediatr Diabetes* 2007 October;8 Suppl 6:49-56.

(19) Hirsch IB. Insulin analogues. *N Engl J Med* 2005 January 13;352(2):174-83.

(20) Levine BS, Anderson BJ, Butler DA, Antisdel JE, Brackett J, Laffel LM. Predictors of glycemic control and short-term adverse outcomes in youth with type 1 diabetes. *J Pediatr* 2001 August;139(2):197-203.

(21) Moreland EC, Volkening LK, Lawlor MT, Chalmers KA, Anderson BJ, Laffel LM. Use of a blood glucose monitoring manual to enhance monitoring adherence in adults with diabetes: a randomized controlled trial. *Arch Intern Med* 2006 March 27;166(6):689-95.

(22) Mayer-Davis EJ, Nichols M, Liese AD et al. Dietary intake among youth with diabetes: the SEARCH for Diabetes in Youth Study. *J Am Diet Assoc* 2006 May;106(5):689-97.

(23) Herbst A, Kordonouri O, Schwab KO, Schmidt F, Holl RW. Impact of physical activity on cardiovascular risk factors in children with type 1 diabetes: a multicenter study of 23,251 patients. *Diabetes Care* 2007 August;30(8):2098-100.

(24) Lobelo F, Liese AD, Liu J et al. Physical activity and electronic media use in the SEARCH for diabetes in youth case-control study. *Pediatrics* 2010 June;125(6):e1364-e1371.

(25) Helgeson VS, Viccaro L, Becker D, Escobar O, Siminerio L. Diet of adolescents with and without diabetes: Trading candy for potato chips? *Diabetes Care* 2006 May;29(5):982-7.

(26) Overby NC, Margeirsdottir HD, Brunborg C, Anderssen SA, Andersen LF, hl-Jorgensen K. Physical activity and overweight in children and adolescents using intensified insulin treatment. *Pediatr Diabetes* 2008 August 20.

(27) Tamborlane WV, Beck RW, Bode BW et al. Continuous glucose monitoring and intensive treatment of type 1 diabetes. *N Engl J Med* 2008 October 2;359(14):1464-76.

(28) Wang YC, Stewart SM, Mackenzie M, Nakonezny PA, Edwards D, White PC. A randomized controlled trial comparing motivational interviewing in education to structured diabetes education in teens with type 1 diabetes. *Diabetes Care* 2010 August;33(8):1741-3.

(29) Wysocki T, Harris MA, Buckloh LM et al. Randomized, controlled trial of Behavioral Family Systems Therapy for Diabetes: maintenance and generalization of effects on parent-adolescent communication. *Behav Ther* 2008 March;39(1):33-46.

(30) Wysocki T, Harris MA, Buckloh LM et al. Randomized trial of behavioral family systems therapy for diabetes: maintenance of effects on diabetes outcomes in adolescents. *Diabetes Care* 2007 March;30(3):555-60.

(31) Channon SJ, Huws-Thomas MV, Rollnick S et al. A multicenter randomized controlled trial of motivational interviewing in teenagers with diabetes. *Diabetes Care* 2007 June;30(6):1390-5.

(32) Channon SJ, Huws-Thomas MV, Rollnick S et al. A multicenter randomized controlled trial of motivational interviewing in teenagers with diabetes. *Diabetes Care* 2007 June;30(6):1390-5.

(33) Carroll AE, Marrero DG, Downs SM. The HealthPia GlucoPack Diabetes phone: a usability study. *Diabetes Technol Ther* 2007 April;9(2):158-64.

(34) Wysocki T, Harris MA, Buckloh LM et al. Randomized, controlled trial of Behavioral Family Systems Therapy for Diabetes: maintenance and generalization of effects on parent-adolescent communication. *Behav Ther* 2008 March;39(1):33-46.

(35) Sapienza JK, Masten AS. Understanding and promoting resilience in children and youth. *Curr Opin Psychiatry* 2011 July;24(4):267-73.

(36) Collins LM, Murphy SA, Bierman KL. A conceptual framework for adaptive preventive interventions. *Prev Sci* 2004 September;5(3):185-96.

(37) Sobell MB, Sobell LC. Stepped care as a heuristic approach to the treatment of alcohol problems. *J Consult Clin Psychol* 2000 August;68(4):573-9.

(38) Conduct Problems Prevention Research Group. A developmental and clinical model for the prevention of conduct disorders: The Fast Track Program. *Development and Psychology* 1992;4:509-28.

(39) Dishion TJ, Kavanagh K. A multilevel approach to family-centered prevention in schools: process and outcome. *Addict Behav* 2000 November;25(6):899-911.

(40) Borhani NO, Applegate WB, Cutler JA et al. Systolic Hypertension in the Elderly Program (SHEP). Part 1: Rationale and design. *Hypertension* 1991 March;17(3 Suppl):II2-15.

(41) Breslin FC, Sobell SL. Alcohol administration methodology 1994-1995: what researchers do and do not report about subjects and dosing procedures. *Addict Behav* 1999 July;24(4):509-20.

(42) Sobell MB, Sobell LC. Stepped care as a heuristic approach to the treatment of alcohol problems. *J Consult Clin Psychol* 2000 August;68(4):573-9.

(43) Prochaska JO, Velicer WF, Fava JL, Rossi JS, Tsoh JY. Evaluating a population-based recruitment approach and a stage-based expert system intervention for smoking cessation. *Addict Behav* 2001 July;26(4):583-602.

(44) Collins LM, Murphy SA, Bierman KL. A conceptual framework for adaptive preventive interventions. *Prev Sci* 2004 September;5(3):185-96.

(45) Wilfley DE, Tibbs TL, Van Buren DJ, Reach KP, Walker MS, Epstein LH. Lifestyle interventions in the treatment of childhood overweight: a meta-analytic review of randomized controlled trials. *Health Psychol* 2007 September;26(5):521-32.

(46) Padman R, Werk LN, Ramirez-Garnica G, Ye G, Nathanson I. Association between practice patterns and body mass index percentile in infants and young children with cystic fibrosis. *J Cyst Fibros* 2008 March 12.

(47) Jeppsson JO, Kobold U, Barr J et al. Approved IFCC reference method for the measurement of HbA1c in human blood. *Clin Chem Lab Med* 2002 January;40(1):78-89.

(48) The effect of intensive treatment of diabetes on the development and progression of long-term complications in insulin-dependent diabetes mellitus. The Diabetes Control and Complications Trial Research Group. *N Engl J Med* 1993 September 30;329(14):977-86.

(49) Intensive blood-glucose control with sulphonylureas or insulin compared with conventional treatment and risk of complications in patients with type 2 diabetes (UKPDS 33). UK Prospective Diabetes Study (UKPDS) Group. *Lancet* 1998 September 12;352(9131):837-53.

(50) Effect of intensive diabetes treatment on the development and progression of long-term complications in adolescents with insulin-dependent diabetes mellitus: Diabetes Control and Complications Trial. Diabetes Control and Complications Trial Research Group. *J Pediatr* 1994 August;125(2):177-88.

(51) Nathan DM, Cleary PA, Backlund JY et al. Intensive diabetes treatment and cardiovascular disease in patients with type 1 diabetes. *N Engl J Med* 2005 December 22;353(25):2643-53.

(52) Wysocki T, Harris MA, Buckloh LM et al. Randomized trial of behavioral family systems therapy for diabetes: maintenance of effects on diabetes outcomes in adolescents. *Diabetes Care* 2007 March;30(3):555-60.

(53) Channon SJ, Huws-Thomas MV, Rollnick S et al. A multicenter randomized controlled trial of motivational interviewing in teenagers with diabetes. *Diabetes Care* 2007 June;30(6):1390-5.

(54) Hill-Briggs F, Gemmell L. Problem solving in diabetes self-management and control: a systematic review of the literature. *Diabetes Educ* 2007 November;33(6):1032-50.

(55) Tamborlane WV, Beck RW, Bode BW et al. Continuous glucose monitoring and intensive treatment of type 1 diabetes. *N Engl J Med* 2008 October 2;359(14):1464-76.

(56) Centers for Disease Control and Prevention. CDC Growth Charts: United States. 2000.

(57) Naughton MJ, Ruggiero AM, Lawrence JM et al. Health-related quality of life of children and adolescents with type 1 or type 2 diabetes mellitus: SEARCH for Diabetes in Youth Study. *Arch Pediatr Adolesc Med* 2008 July;162(7):649-57.

(58) Seid M, D'Amico EJ, Varni JW et al. The in vivo adherence intervention for at risk adolescents with asthma: report of a randomized pilot trial. *J Pediatr Psychol* 2012 May;37(4):390-403.

(59) Markowitz JT, Butler DA, Volkening LK, Antisdel JE, Anderson BJ, Laffel LM. Brief screening tool for disordered eating in diabetes: internal consistency and external validity in a contemporary sample of pediatric patients with type 1 diabetes. *Diabetes Care* 2010 March;33(3):495-500.

(60) Iannotti RJ, Schneider S, Nansel TR et al. Self-efficacy, outcome expectations, and diabetes self-management in adolescents with type 1 diabetes. *J Dev Behav Pediatr* 2006 April;27(2):98-105.

(61) Wysocki T, Harris MA, Buckloh LM et al. Randomized trial of behavioral family systems therapy for diabetes: maintenance of effects on diabetes outcomes in adolescents. *Diabetes Care* 2007 March;30(3):555-60.

(62) Harris MA, Wysocki T, Sadler M et al. Validation of a structured interview for the assessment of diabetes self-management
3. *Diabetes Care* 2000 September;23(9):1301-4.

(63) Diabetes self-management profile for flexible insulin regimens: cross-sectional and longitudinal analysis of psychometric properties in a pediatric sample. *Diabetes Care* 2005 August;28(8):2034-5.

(64) Iannotti RJ, Nansel TR, Schneider S et al. Assessing regimen adherence of adolescents with type 1 diabetes. *Diabetes Care* 2006 October;29(10):2263-7.

(65) Wysocki T, Buckloh LM, Antal H, Lochrie A, Taylor A. Validation of a self-report version of the diabetes self-management profile. *Pediatr Diabetes* 2011 October 3.

(66) Hood KK, Butler DA, Anderson BJ, Laffel LM. Updated and revised Diabetes Family Conflict Scale. *Diabetes Care* 2007 July;30(7):1764-9.

(67) Anderson BJ, Auslander WF, Jung KC, Miller JP, Santiago JV. Assessing family sharing of diabetes responsibilities. *J Pediatr Psychol* 1990 August;15(4):477-92.

(68) Cox DJ, Irvine A, Gonder-Frederick L, Nowacek G, Butterfield J. Fear of hypoglycemia: quantification, validation, and utilization. *Diabetes Care* 1987 September;10(5):617-21.

(69) Green LB, Wysocki T, Reineck BM. Fear of hypoglycemia in children and adolescents with diabetes. *J Pediatr Psychol* 1990 October;15(5):633-41.

(70) Clarke WL, Gonder-Frederick A, Snyder AL, Cox DJ. Maternal fear of hypoglycemia in their children with insulin dependent diabetes mellitus. *J Pediatr Endocrinol Metab* 1998 March;11 Suppl 1:189-94.

(71) Songer T, Glazner J, Coombs LP et al. Examining the economic costs related to lifestyle and pharmacological interventions in youth with Type 2 diabetes. *Expert Rev Pharmacoecon Outcomes Res* 2006 June 1;6(3):315-24.

(72) Ritter PL, Stewart AL, Kaymaz H, Sobel DS, Block DA, Lorig KR. Self-reports of health care utilization compared to provider records. *J Clin Epidemiol* 2001 February;54(2):136-41.

(73) Songer T, Glazner J, Coombs LP et al. Examining the economic costs related to lifestyle and pharmacological interventions in youth with Type 2 diabetes. *Expert Rev Pharmacoecon Outcomes Res* 2006 June 1;6(3):315-24.

(74) Horsman J, Furlong W, Feeny D, Torrance G. The Health Utilities Index (HUI): concepts, measurement properties and applications. *Health Qual Life Outcomes* 2003;1:54.

(75) Radloff L. The CES-D scale: a self report depression scale for research in the general population. *Applied Psychological Measurement* 1977;1(3):385-401.

(76) The American Diabetes Association. Standards of medical care in diabetes--2012. *Diabetes Care* 2012 January;35 Suppl 1:S11-S63.

(77) Mauras N, Beck R, Xing D et al. A Randomized Clinical Trial to Assess the Efficacy and Safety of Real-Time Continuous Glucose Monitoring in the Management of Type 1 Diabetes in Young Children Aged 4 to <10 Years. *Diabetes Care* 2012 February;35(2):204-10.

(78) Miller WL RS. *Movitational interviewing: Preparing people for change*. 2 ed. New York: Guilford; 2002.

(79) Resnicow K, DiIorio C, Soet JE, Ernst D, Borrelli B, Hecht J. Motivational interviewing in health promotion: it sounds like something is changing. *Health Psychol* 2002 September;21(5):444-51.

(80) American Lung Association. Trends in Asthma Morbidity and Mortality. *American Lung Association* 2009;Available at: URL: http://www.lungusa.org/atf/cf/{7A8D42C2-FCCA-4604-8ADE-7F5D5E762256}/ASTHMA06FINAL.PDF. AccessedJune 24, 2007.

(81) D'Amico EJ, Fromme K. Brief prevention for adolescent risk-taking behavior. *Addiction* 2002 May;97(5):563-74.

(82) Werner MJ. Principles of brief intervention for adolescent alcohol, tobacco, and other drug use. *Pediatr Clin North Am* 1995 April;42(2):335-49.

(83) Brown SA. Facilitating change for adolescent alcohol problems: A multiple options approach. In: Wagner EF WH, editor. *Innovations in adolescent substance abuse intervention*.Oxford: Elsevier Science; 2001. p. 169-87.

(84) D'Amico EJ, Ellickson PL, Wagner EF et al. Developmental considerations for substance use interventions from middle school through college. *Alcohol Clin Exp Res* 2005 March;29(3):474-83.

(85) Masterman PW, Kelly AB. Reaching adolescents who drink harmfully: Fitting intervention to developmental reality. *J Subst Abuse Treat* 2003 June;24(4):347-55.

(86) D'Amico EJ, Anderson KG, Metrik J, Frissell KC, Ellingstad T, Brown SA. Adolescent self-selection of service formats: implications for secondary interventions targeting alcohol use. *Am J Addict* 2006;15 Suppl 1:58-66.

(87) Strauss A CJFS. *Chronic Illness and Quality of Life*. 2 ed. St. Louis: Mosby; 1984.

(88) D'Zurilla TJ. *Problem-solving therapy: A social competence approach to clinical intervention*. New York: Springer; 1986.

(89) Nezu AM, Perri MG. Social problem-solving therapy for unipolar depression: an initial dismantling investigation. *J Consult Clin Psychol* 1989 June;57(3):408-13.

(90) Wade S, Weil C, Holden G et al. Psychosocial characteristics of inner-city children with asthma: a description of the NCICAS psychosocial protocol. National Cooperative Inner-City Asthma Study. *Pediatr Pulmonol* 1997 October;24(4):263-76.

(91) Wigal JK, Stout C, Brandon M et al. The Knowledge, Attitude, and Self-Efficacy Asthma Questionnaire. *Chest* 1993 October;104(4):1144-8.

(92) Leroyer C, Lebrun T, Proust A et al. Knowledge, self-management, compliance and quality of life in asthma: a cross-sectional study of the French version of the Asthma Quality of Life Questionnaire. *Qual Life Res* 1998 April;7(3):267-72.

(93) Hilton S, Sibbald B, Anderson HR, Freeling P. Controlled evaluation of the effects of patient education on asthma morbidity in general practice. *Lancet* 1986 January 4;1(8471):26-9.

(94) Hill-Briggs F. Problem solving in diabetes self-management: a model of chronic illness self-management behavior. *Ann Behav Med* 2003;25(3):182-93.

(95) Yates B. *Self-Management: The Science and Art of Helping Yourself*. Belmont: Wadsworth; 1985.

(96) Varni JW, La Greca AM, Spirito A. Cognitive-behavioral interventions for children with chronic health conditions. In: Kendall PC, editor. *Child and adolescent therapy: Cognitive-behavioral procedures*. 2 ed. New York: Guilford; 2000.

(97) Evans R, III, Gergen PJ, Mitchell H et al. A randomized clinical trial to reduce asthma morbidity among inner-city children: results of the National Cooperative Inner-City Asthma Study. *J Pediatr* 1999 September;135(3):332-8.

(98) Colland VT. Learning to cope with asthma: a behavioural self-management program for children. *Patient Educ Couns* 1993 December 31;22(3):141-52.

(99) Ronchetti R, Indinnimeo L, Bonci E et al. Asthma self-management programmes in a population of Italian children: a multicentric study. Italian Study Group on Asthma Self-Management Programmes. *Eur Respir J* 1997 June;10(6):1248-53.

(100) Sahler OJ, Fairclough DL, Phipps S et al. Using problem-solving skills training to reduce negative affectivity in mothers of children with newly diagnosed cancer: report of a multisite randomized trial. *J Consult Clin Psychol* 2005 April;73(2):272-83.

(101) Howells L, Wilson AC, Skinner TC, Newton R, Morris AD, Greene SA. A randomized control trial of the effect of negotiated telephone support on glycaemic control in young people with Type 1 diabetes. *Diabet Med* 2002 August;19(8):643-8.

(102) Cook S, Herold K, Edidin DV, Briars R. Increasing problem solving in adolescents with type 1 diabetes: the choices diabetes program. *Diabetes Educ* 2002 January;28(1):115-24.

(103) Hains AA, Davies WH, Parton E, Totka J, moroso-Camarata J. A stress management intervention for adolescents with type 1 diabetes. *Diabetes Educ* 2000 May;26(3):417-24.

(104) Grey M, Boland EA, Davidson M, Li J, Tamborlane WV. Coping skills training for youth with diabetes mellitus has long-lasting effects on metabolic control and quality of life. *J Pediatr* 2000 July;137(1):107-13.

(105) Grey M, Boland EA, Davidson M, Yu C, Tamborlane WV. Coping skills training for youths with diabetes on intensive therapy. *Appl Nurs Res* 1999 February;12(1):3-12.

(106) Seid M, D'Amico EJ, Varni JW et al. The In Vivo Adherence Intervention For at Risk Adolescents With Asthma: Report of a Randomized Pilot Trial. *J Pediatr Psychol* 2011 December 13.

(107) Wysocki T. Associations among teen-parent relationships, metabolic control, and adjustment to diabetes in adolescents. *J Pediatr Psychol* 1993 August;18(4):441-52.

(108) Wysocki T, Iannotti R, Weissberg-Benchell J et al. Diabetes problem solving by youths with type 1 diabetes and their caregivers: measurement, validation, and longitudinal associations with glycemic control. *J Pediatr Psychol* 2008 September;33(8):875-84.

(109) Wysocki T, Greco P, Buckloh LM. Childhood diabetes in psychological context. In: M.C.Roberts, editor. *Handbook of pediatric psychology*. 3 ed. New York: Guilford Press; 2003. p. 599-627.

(110) Cakan N, Ellis DA, Templin T, Frey M, Naar-King S. The effects of weight status on treatment outcomes in a randomized clinical trial of multisystemic therapy for adolescents with type 1 diabetes and chronically poor metabolic control. *Pediatr Diabetes* 2007 August;8(4):206-13.

(111) Ellis DA, Yopp J, Templin T et al. Family mediators and moderators of treatment outcomes among youths with poorly controlled type 1 diabetes: results from a randomized controlled trial. *J Pediatr Psychol* 2007 March;32(2):194-205.

(112) Cameron FJ, Skinner TC, de Beaufort CE et al. Are family factors universally related to metabolic outcomes in adolescents with Type 1 diabetes? *Diabet Med* 2008 April;25(4):463-8.

(113) Botello-Harbaum M, Nansel T, Haynie DL, Iannotti RJ, Simons-Morton B. Responsive parenting is associated with improved type 1 diabetes-related quality of life. *Child Care Health Dev* 2008 September;34(5):675-81.

(114) Cakan N, Ellis DA, Templin T, Frey M, Naar-King S. The effects of weight status on treatment outcomes in a randomized clinical trial of multisystemic therapy for adolescents with type 1 diabetes and chronically poor metabolic control. *Pediatr Diabetes* 2007 August;8(4):206-13.

(115) Wysocki T, Harris MA, Greco P et al. Social validity of support group and behavior therapy interventions for families of adolescents with insulin-dependent diabetes mellitus. *J Pediatr Psychol* 1997 October;22(5):635-49.

(116) Wysocki T, Miller KM, Greco P et al. Behavior therapy for families of adolescents with diabetes: effects on directly observed family interactions. *Behavior Therapy* 1999;30(4):496-515.

(117) Wysocki T, Harris MA, Greco P et al. Randomized, controlled trial of behavior therapy for families of adolescents with insulin-dependent diabetes mellitus. *J Pediatr Psychol* 2000 January;25(1):23-33.

(118) Wysocki T, Greco P, Harris MA, Bubb J, White NH. Behavior therapy for families of adolescents with diabetes: maintenance of treatment effects. *Diabetes Care* 2001 March;24(3):441-6.

(119) Wysocki T, Harris MA, Buckloh LM et al. Effects of behavioral family systems therapy for diabetes on adolescents' family relationships, treatment adherence, and metabolic control. *J Pediatr Psychol* 2006 October;31(9):928-38.

(120) Wysocki T, Harris MA, Buckloh LM et al. Randomized trial of behavioral family systems therapy for diabetes: maintenance of effects on diabetes outcomes in adolescents. *Diabetes Care* 2007 March;30(3):555-60.

(121) Wysocki T, Harris MA, Buckloh LM et al. Randomized, controlled trial of Behavioral Family Systems Therapy for Diabetes: maintenance and generalization of effects on parent-adolescent communication. *Behav Ther* 2008 March;39(1):33-46.

(122) Wysocki T, Harris MA, Greco P et al. Social validity of support group and behavior therapy interventions for families of adolescents with insulin-dependent diabetes mellitus. *J Pediatr Psychol* 1997 October;22(5):635-49.

(123) Wysocki T, Harris MA, Greco P et al. Randomized, controlled trial of behavior therapy for families of adolescents with insulin-dependent diabetes mellitus. *J Pediatr Psychol* 2000 January;25(1):23-33.

(124) Wysocki T, Greco P, Harris MA, Bubb J, White NH. Behavior therapy for families of adolescents with diabetes: maintenance of treatment effects. *Diabetes Care* 2001 March;24(3):441-6.

(125) Wysocki T, Harris MA, Buckloh LM et al. Effects of behavioral family systems therapy for diabetes on adolescents' family relationships, treatment adherence, and metabolic control. *J Pediatr Psychol* 2006 October;31(9):928-38.

(126) Wysocki T, Harris MA, Buckloh LM et al. Randomized trial of behavioral family systems therapy for diabetes: maintenance of effects on diabetes outcomes in adolescents. *Diabetes Care* 2007 March;30(3):555-60.

(127) Wysocki T, Harris MA, Buckloh LM et al. Randomized, controlled trial of Behavioral Family Systems Therapy for Diabetes: maintenance and generalization of effects on parent-adolescent communication. *Behav Ther* 2008 March;39(1):33-46.

(128) The American Diabetes Association. Standards of medical care in diabetes--2012. *Diabetes Care* 2012 January;35 Suppl 1:S11-S63.

(129) Wysocki T, Harris MA, Buckloh LM et al. Effects of behavioral family systems therapy for diabetes on adolescents' family relationships, treatment adherence, and metabolic control. *J Pediatr Psychol* 2006 October;31(9):928-38.

(130) Wysocki T, Harris MA, Buckloh LM et al. Randomized trial of behavioral family systems therapy for diabetes: maintenance of effects on diabetes outcomes in adolescents. *Diabetes Care* 2007 March;30(3):555-60.

(131) Wysocki T, Harris MA, Buckloh LM et al. Randomized, controlled trial of Behavioral Family Systems Therapy for Diabetes: maintenance and generalization of effects on parent-adolescent communication. *Behav Ther* 2008 March;39(1):33-46.

(132) Chase HP, Maahs D. *A First Book for Understanding Diabetes*. 12th Edition ed. Denver, CO: Children's Diabetes Foundation at Denver, Colordao; 2011.

(133) Chase HP, Maahs D. *Understanding Diabetes: A handbook for people who are living with diabetes*. 12 Edition ed. Cenver, CO: Children's Diabetes Foundation at Denver, Colordao; 2011.

(134) Forsberg L, Kallmen H, Hermansson U, Berman AH, Helgason AR. Coding counsellor behaviour in motivational interviewing sessions: inter-rater reliability for the Swedish Motivational Interviewing Treatment Integrity Code (MITI). *Cogn Behav Ther* 2007;36(3):162-9.

(135) Collins LM, Murphy SA, Bierman KL. A conceptual framework for adaptive preventive interventions. *Prev Sci* 2004 September;5(3):185-96.

(136) Wysocki T, Harris MA, Buckloh LM et al. Randomized trial of behavioral family systems therapy for diabetes: maintenance of effects on diabetes outcomes in adolescents. *Diabetes Care* 2007 March;30(3):555-60.

(137) Seid M, D'Amico EJ, Varni JW et al. The In Vivo Adherence Intervention For at Risk Adolescents With Asthma: Report of a Randomized Pilot Trial. *J Pediatr Psychol* 2011 December 13.

(138) Baron RM, Kenny DA. The moderator-mediator variable distinction in social psychological research: conceptual, strategic, and statistical considerations. *J Pers Soc Psychol* 1986 December;51(6):1173-82.

(139) Fritz MS, MacKinnon DP. Required sample size to detect the mediated effect. *Psychol Sci* 2007 March;18(3):233-9.

(140) Cakan N, Ellis DA, Templin T, Frey M, Naar-King S. The effects of weight status on treatment outcomes in a randomized clinical trial of multisystemic therapy for adolescents with type 1 diabetes and chronically poor metabolic control. *Pediatr Diabetes* 2007 August;8(4):206-13.

(141) Collins LM, Murphy SA, Bierman KL. A conceptual framework for adaptive preventive interventions. *Prev Sci* 2004 September;5(3):185-96.

(142) Littell RC, Milliken GA, Stroup WW, Wolfinger RD, Schabenberger O. *SAS for mixed models*. 2nd ed. Cary, NC: SAS Institute Inc; 2006.

1. Patton JH, Stanford MS, Barratt ES. Factor structure of the Barratt impulsiveness scale. *J Clin Psychol.* 1995;51(6):768-774.
2. van Strien T, Frijters, J. E. R., Bergers, G. P. A. and Defares, P. B. . The Dutch Eating Behavior Questionnaire (DEBQ) for assessment of restrained, emotional, and external eating behavior. *Int. J. Eat. Disord.* 1986;5:295-315.
