# Supplementary material for: Assessment of a Precision Medicine Analysis of a Behavioral Counseling Strategy to Improve Adherence to Diabetes Self-management Among Youth: A Post Hoc Analysis of the FLEX Trial
Source: JAMA Netw Open. 2019 May 31;2(5):e195137. doi: 10.1001/jamanetworkopen.2019.5137 (PMC6547107; doi:10.1001/jamanetworkopen.2019.5137)
Supplement: Supplement 2. — eAppendix 1. Introduction and Measures eAppendix 2. Properties of Composite Outcome eAppendix 3. Sensitivity Analyses eAppendix 4. Future Methodological Work eTable 1. Subgroup Recovery Sensitivity and Specificity in the Simple Numerical Simulation, Synergistic Setting, for Various True Univariate Treatment Effects, by True Subgroup Status eTable 2. Treatment Effect MSE in the Simple Numerical Simulation, Synergistic Setting, by the Values of the True Univariate Treatment Effects eTable 3. Treatment Effect MSE in the Simple Numerical Simulation, Antagonistic Setting, by the Values of the True Univariate Treatment Effects eTable 4. Subgroup Recovery Sensitivity and Specificity in the Trial-Like Numerical Simulation, Synergistic Setting, for Various True Univariate Treatment Effects, by ITR Estimation Method and True Subgroup Status eTable 5. Treatment Effect MSE in the Trial-Like Numerical Simulation, Synergistic Setting, by the Values of the True Univariate Treatment Effects eTable 6. Treatment Effect MSE in the Trial-Like Numerical Simulation, Antagonistic Setting, by the Values of the True Univariate Treatment Effects eTable 7. Estimated Value and Bootstrap 95% CIs for the Value Achieved by the Estimated ITR in Each Univariate Outcome and the Composite Outcome, by Dataset and ITR Estimation Method eTable 8. Baseline Characteristics of FLEX Participants by RLT, ITR-Assigned Subgroups for the Composite Outcome and Univariate Outcomes (HbA1c Level, Quality of Life, and BMIz) eTable 9. Baseline Characteristics of FLEX Participants by Outcome Weighted Learning ITR-Assigned Subgroups for the Composite Outcome and Univariate Outcomes (HbA1c Level, Quality of Life, and BMIz) eFigure 1. Summary of Simple Numerical Experiment, Synergistic Setting eFigure 2. Summary of Simple Numerical Experiment, Antagonistic Setting eFigure 3. Comparison to Univariate Outcome, Simple Numerical Experiment, Antagonistic Setting eReferences [file jamanetwopen-2-e195137-s002.pdf]

Supplementary Online Content

Kahkoska AR, Lawson MT, Crandell J, et al. Assessment of a precision medicine analysis of a behavioral counseling strategy to improve adherence to diabetes self-management among youth: a post hoc analysis of the FLEX trial. *JAMA Netw Open*. 2019;2(5):e195137. doi:10.1001/jamanetworkopen.2019.5137

eAppendix 1. Introduction and Measures

eAppendix 2. Properties of Composite Outcome

eAppendix 3. Sensitivity Analyses

eAppendix 4. Future Methodological Work

eTable 1. Subgroup Recovery Sensitivity and Specificity in the Simple Numerical Simulation, Synergistic Setting, for Various True Univariate Treatment Effects, by True Subgroup Status

eTable 2. Treatment Effect MSE in the Simple Numerical Simulation, Synergistic Setting, by the Values of the True Univariate Treatment Effects

eTable 3. Treatment Effect MSE in the Simple Numerical Simulation, Antagonistic Setting, by the Values of the True Univariate Treatment Effects

eTable 4. Subgroup Recovery Sensitivity and Specificity in the Trial-Like Numerical Simulation, Synergistic Setting, for Various True Univariate Treatment Effects, by ITR Estimation Method and True Subgroup Status

eTable 5. Treatment Effect MSE in the Trial-Like Numerical Simulation, Synergistic Setting, by the Values of the True Univariate Treatment Effects

eTable 6. Treatment Effect MSE in the Trial-Like Numerical Simulation, Antagonistic Setting, by the Values of the True Univariate Treatment Effects

eTable 7. Estimated Value and Bootstrap 95% CIs for the Value Achieved by the Estimated ITR in Each Univariate Outcome and the Composite Outcome, by Dataset and ITR Estimation Method

eTable 8. Baseline Characteristics of FLEX Participants by RLT ITR-Assigned Subgroups for the Composite Outcome and Univariate Outcomes (HbA_{1c}, Quality of Life, and BMIz)

eTable 9. Baseline Characteristics of FLEX Participants by Outcome Weighted Learning ITR-Assigned Subgroups for the Composite Outcome and Univariate Outcomes (HbA_{1c}, Quality of Life, and BMIz)

eFigure 1. Summary of Simple Numerical Experiment, Synergistic Setting

eFigure 2. Summary of Simple Numerical Experiment, Antagonistic Setting

eFigure 3. Comparison to Univariate Outcome, Simple Numerical Experiment, Antagonistic Setting

eReferences

© 2019 Kahkoska AR et al. *JAMA Network Open.*

This supplementary material has been provided by the authors to give readers additional information about their work.

© 2019 Kahkoska AR et al. *JAMA Network Open.*

eAppendix 1. Introduction and Measures

In this supplement, we explore several technical details and sensitivity analyses, as well as presenting a variety of figures and tables, which were excluded from the main text on account of the limited available space. The supplement is structured as follows. First, we will present the details of trial measurement methodology, and we will present a rigorous mathematical definition of the trial's outcomes of interest, paying particular attention to the composite outcome which is novel to this analysis. Second, we will explore the properties of the composite outcome using numerical experiments. Third, we will present sensitivity analyses, including using the complete cases dataset rather than the imputed dataset, using outcome-weighted learning (OWL) instead of reinforcement learning trees (RLT) to estimate the optimal individualized treatment rule (ITR), and their combination, and we will present our rationale for choosing RLT in the imputed dataset as the primary method of interest. Fourth, we will introduce OWL and detail its salient differences from RLT. Fifth, we will describe the bootstrapping procedure we employed to find the variability of an ITR's value with our imputation scheme. We conclude by discussing a few potential avenues for future research.

MEASURES

In this section, we present details surrounding the measurement of key variables in the FLEX trial. We first give the details of measurement methodology for several covariates and outcomes. We then present the mathematical definitions of the four outcome variables used for our ITR estimation methods.

Measurement Methodology

Standardized Measurements. All data collection was standardized as per FLEX study protocol, and FLEX assessment staff were trained and certified to perform all study procedures. Adolescents and participating caregivers could choose to complete questionnaires online, through the secure FLEX study website, or during in-person study measurement visits. The full set of study measurements was obtained at baseline and 6 and 18 months post-randomization; a limited set of measurements was obtained at 3 and 9 months post-randomization.[1]

Laboratory Data. A central laboratory (Northwest Lipid Metabolism and Diabetes Research Laboratories, Seattle, WA, USA) provided oversight and conducted all assays. At all timepoints, hemoglobin A1c (HbA1c) was measured in whole blood by using an automated nonporous ion exchange HPLC system (model G-7; Tosoh Bioscience). Measurements of plasma cholesterol, triglycerides, and HDL cholesterol concentrations were performed on a Hitachi 917 autoanalyser (Boehringer Mannheim Diagnostics) at the full measurement visits, after the patient had fasted for at least eight hours. LDL cholesterol was calculated by the Friedewald equation for those with triglycerides <4.52 mmol/l and by the beta-quantification procedure for those with triglycerides >4.52 mmol/l.

Clinical Measures. At baseline and at 6- and 18-months post-randomization, patients wore a blinded CGM (iPro®2 Professional CGM; Medtronic Diabetes, Northridge, CA) for a seven-day period to measure interstitial glucose levels in real time throughout the day and night. Cut-points for glucose used to describe hypoglycemia were established according to recommended values.[2] Height was measured using a stadiometer, and weight was measured to the nearest 0.1 kg using an electronic scale. Body mass index (BMI, weight (kg) / height^2 (m^2)) was calculated and converted to an age- and sex-specific BMI z-score (BMIz) according to Centers for Disease Control and Prevention growth charts. Blood pressure was measured after five minutes of rest using an aneroid manometer. The second and third of three measures were averaged for systolic and diastolic pressures.

Questionnaires. Patients self-reported race, highest level of parental education, duration of diabetes, insulin delivery method (pump versus multiple daily injections (MDI)), and past use of CGM outside the study in standardized questionnaires. Self-reported race and ethnicity was classified as non-Hispanic white, non-Hispanic Black, Black, and other including Asian/Pacific Islander, Native American, or unknown. The Diabetes Self-Management Profile – Self Report (DSMP-SR) was used to assess usual practices of diabetes management during the preceding three months, across five domains: exercise, management of hypoglycemia, diet, blood glucose testing, and insulin administration and dose adjustment.[3] Higher scores indicated more diabetes self-management behaviors. The DSMP-SR was modified for the present study to allow a single questionnaire to be administered regardless of insulin regimen. Symptoms of depression were assessed using the Centers for Epidemiologic Study – Depression Scale (CES-D), with higher scores reflecting more depressive symptoms.[4] The composite Pediatric Quality of Life Inventory™– Generic Core Scales (PedsQL™) was used to assess quality of life (QoL) across four domains (physical, emotional, social, and school functioning) during the previous month, with higher scores reflecting better

QoL.[5] Fear of hypoglycemia was completed by both the adolescent and parents and measured three domains: maintaining high blood sugar, helplessness/worry about low blood sugar, and worry about negative social consequences.[6]

Outcome Variables

We first introduce notation that will be helpful in our mathematical definitions. Let $R_{1,0}$ and $R_{1,1}$ denote the vector of patient HbA1c at baseline and 18 months, respectively. Let $R_{2,0}$ and $R_{2,1}$ denote the vector of patient quality of life, as determined by the PedsQL™ Generic scale, at baseline and 18 months, respectively. Finally, let $R_{3,0}$ and $R_{3,1}$ denote the vector of patient BMI Z-score at baseline and 18 months respectively. Let $i = 1, \dots, n$ index patients, such that $R_{1,0,i}$ denotes patient *i*'s HbA1c at baseline, and so on.

We will define the three univariate outcomes before presenting the definition of the composite outcome.

HbA1c Univariate Outcome. The raw univariate outcome vector for HbA1c is simply given by $R_{1,raw} = R_{1,1} - R_{1,0}$, i.e. the diffence between HbA1c at baseline and at 18 months. Elevated HbA1c is related to the risk for long-term complications of type 1 diabetes.[7] All participants in the FLEX trial had an elevated HbA1c at baseline, meaning reductions in HbA1c are expected to reduce the risk of long-term complications. As such, the raw univariate outcome is scaled such that more negative values are preferable, as they correspond to the greatest reductions in HbA1c. By convention, the clinical reward in ITR estimation settings is strictly positive, with larger values corresponding to better rewards. We define the scaled univariate HbA1c outcome as follows:

$$R_1 = \frac{\max(R_{1,\text{raw}}) - R_{1,\text{raw}}}{\max(R_{1,\text{raw}}) - \min(R_{1,\text{raw}})}.$$

Note that by definition R_1 is restricted between 1 and 0, with larger values corresponding to better outcomes (i.e. greater reductions in HbA1c).

QoL Univariate Outcome. The raw univariate outcome vector for quality of life is given by $R_{2,raw} = R_{2,1} - R_{2,0}$, i.e. the diffence between QoL scores at baseline and at 18 months. The raw univariate outcome is scaled such that more positive values are preferable, as they correspond to the largest increases in QoL. By convention, the clinical reward in ITR estimation settings is strictly positive, with larger values corresponding to better rewards. We define the scaled univariate QoL outcome as follows:

$$R_2 = \frac{R_{2,\text{raw}} - \min(R_{2,\text{raw}})}{\max(R_{2,\text{raw}}) - \min(R_{2,\text{raw}})}.$$

Note that by definition $R_{2,0}$ is restricted between 1 and 0, with larger values corresponding to better outcomes (i.e. larger increases in QoL).

BMIz Univariate Outcome. While the raw univariate outcome for BMIz, $R_{3,raw} = R_{3,1} - R_{3,0}$, offers computational simplicity, it is not preferred because it does not take into account the patient's starting BMIz. Poor glycemic control can result in glucose purging and weight loss.[8,9] Given the elevated HbA1c levels at baseline in the FLEX study, it was expected that some participants might gain weight if glycemic control, the primary endpoint, were improved. Moreover, a substantial portion of the patients ended the trial with a BMIz in the healthy range below 1.04, some of whose BMIz did increase over the course of the 18 month period. Giving these patients a poor clinical reward is inappropriate given the relationship between glycemic control and body weight and the goals of the study. As such, we define the BMIz outcome to selectively penalize weight gain that results in excess body weight in relation to sex- and age-specific BMI percentiles. Let $R_3^{\neq 1}$ be the subvector of $R_{3,raw}$ corresponding to all *i* such that $R_{3,1,i} \geq 1.04$ and $R_{3,1,i} > R_{3,0,i}$. We consider the following constrained BMIz outcome:

$$R_3 = \begin{cases} 1, & \text{if } R_{3,1} < 1.04 \\ 1, & \text{if } R_{3,1} - R_{3,0} < 0 \\ \dfrac{\max(R_3^{\neq 1}) - R_{3,\text{raw}}}{\max(R_3^{\neq 1}) - \min(R_3^{\neq 1})}, & \text{otherwise.} \end{cases}$$

By definition, R_3 is constrained to lie between 0 and 1, with larger values corresponding to better outcomes.

© 2019 Kahkoska AR et al. *JAMA Network Open.*

Composite Outcome. We introduce the composite outcome, a combination of the three univariate outcomes into a single outcome. The composite outcome is an approximation of constrained optimization, and corresponds to a hierarchy of the univariate outcomes. Befitting the goals of the FLEX intervention, we prioritized HbA1c highest, QoL next, and BMIz third. Heuristically, the composite outcome is defined as follows. We specify thresholds for "failure" for HbA1c and QoL, such that a patient is considered to have unacceptable values of that outcome if they fall on the wrong side of that threshold at the end of the study. Patients who fail HbA1c, regardless of their QoL and BMIz, are placed into the first category and take the lowest numerical reward values, between 0 and 1, with the magnitude determined by how poor their HbA1c is. Patients who have an acceptable HbA1c but fail QoL are placed into the second category, with numerical reward values falling between 1 and 2 depending on how poor their QoL is. Finally, patients who end the trial with acceptable HbA1c and QoL are placed into the third category and given the highest numerical values, with values ranging from 2 to 3 depending on how much their BMIz improved. Both failure criteria can be circumvented by strong enough improvement---for instance, a patient whose HbA1c at 18 months is 9.0 but whose HbA1c fell by 0.7 over the course of the intervention is not considered to have failed HbA1c for the purposes of the composite outcome.

We define the mutually exclusive outcome threshold events E_1, E_2, E_3 to simplify notation. E_1 is the indicator that $R_{1,1} > 8.5$ and $R_{1,raw} > -0.5$. E_2 is the indicator that $E_1 = 0$, $R_{2,1} < 60$, and $R_{2,raw} < 10$. E_3 is the indicator that both E_1 and E_2 equal 0. The thresholds for HbA1c were chosen based on clinical cut-points, and the thresholds for QoL were chosen based on sample quantiles of QoL in the sample. The composite outcome is defined as follows:

$$R = \begin{cases} \dfrac{R_{1,1} - 8.5}{\max(R_{1,1}) - 8.5}, & E_1 = 1 \\ 1 + \dfrac{60 - R_{2,1}}{60 - \min(R_{2,1})}, & E_2 = 1 \\ 2 + R_3, & E_3 = 1. \end{cases}$$

© 2019 Kahkoska AR et al. *JAMA Network Open.*

eAppendix 2. Properties of Composite Outcome

In this section, we explore the properties of the composite outcome. The composite outcome is a method of approximating constrained optimization over multiple outcome variables, and to our knowledge represents a novel approach to doing so. Constrained problems are of frequent interest in medicine, particularly in complex diseases where it is unlikely for one outcome variable to dominate all others. Type 1 diabetes alone, for instance, presents many sets of outcome variables that lend themselves to a constrained approach. Investigators may wish to enforce ideal glycemic control while discouraging weight gain, a known side effect of intensive insulin therapies[9]; they may wish to lower long-term measures of glycemic control such as HbA1c while simultaneously keeping patients out of the acutely dangerous hypoglycemic range measured by a continuous glucose monitor; or they may have more complex sets of goals corresponding to several outcome variables, as is the case in the FLEX trial.

As the mathematical definition presented in the prior section suggests, the distribution of the composite outcome depends directly upon an explicit hierarchy of the outcome variables in a trial, as well as meaningful regional thresholds that define failure events for those outcome variables. Both the hierarchy and the thresholds rely on domain knowledge. In the FLEX trial, for instance, domain knowledge suggested the regions of interest for one of our outcome variables—HbA1c below 8.5 is considered a significant improvement for this population, which was recruited with high baseline HbA1c[10]—and informed the other, while the priorities of the trial dictated the order of the hierarchy. HbA1c was prioritized highest, as it was the outcome of primary interest and directly related to long-term complications of diabetes. QoL was prioritized second, as it was an outcome of secondary interest and represents an important patient-oriented outcome. Due to natural growth in this age range complicating BMI-based outcomes, and due to the complicated relationship between body weight and glycemic control alluded to in the definition of the BMIz outcome, BMIz was prioritized after both HbA1c and QoL.[1] Note that the shape of the regions need not always be as simple as those presented in the FLEX trial---failure thresholds for raw BMI, for example, would likely penalize measurements that are too high as well as those that are too low.

This approach offers one main advantage over constrained optimization: ease of use. Constrained optimization can be difficult to implement, especially when the regions of interest are complex, and the burden introduced by considering additional outcome variables can be far from trivial. So long as the hierarchy and the failure regions remain clear, it is simple to add outcome variables into a composite outcome. Any analyst equipped to carry out ITR estimation should be equipped to create a composite outcome and carry out an almost exactly analogous analysis with it; the same cannot be said of coding constrained optimization by hand.

Numerical Experiments

We examine the finite-sample performance of the composite outcome for ITR-based subgroup determination in a trial with heterogeneous treatment effects through a brief set of numerical experiments. In the first, we examine a very simple model to explore the properties of the method and the composite outcome, particularly regarding the muted group. In the second, we examine the performance of the method in settings more akin to those likely to be observed in real studies, paying special attention to comparing the performance of RLT and OWL-based subgroups.

Several features are similar across experiments. In both cases, the covariates X are drawn i.i.d. from U(-1,1), with a corresponding Gaussian p+1-vector β that governs the baseline link between covariates and clinical reward R. The observed treatment A is chosen independent of covariates and rewards so that patients are randomized equally to intervention ($A_i = 1$) and control ($A_i = -1$). Each experiment has two clinical rewards,R_1 and R_2. We specify true subgroups for each patient, stored in the n-vector S, where $S \in \{-1,0,1\}$ denotes whether the patient benefits from control, has equivalent reward under control and treatment, or benefits from treatment, respectively. We assume that these subgroups apply to each outcome for the sake of simplifying comparisons to the gold standard; the details of how each subgroup is determined varies between experiments. R_1 and R_2 are constructed similarly across experiments. Given fixed treatment effects δ_1 and δ_2 for R_1 and R_2 respectively, each experiment considers two settings. In the first, termed *synergistic*, the treatment effects point in the same direction for both outcomes:

$$R_j = X\beta + \epsilon + \delta_j AS,$$

for $j = 1,2$. In the reverse scenario, termed *antagonistic*, the treatment effects point in opposite ways for the two outcomes:

$$R_1 = X\beta + \epsilon + \delta_1 AS$$
$$R_2 = X\beta + \epsilon - \delta_2 AS.$$

The threshold q defines the failure event for R_1: patients with $R_1 < q$ have "failed" R_1 and receive composite outcome $R \in [0,1]$ based on the magnitude of their R_1, while patients with $R_1 \geq q$ have "acceptable" R_1 and receive $R \in [1,2]$ based on the magnitude of their R_2, in the manner described in the originally stated definition of the composite outcome. We set q to the first quartile of R_1 in the sequel. In each simulation, once the data were generated, we estimated the optimal ITR in the sample using RLT (and, in the second experiment, OWL), then used it to obtain subgroup estimates $\hat{S}$. One set of evaluation metrics for the method is the subgroup recovery sensitivity and specificity, defined as

$$\text{sens}_j = \frac{\sum_{i=1}^{n}(\hat{S}_i = j \cap S_i = j)}{\sum_{i=1}^{n} S_i = j}$$

$$\text{spec}_j = \frac{\sum_{i=1}^{n}(\hat{S}_i \neq j \cap S_i \neq j)}{\sum_{i=1}^{n} S_i \neq j},$$

where $j = -1,0,1$. Another evaluation metric is available for the RLT ITR due to the simulated nature of the data. Let R^1 and R^{-1} denote the true values of R under intervention and control. Since we know the magnitude and direction of the true treatment effect for both R_1 and R_2, we can calculate the difference in R obtained by switching a patient on intervention to control or vice versa. For instance, in the synergistic setting, a patient with $S_i = 1$ and $A_i = 1$ would have $R_{i1}^{-1} = R_{i1} - \delta_1$ and $R_{i2}^{-1} = R_{i2} - \delta_2$. Then R_i^{-1} could be obtained by recalculating R with these perturbed values. Let $\hat{Q}^1(X)$ and $\hat{Q}^{-1}(X)$ denote the RLT-predicted values of R under treatment and control, respectively. Then we can examine the mean squared error (MSE) for the treatment effect, defined as

$$\text{MSE} = n^{-1}\sum_{i=1}^{n}\left[\left(R_i^1 - R_i^{-1}\right) - \left(\hat{Q}^1(X_i) - \hat{Q}^{-1}(X_i)\right)\right]^2.$$

Experiment in Simple Conditions. We first explored a basic model to assess the performance of the method when all factors are straightforward, particularly with regards to the muted group. In this model, we set *n*=200 and *p*=1.

As briefly discussed in the prior section, we intentionally built a muted group into our simulated data. In particular, we allotted the true subgroup membership according to

$$S = \begin{cases} 1, & X > 0.5 \\ -1, & X < -0.5 \\ 0, & \text{otherwise.} \end{cases}$$

In this experiment, we considered both the synergistic and antagonistic setting, and we considered three values for each δ_j: 1, 3, and 10.

As our goal in this simulation was to examine the behavior of the method surrounding the muted group, we used only RLT to estimate the optimal ITR.

eTable 1 gives the subgroup recovery sensitivity and specificity in the synergistic setting by δ_1, δ_2, and value of S. Overall, we see high sensitivity for both the intervention and control group, especially when the treatment effect for either outcome variable is large. Specificity is less impressive in these groups for large and small treatment effects alike. This is attributable to a curious phenomenon: in none of these simulations did the proposed method recover a muted group. While this seems an indictment of the method, examining the treatment effect MSE in the synergistic setting, displayed in eTable 2, reveals a different story: the treatment effect MSE is small even when the treatment effects are modest, and very small when treatment effects are large, which suggests the method is performing well. eFigure 1 illustrates the disconnect between the conclusions drawn from these evaluation metrics. In particular, in the true domain of the muted group, a method that performs perfectly would have $\hat{Q}^1(X_i) - \hat{Q}^{-1}(X_i) = 0$ within this range. The method does not set any of these differences identically equal to zero for any combination of (δ_1, δ_2); however, for each (δ_1, δ_2), the estimated differences are smaller in this range than outside it, with this trend increasing as the true treatment effect increases. We will return to this observation in our discussion of the numerical experiments.

eTable 3 gives the treatment effect MSE in the antagonistic setting. Again, MSE is low throughout, suggesting the method performs well even in this conceptually more difficult setting. eFigure 2 illustrates the predictions and

© 2019 Kahkoska AR et al. *JAMA Network Open.*

estimated vs. true treatment effects plotted against the true splitting variable X in the antagonistic setting. Again, the predicted differences between interention and control are smaller in magnitude inside the range of the true muted group than outside it, on average, though none are set identically to zero. We also note the somewhat unintuitive behavior of the true treatment effects: although the treatment effect is truly positive for one of the outcome variables, nearly all the true treatment effects are negative. Optimizing on either outcome in a univariate manner would fail to discover this interesting trend, as eFigure 3 demonstrates: the trend for the univariate outcome appears similar to that exhibited in the synergistic setting.

Experiment in Trial-Like Conditions. We briefly explore the performance of the method in conditions more similar to those observed in the FLEX trial. In this experiment, we set *n*=200 and *p*=30.

As in the simple simulation of the preceding section, we divide the sample into a true intervention, control, and muted group. The subgroup divisions are given by

$$S = \begin{cases} -1, & a^t X_{k,\text{int}} < c_1 \\ 1, & a^t X_{k,\text{int}} > c_2 \\ 0, & \text{otherwise,} \end{cases}$$

where $X_{k,int}$ is a matrix composed of the first k columns of X and all their pairwise interactions, a is a random Gaussian vector of the corresponding length, and c_1 and c_2 are specified thresholds. In the sequel, we choose *k*=2 and set c_1 and c_2 to the first and third quartiles of $a^T X_{k,int}$, respectively. In this experiment, we considered both the synergistic and antagonistic setting, and we considered three values for the true univariate treatment effects δ_1 and δ_2: 1, 3, and 10. Additionally, we considered both RLT and OWL to estimate the optimal ITR. We ran 100 simulations for each combination of ITR method, δ_1, δ_2, and setting.

eTable 4 gives the average subgroup recovery sensitivity and specificity over the 100 simulations for both RLT and OWL for the synergistic setting, divided by true subgroup status and true value of the univariate treatment effects δ_1 and δ_2. Both methods show improvements in sensitivity for the intervention and control groups as the true treatment effect sizes increase. RLT once again struggles to pick up a muted group in any setting, failing to do so entirely when effect sizes are large. Unlike in the numerical experiment of the prior section, RLT does succeed in discovering a muted group with high specificity when effect sizes are small, albeit with modest sensitivity. As noted before, OWL is incapable of discovering a muted group; its sensitivity and specificity remain admirably high in the groups it can recover.

eTables 5 and 6 give the average treatment effect MSE over the 100 simulations by true value of univariate treatment effects δ_1 and δ_2 for the synergistic and antagonistic settings, respectively. As in the prior numerical experiment, MSE values are generally low in this numerical experiment, especially in the synergistic setting, suggesting that the method performs well at estimating the true treatment effect in trial-like settings even when its performance in identifying members of the muted group appears to suffer.

Discussion of Numerical Experiments. One salient take-away from the simple numerical experiment, especially the disconnect between subgroup recovery sensitivity and specificity and treatment effect MSE illustrated by eFigure 1, is that slight alterations to this method may lead to improvements in its performance as measured by subgroup recovery sensitivity and specificity. In real data, we will not know the true treatment effect—the method will, for better or worse, be judged on its efficacy in determining the correct subgroup label. In this experiment, the method reliably estimated much smaller treatment effects in the range defined by the muted group than the range defined by the intervention and control groups, but failed to set these effects identically equal to zero. An "ε-insensitive" version of the method, in which predicted values under treatment and control must differ by at least ε to be placed into either the control or intervention group, might offer improved performance with the same attractive interpretability.

Another notable trend observed in both numerical experiments is the high specificity for the muted group even when effect sizes were small. This fact may increase our confidence in the existence of the muted group assigned by RLT in the FLEX trial, while the satisfactory sensitivity and specificity for both the intervention and control groups in numerical experiments is promising for those groups as well.

eAppendix 3. Sensitivity Analyses

In this section, we present sensitivity analyses pertaining to two of our methodological decisions: the decision to impute missing covariates with MICE, and the decision to use RLT to compute the estimated optimal ITR. We address the former by carrying out the method on the subset of patients with complete cases in all covariates and outcomes; we address the latter by estimating the optimal ITR with OWL. As such, we ultimately explored four settings: complete cases and RLT; complete cases and OWL; imputed cases and RLT; and imputed cases and OWL.

eTable 7 gives $\hat{V}_{opt}$ for each combination of dataset, ITR estimation method, and outcome trained upon. Each column of eTable 7 has its own natural numerical scale, due to the distribution of the underlying rewards. The combination of method and dataset that performed the best for each outcome, in terms of value, is shown in bold. 95% confidence intervals for each value estimate are given by the bootstrap, described in fuller detail in the penultimate section of this supplement. For the three univariate outcomes, the different ITRs show only minor differences in value. OWL in the imputed dataset performs nominally best for HbA1c and QoL, while OWL in the complete cases performs nominally best for BMIz. Of these comparisons, only one seems to indicate a significant difference: RLT in the imputed dataset appears to perform worse than OWL in the imputed dataset for the univariate HbA1c outcome, with the former's 95% CI lying entirely above the latter's. For the composite outcome, however, RLT in the imputed dataset provides a substantial improvement in value over all other estimated ITRs, with its 95% CI lying well above all other ITR's.

This improvement in value is the primary reasons we chose RLT in the imputed dataset as the primary ITR estimation method of interest. The others are more philosophical in nature. RLT's creation of the muted group, as described in the main text, is another argument in favor of RLT—the muted group may prove to be an important consideration for real-life decisions about targeting interventions. In particular, the fact that the majority of adolescents are “indifferent” to the FLEX intervention with regards to HbA1c, as Table 3 in the main text demonstrates, is a reflection of the challenges in controlling glycemia within the age range studied by the trial, as well as an indication that future work is needed to better tailor the FLEX intervention toward the specific needs of adolescents. Additionally, the arguments that typically apply to imputation—it allows us to use more data, and the assumptions required to impute are least impactful when the amount of total observations filled in by imputation is small—apply to the FLEX trial as well.

For completeness, we present eTable 8, the “full” version of Table 3 in the main text with all covariates that were considered instead of only those that had significant differences between subgroups for at least one ITR. We also present eTable 9, the analogue of eTable 8 for the subgroups defined by OWL in the imputed dataset. For conciseness, we do not present the analogous tables for the ITRs estimated in the complete cases dataset.

eTable 9 depicts the characteristics of FLEX participants in the subgroups assigned by the OWL ITR to the Intervention and Usual Care for the composite outcome and each univariate outcome.

Regarding the composite outcome, 101 participants (47%) were assigned to Intervention, while the remaining 115 participants (53%) were assigned to Usual Care. Individuals assigned to the Intervention subgroup were less likely to have private health insurance (57% versus 82%; P = 0.001). Participants assigned to Intervention had a longer disease duration at baseline (mean (SD) 7.3 (3.7) years versus 5.5 (3.5) years; P = 0.02) and were less likely to use an insulin pump (57% versus 83.5%; P < 0.001). They also reported lower problem-solving abilities at baseline (mean (SD) SPSI score of 103.2 (13.0) versus 108.4 (12.5); P =0.02).

Regarding the HbA1c univariate outcome, 118 participants (55%) were assigned to Intervention and 98 participants (45%) were assigned to Usual Care. Individuals assigned to Intervention were more likely to be non-Hispanic white race/ethnicity (91% versus 60%; P < 0.01) and less likely to have private health insurance (60% versus 83%, P = 0.04).

Regarding the QoL univariate outcome, 123 participants (57%) were assigned to Intervention and 93 participants (43%) were assigned to Usual Care. Individuals assigned to Intervention showed higher glycemic variability (mean (SD) coefficient of variation of 41.5% (8.0%) versus 37.9% (7.4%) and experienced more clinical and clinically serious hypoglycemia at baseline (all P < 0.05).

© 2019 Kahkoska AR et al. *JAMA Network Open.*

Regarding the BMIz univariate outcome, 116 participants (54%) were assigned to Intervention and 100 participants (46%) were assigned to Usual Care. Individuals assigned to Intervention showed a longer disease duration at baseline (mean (SD) 7.8 (3.8) months versus 4.7 (3.0) months; P < 0.001).

Compared to RLT (depicted in eTable 8), the OWL ITR (eTable 9) assigned a larger proportion of the FLEX sample to the intervention group, ranging from 47% for the composite outcome to 57% for quality of life. Characteristics that were significantly different across RLT-assigned subgroups were not consistent with the characteristics that were significantly different across the OWL-assigned subgroups. In some cases, the OWL-assigned subgroups showed significant differences in additional characteristics. For example, individuals assigned to Intervention for the composite were less likely to have public health insurance with both ITR, but those in the OWL-assigned group showed additional differences in sex, race/ethnicity, disease duration, and insulin pump use versus the participants that OWL assigned to receive Usual Care. In other cases, differences across the RLT-assigned subgroups were not replicated across the OWL-assigned subgroups. This may be in part due to redistribution of individuals from the Muted Group in RLT subgroups into the Intervention Group or Usual Care groups estimated by OWL. For example, the individuals muted by RLT for the HbA1c univariate outcome had significantly higher HbA1c at baseline compared to the Intervention and Usual Care Groups. By contrast, the OWL-assigned subgroups showed no differences in HbA1c, although both groups showed consistent differences in hypoglycemia at baseline.

OUTCOME WEIGHTED LEARNING

Outcome weighted learning (OWL) is a precision medicine method for estimating the optimal ITR in a sample. Unlike RLT, which poses a model between X and R and inverts it to find the ITR, OWL considers a class of functions Π to which all estimated ITRs can belong, then directly estimates the optimal ITR $\hat{\pi}_{opt}$ by minimizing a loss function applied to this class.[11] The challenge in implementing OWL comes from specifying a class Π that is robust enough to allow sufficiently close estimation of the true optimal ITR π^*_{opt} but also computationally tractable. The specific implementation of OWL we employed was Residual Weighted Learning with a linear kernel.[12]

One key difference between RLT and OWL is that OWL does not create a muted group. Residual weighted learning ultimately relies on support vector machines, which are strict classifiers—all observations are assigned to either treatment or control.

This implies that OWL and RLT may be suited to different scenarios. In scenarios where the true muted group is small, OWL's focus on direct estimation of the ITR may lead to improvements in overall value. But in scenarios where the true muted group is large, OWL will classify them into either intervention or control regardless of the estimated treatment effect. This may impact value estimates, but more importantly misrepresents the efficacy of both intervention and control. And naturally, any attempt to characterize the ITR-based treatment or control group through their covariates will be highly influenced by having additional group members under OWL, especially group members whose differential treatment response suggests they may increase the heterogeneity of the group.

IMPUTATION BOOTSTRAPPING PROCEDURE

In this section we describe the bootstrapping procedure we employed to estimate the variability of an ITR which was estimated from multiple imputed datasets. We begin by restating, and introducing notation for, our multiple imputation procedure.

Let $k = 1, \dots, K$ index the K datasets imputed by MICE, $X_{imp,k}$. As noted in the main text, we used $K = 11$ to preclude the possibility of ties on a majority vote for OWL, but we leave K general here. As before, let $i = 1, \dots, n$ index patients and $j = 1, \dots, p$ index covariates, such that each $X_{imp,k}$ is an n by p matrix. Let $\hat{\pi}_{opt,k}$ denote the estimated optimal ITR estimated from the data $X_{imp,k}$, and let $\hat{V}_{opt,k}$ denote its estimated value. Let the estimated optimal ITR for the whole sample be denoted $\hat{\pi}_{opt}$, with estimated value $\hat{V}_{opt}$. Our multiple imputation procedure follows the steps outlined in Algorithm 1.

© 2019 Kahkoska AR et al. *JAMA Network Open.*

Algorithm 1 Multiple imputation procedure for $\hat{\pi}_{\text{opt}}$

1. Generate $X_{\text{imp},k}, k = 1, \ldots, K$, via MICE
2. For $k = 1, \ldots, K$, compute $\hat{\pi}_{\text{opt},k}$ via the method of interest
3. Set $\hat{\pi}_{\text{opt}}$ to the plurality vote of the $\hat{\pi}_{\text{opt},k}$
4. Obtain the estimated value $\hat{V} = \left(\sum_i R_i I\left\{A_i = \hat{\pi}_{\text{opt}}(X_i)\right\}\right) / \left(\sum_i I\left\{A_i = \hat{\pi}_{\text{opt}}(X_i)\right\}\right)$

We can use Algorithm 1 to obtain point estimates of $\hat{V}_{opt}$. However, due to multiple imputation, large-sample theory no longer provides a simple form for an estimate of the variability of $\hat{V}_{opt}$. Although the bootstrap is not guaranteed to be valid in all precision medicine settings, under reasonable regularity conditions for the estimated ITR, bootstrap validity will hold.

The first and second steps of Algorithm 1 are, unsurprisingly, its most computationally intensive. To avoid replicating as many computationally intensive steps as possible when bootstrapping, we adopt the bootstrapping procedure described in Algorithm 2.

Algorithm 2 Bootstrapping to estimate the variability of V_{opt}

1. Sample the indices $i = 1, \ldots, n$ B times with replacement to generate the bootstrapping index vectors, $I_b, b = 1, \ldots, B$
2. For $b = 1, \ldots, B$:
   (a) For $k = 1, \ldots, K$:
      i. Generate the bth bootstrap sample of the kth imputed dataset $\tilde{X}^b_{\text{imp},k}$, by stacking the rows of $X_{\text{imp},k}$ corresponding to I_b
      ii. Compute $\hat{\pi}^b_{\text{opt},k}$ via the method of interest
   (b) Set $\hat{\pi}^b_{\text{opt}}$ to the plurality vote of the $\hat{\pi}^b_{\text{opt},k}$
   (c) Compute $\hat{V}^b_{\text{opt}} = \left(\sum_i R_i I\left\{A_i = \hat{\pi}^b_{\text{opt}}(X_i)\right\}\right) / \left(\sum_i I\left\{A_i = \hat{\pi}^b_{\text{opt}}(X_i)\right\}\right)$
3. Compute $\widehat{SE}(\hat{V}_{\text{opt}}) = \frac{1}{B}\sum_{b=1}^{B}\left(\hat{V}^b_{\text{opt}} - \bar{V}_{\text{opt}}\right)$

Strictly speaking, the bootstrapping procedure in Algorithm 2 does not capture all of the variability that arises from MICE, in the sense that we do not impute via MICE after generating each bootstrap sample. As the proportion of missing data in any one column in this sample was quite low, however, we did not believe capturing this particular source of variance was crucial given the computational costs it would incur.

Bootstrap procedures to estimate the confidence interval of a single ITR's value, or the difference in estimated value between two ITRs, proceed in a manner analogous to Algorithm 2, following the usual bootstrapping recommendations that B should be higher to estimate a confidence interval than a variance. As the procedure is entirely analogous, we do not explicitly outline it here. For the sensitivity analyses presented in these supplementary materials, we calculated 95% CIs based on $B = 1000$ bootstrap replicates.

© 2019 Kahkoska AR et al. *JAMA Network Open.*

eAppendix 4. Future Methodological Work

We briefly mentioned one potential direction for future research in our discussion of numerical experiments. Namely, as eFigures 1, 2, and 3 demonstrate, subgroup recovery sensitivity and specificity, particularly sensitivity regarding the muted group and specificity regarding the intervention and control groups, may be improved by an ITR estimation method that is less sensitive to predicted differences between treatment and control. In the case of an indirect estimation method like RLT, this may take the form of ε-insensitivity to differences in predicted values. That is, where the RLT-based ITR is currently assigned by

$$\hat{\pi}(X_i) = \begin{cases} -1, & \hat{Q}^{-1}(X_i) - \hat{Q}^{1}(X_i) > 0 \\ 0, & \hat{Q}^{-1}(X_i) - \hat{Q}^{1}(X_i) = 0 \\ 1, & \hat{Q}^{-1}(X_i) - \hat{Q}^{1}(X_i) < 0, \end{cases}$$

it would instead be assigned by

$$\hat{\pi}(X_i) = \begin{cases} -1, & \hat{Q}^{-1}(X_i) - \hat{Q}^{1}(X_i) > \epsilon \\ 0, & |\hat{Q}^{-1}(X_i) - \hat{Q}^{1}(X_i)| \leq \epsilon \\ 1, & \hat{Q}^{-1}(X_i) - \hat{Q}^{1}(X_i) < -\epsilon, \end{cases}$$

where $\hat{Q}^1(X)$ and $\hat{Q}^{-1}(X)$ are defined as in the introduction to our numerical experiments. The details of finding an automated procedure to choose the most desirable ε—and indeed the correct metric to evaluate the desirability of a given choice of ε—remain for future research. Extending this logic to direct estimation methods such as OWL presents further challenges. Methods exist providing an option to "reject" the choice between intervention and control in an OWL framework by penalizing misclassification more heavily than failing to classify. While this procedure creates a third group, the algorithmic justification of this group is not identical to that of the Muted Group defined by RLT's variable muting. The "rejection" group consists of those patients who are likely to be misclassified if the algorithm is forced to choose; the muted group described in this work consists of those patients who experience no difference in predicted outcome between intervention and control (or, if the algorithm is ε-insensitive, a sufficiently small difference). It is reasonable to presume that patients likely to be misclassified by OWL are also likely to experience no (or very small) RLT-predicted differences between intervention and control. This is, however, merely a presumption, and it must be verified.

Another natural extension of the work presented in this manuscript is to more complex intervention options than binary. For indirect estimation methods such as RLT, the *K*-intervention setting does not induce a particular mathematical burden in ITR estimation, with the exception of likely needing to scale any ε adaptively by intervention pair. The concept of the muted group, however, must be extended to "muted for a given intervention set." A patient may, for instance, experience identical predicted outcomes under interventions A and B but identical, higher predicted outcomes under interventions C and D. Such a case clearly recommends one set of interventions over another set, but does not distinguish between the interventions within either set. In the same way, extending indirect estimation methods to the ordinal treatment or dose-finding setting may not pose especially great mathematical challenges, but the challenges in interpretation are non-trivial. For the direct estimation method of OWL, extensions to the *K*-treatment, ordinal, and dose-finding, settings that do not consider a muted group are feasible given existing methods.[13,14,15] None of these methods are currently adapted to include a "rejection" region, and the ordinal treatment and dose-finding settings in particular would seem to present mathematical challenges for doing so. Even still, the potential for disconnect between the muted group and the "rejection" group remains here.

© 2019 Kahkoska AR et al. *JAMA Network Open.*

eTable 1. Subgroup Recovery Sensitivity and Specificity in the Simple Numerical Simulation, Synergistic Setting, for Various True Univariate Treatment Effects, by True Subgroup Status

δ_1	δ_2	Measure	$S = -1$	$S = 0$	$S = 1$
1	1	Sens	0.962	0	0.917
1	1	Spec	0.541	1	0.750
1	3	Sens	0.981	0	0.1
1	3	Spec	0.581	1	0.743
1	10	Sens	1	0	1
1	10	Spec	0.669	1	0.664
3	1	Sens	1	0	0.917
3	1	Spec	0.547	1	0.757
3	3	Sens	1	0	1
3	3	Spec	0.581	1	0.750
3	10	Sens	1	0	1
3	10	Spec	0.581	1	0.776
10	1	Sens	1	0	0.938
10	1	Spec	0.534	1	0.776
10	3	Sens	1	0	1
10	3	Spec	0.601	1	0.730
10	10	Sens	1	0	1
10	10	Spec	0.588	1	0.743

© 2019 Kahkoska AR et al. *JAMA Network Open.*

eTable 2. Treatment Effect MSE in the Simple Numerical Simulation, Synergistic Setting, by the Values of the True Univariate Treatment Effects

δ_1	Treatment effect MSE when:		
	$\delta_2 = 1$	$\delta_2 = 3$	$\delta_2 = 10$
1	0.054	0.035	0.053
3	0.040	0.027	0.015
10	0.036	0.022	0.012

© 2019 Kahkoska AR et al. *JAMA Network Open.*

eTable 3. Treatment Effect MSE in the Simple Numerical Simulation, Antagonistic Setting, by the Values of the True Univariate Treatment Effects

$\boldsymbol{\delta_1}$	**Treatment effect MSE when:**		
	$\boldsymbol{\delta_2 = 1}$	$\boldsymbol{\delta_2 = 3}$	$\boldsymbol{\delta_2 = 10}$
1	0.035	0.049	0.057
3	0.027	0.040	0.069
10	0.030	0.043	0.066

© 2019 Kahkoska AR et al. *JAMA Network Open.*

eTable 4. Subgroup Recovery Sensitivity and Specificity in the Trial-Like Numerical Simulation, Synergistic Setting, for Various True Univariate Treatment Effects, by ITR Estimation Method and True Subgroup Status

δ_1	δ_2	Measure	ITR Est. Method: RLT			ITR Est. Method: OWL		
			$S = -1$	$S = 0$	$S = 1$	$S = -1$	$S = 0$	$S = 1$
1	1	Sens	0.445	0.293	0.427	0.722	0	0.719
1	1	Spec	0.678	0.705	0.698	0.610	1	0.609
1	3	Sens	0.719	0.067	0.801	0.810	0	0.824
1	3	Spec	0.706	0.934	0.650	0.667	1	0.646
1	10	Sens	0.928	0	0.941	0.866	0	0.876
1	10	Spec	0.740	1	0.690	0.693	1	0.674
3	1	Sens	0.964	0.009	0.718	0.929	0	0.727
3	1	Spec	0.521	0.992	0.830	0.550	1	0.775
3	3	Sens	0.986	0	0.934	0.938	0	0.868
3	3	Spec	0.678	1	0.778	0.654	1	0.744
3	10	Sens	0.991	0	0.983	0.945	0	0.919
3	10	Spec	0.723	1	0.759	0.697	1	0.730
10	1	Sens	0.999	0	0.832	0.991	0	0.624
10	1	Spec	0.568	1	0.843	0.436	1	0.868
10	3	Sens	0.999	0	0.972	0.984	0	0.835
10	3	Spec	0.671	1	0.809	0.596	1	0.809
10	10	Sens	0.999	0	0.998	0.974	0	0.939
10	10	Spec	0.715	1	0.778	0.702	1	0.750

© 2019 Kahkoska AR et al. *JAMA Network Open.*

eTable 5. Treatment Effect MSE in the Trial-Like Numerical Simulation, Synergistic Setting, by the Values of the True Univariate Treatment Effects

δ_1	Treatment effect MSE when:		
	$\delta_2 = 1$	$\delta_2 = 3$	$\delta_2 = 10$
1	0.109	0.129	0.139
3	0.153	0.137	0.119
10	0.091	0.080	0.055

© 2019 Kahkoska AR et al. *JAMA Network Open.*

eTable 6. Treatment Effect MSE in the Trial-Like Numerical Simulation, Antagonistic Setting, by the Values of the True Univariate Treatment Effects

$\boldsymbol{\delta_1}$	**Treatment effect MSE when:**		
	$\boldsymbol{\delta_2 = 1}$	$\boldsymbol{\delta_2 = 3}$	$\boldsymbol{\delta_2 = 10}$
1	0.099	0.107	0.123
3	0.150	0.146	0.137
10	0.094	0.104	0.141

© 2019 Kahkoska AR et al. *JAMA Network Open.*

eTable 7. Estimated Value and Bootstrap 95% CIs for the Value Achieved by the Estimated ITR in Each Univariate Outcome and the Composite Outcome, by Data Set and ITR Estimation Method

**Dataset**	**Method**	**$\widehat{V}$ (95% CI) for Outcome**			
		**HbA1c**	**QoL**	**BMIz**	**Composite**
Complete case	RLT	0.6786 (0.660, 0.702)	0.6434 (0.643, 0.702)	0.9670 (0.954, 0.984)	2.1359 (2.030, 2.272)
Complete case	OWL	0.6905 (0.693, 0.707)	0.7006 (0.698, 0.722)	**0.9857 (0.984, 0.989)**	2.0734 (2.060, 2.167)
Imputed	RLT	0.6738 (0.661, 0.689)	0.6739 (0.669, 0.722)	0.9737 (0.960, 0.988)	**2.6985 (2.696, 2.858)**
Imputed	OWL	**0.7044 (0.702, 0.705)**	**0.7021 (0.701, 0.703)**	0.9855 (0.985, 0.986)	1.9867 (1.959, 2.070)

© 2019 Kahkoska AR et al. *JAMA Network Open.*

eTable 8. Baseline Characteristics of FLEX Participants by Reinforcement Learning Trees (RLT), Individualized Treatment Rule (ITR)-Assigned Subgroups for the Composite Outcome and Univariate Outcomes (HbA_{1c}, quality of life, and BMIz)

	All	A. Composite Outcome			B. Univariate Outcome: HbA1c				C. Univariate Outcome: Quality of Life				D. Univariate Outcome: BMIz			
Baseline characteristics, n (%) or mean (SD)		Intervention Group, n=91 (42.1%)	Control Group, n= 125 (57.9%)	p-value	Intervention Group, n=54 (25.0%)	Muted Group, n=105 (48.6%)	Control Group, n=57 (26.4%)	p-value	Intervention Group, n=89 (41.2%)	Muted Group, n=63 (29.2%)	Control Group, n=64 (29.6%)	p-value	Intervention Group, n=44 (20.4%)	Muted Group, n=136 (63.0%)	Control Group, n=36 (16.7%)	p-value
Demographic Characteristics																
Age (years)	14.9 (1.1)	14.9 (1.1)	14.8 (1.1)	0.97	14.7 (1.1)	15.0 (1.1)	14.8 (1.1)	0.57	14.8 (1.1)	15.0 (1.1)	14.8 (1.2)	0.93	14.8 (1.2)	14.8 (1.1)	15.0 (1.0)	0.90
Female sex	108 (50)	50 (55.0)	58 (46.4)	0.68	29 (53.7)	49 (46.7)	30 (52.6)	0.68	38 (42.7)	49 (61.9)	31 (48.4)	0.52	29 (65.9)	63 (46.3)	16 (14.4)	0.39
Race and Ethnicity				0.72				0.57				0.48				0.54
Non-Hispanic white	166 (76.9)	75 (82.4)	91 (72.8)		43 (79.6)	77 (73.3)	46 (80.7)		69 (77.5)	48 (76.2)	49 (76.6)		32 (72.7)	109 (80.2)	25 (69.4)	
Black	10 (4.6)	4 (4.4)	6 (4.8)		2 (3.7)	8 (7.6)	0 (0.0)		3 (3.4)	3 (4.8)	4 (6.3)		2 (4.6)	6 (4.4)	2 (5.6)	
Hispanic	29 (13.4)	9 (9.9)	20 (16.0)		7 (13.0)	15 (14.3)	7 (12.3)		15 (16.9)	5 (7.9)	9 (14.1)		6 (13.6)	18 (13.2)	5 (13.9)	
Other	11 (5.1)	3 (3.3)	8 (6.4)		2 (3.7)	5 (4.8)	4 (7.0)		2.3)	7 (11.1)	2 (3.1)		6 (9.1)	3 (2.2)	4 (11.1)	
Parental Education				0.97				0.45				0.65				0.54
Graduate degree	38 (17.7)	16 (17.8)	22 (17.6)		9 (16.7)	15 (14.3)	14 (25.0)		15 (17.1)	10 (15.9)	13 (20.3)		9 (20.5)	26 (19.3)	3 (8.3)	
College Degree	91 (42.3)	37 (41.1)	54 (43.2)		29 (53.7)	43 (41.0)	19 (33.9)		37 (42.1)	27 *42.9)	27 (42.2)		20 (45.5)	55 (40.7)	16 (44.)	
Some College	62 (28.8)	25 (27.8)	37 (29.6)		13 (24.1)	30 (28.6)	19 (33.9)		29 (33.0)	13 (20.6)	20 (31.3)		8 (18.2)	39 (28.9)	15 (41.7)	

© 2019 Kahkoska AR et al. *JAMA Network Open.*

eTable 8. Baseline Characteristics of FLEX Participants by Reinforcement Learning Trees (RLT), Individualized Treatment Rule (ITR)-Assigned Subgroups for the Composite Outcome and Univariate Outcomes (HbA_{1c}, quality of life, and BMIz)

	All	A. Composite Outcome			B. Univariate Outcome: HbA1c				C. Univariate Outcome: Quality of Life				D. Univariate Outcome: BMIz			
Baseline characteristics, n (%) or mean (SD)		Intervention Group, n=91 (42.1%)	Control Group, n= 125 (57.9%)	p-value	Intervention Group, n=54 (25.0%)	Muted Group, n=105 (48.6%)	Control Group, n=57 (26.4%)	p-value	Intervention Group, n=89 (41.2%)	Muted Group, n=63 (29.2%)	Control Group, n=64 (29.6%)	p-value	Intervention Group, n=44 (20.4%)	Muted Group, n=136 (63.0%)	Control Group, n=36 (16.7%)	p-value
High School or less	24 (11.2)	12 (13.3)	12 (9.6)		3 (5.6)	17 (16.2)	4 (7.1)		7 (8.0)	13 (20.6)	4 (6.3)		7 (15.9)	15 (11.1)	2 (5.6)	
Private Health Insurance	152 (70.4)	55 (60.4)	91 (77.6)	0.26	40 (74.1)	72 (68.6)	40 (80.2)	0.77†	65 (73.0)	43 (68.3)	44 (68.8)	0.95	32 (72.7)	95 (69.9)	25 (69.4)	0.97
Single adult home	27 (12.8)	11 (12.5)	16 (13.0)	0.97	8 (15.4)	15 (14.7)	4 (7.0)	0.57	8 (9.3)	9 (14.5)	10 (15.9)	0.92	8 (18.6)	13 (9.8)	6 (17.1)	0.54
Clinical Characteristics																
Duration of diabetes (years)	6.3 (3.7)	6.7 (3.8)	6.1 (3.7)	0.68	5.8 (3.4)	6.3 (3.7)	6.8 (4.1)	0.57	6.4 (3.8)	6.3 (3.8)	6.2 (3.7)	0.95	6.1 (3.3)	6.5 (3.9)	6/1 (3.9)	0.89
HbA1c (%)	9.6 (1.2)	9.7 (1.3)	9.6 (1.2)	0.72	**9.4 (1.0)**	**9.9 (1.4)****	**9.2 (0.9)**	**0.01***	9.8 (1.2)	9.4 (1.3)	9.6 (1.2)	0.52†	9.6 (1.2)	9.6 (1.3)	9.9 (1.2)	0.68
HbA1c above 9.0%	140 (64.8)	63 (69.2)	77 (61.6)	0.676	**31 (57.4)**	**81 (77.1)****	**28 (49.1)**	**0.01***	67 (75.3)	34 (54.0)	39 (60.9)	0.52†	26 (59.1)	85 (62.5)	29 (80.6)	0.43
Insulin Regimen				0.48				0.57				0.95				0.68
Multiple daily injection	62 (28.8)	32 (35.2)	30 (24.2)		17 (31.5)	34 (32.4)	11 (18.6)		27 (30.3)	17 (27.0)	18 (28.6)		15 (34.1)	35 (25.9)	12 (33.3)	
Pump	153 (71.2)	59 (68.4)	94 (75.8)		37 (68.5)	71 (62.6)	45 (80.4)		62 (69.7)	46 (73.0)	45 (71.4)		29 (65.9)	100 (74.1)	24 (66.7)	

© 2019 Kahkoska AR et al. *JAMA Network Open.*

eTable 8. Baseline Characteristics of FLEX Participants by Reinforcement Learning Trees (RLT), Individualized Treatment Rule (ITR)-Assigned Subgroups for the Composite Outcome and Univariate Outcomes (HbA$_{1c}$, quality of life, and BMIz)

	All	A. Composite Outcome			B. Univariate Outcome: HbA1c				C. Univariate Outcome: Quality of Life				D. Univariate Outcome: BMIz			
Baseline characteristics, n (%) or mean (SD)		Intervention Group, n=91 (42.1%)	Control Group, n= 125 (57.9%)	p-value	Intervention Group, n=54 (25.0%)	Muted Group, n=105 (48.6%)	Control Group, n=57 (26.4%)	p-value	Intervention Group, n=89 (41.2%)	Muted Group, n=63 (29.2%)	Control Group, n=64 (29.6%)	p-value	Intervention Group, n=44 (20.4%)	Muted Group, n=136 (63.0%)	Control Group, n=36 (16.7%)	p-value
Used CGM in past month	43 (21.8)	20 (22.0)	42 (33.6)	0.48	15 (27.8)	33 (31.4)	14 (24.5)	0.67	29 (32.6)	17 (27.0)	16 (25.0)	0.93	15 (34.1)	40 (29.4)	7 (19.4)	0.60
BMI z-score	0.73 (0.91)	0.79 (0.82)	0.68 (0.97)	0.72	0.65 (0.89)	0.80 (0.89)	0.67 (0.98)	0.67	0.64 (0.91)	0.73 (0.94)	0.86 (0.87)	0.91	**0.97 (0.73)**	**0.49 (0.94)****	**1.3 (0.6)****	**<0.001***
Weight Status				0.72				0.57				0.93				**<0.001***
Under- or normal weight	130 (60.2)	52 (57.1)	78 (62.4)		38 (70.4)	59 (56.2)	33 (27.9)		57 (64.0)	37 (58.7)	36 (56.3)		**24 (54.6)**	**95 (69.9)****	**11 (30.6)****	
Overweight	54 (25.0)	27 (29.7)	27 (21.6)		8 (14.8)	29 (27.6)	17 (29.8)		23 (25.8)	14 (22.)	17 (26.6)		**12 (27.3)**	**30 (22.1)****	**12 (33.3)****	
Obese	32 (14.8)	12 (13.2)	20 (16.0)		8 (14.8)	17 (16.2)	7 (12.3)		9 (10.1)	12 (19.1)	11 (17.2)		**8 (18.2)**	**11 (8.1)****	**13 (26.1)****	
CGM-measures																
Glycemic Variability (coefficient of variation, %)	29.9 (7.9)	40.7 (8.6)	39.4 (7.4)	0.48	39.1 (7.3)	41.0 (8.4)	38.7 (7.5)	0.48	40.1 (8.1)	39.7 (7.3)	40.0 (8.4)	0.95	38.5 (6.0)	40.7 (8.6)	38.9 (7.2)	0.54
Average number of hypoglycemic (<70 mg/dL) episodes lasting 15 or more minutes	2 (5)	2 (5)	3 (5)	0.97	1 (5)	4 (5)	2 (6)	0.07	2 (5)	3 (5)	3.5 (6)	0.91	4 (4)	3 (6)	2 (4)	0.60

© 2019 Kahkoska AR et al. *JAMA Network Open.*

eTable 8. Baseline Characteristics of FLEX Participants by Reinforcement Learning Trees (RLT), Individualized Treatment Rule (ITR)-Assigned Subgroups for the Composite Outcome and Univariate Outcomes (HbA_{1c}, quality of life, and BMIz)

	All	A. Composite Outcome			B. Univariate Outcome: HbA1c				C. Univariate Outcome: Quality of Life				D. Univariate Outcome: BMIz			
Baseline characteristics, n (%) or mean (SD)		Intervention Group, n=91 (42.1%)	Control Group, n= 125 (57.9%)	p-value	Intervention Group, n=54 (25.0%)	Muted Group, n=105 (48.6%)	Control Group, n=57 (26.4%)	p-value	Intervention Group, n=89 (41.2%)	Muted Group, n=63 (29.2%)	Control Group, n=64 (29.6%)	p-value	Intervention Group, n=44 (20.4%)	Muted Group, n=136 (63.0%)	Control Group, n=36 (16.7%)	p-value
per 7-day period‡																
Average number of hypoglycemic (<54 mg/dL) episodes lasting 15 or more minutes per 7-day period‡	1 (2)	1 (2)	1 (2)	0.97	0 (2)	**1 (2)****	**0 (2)**	**<0.001***	1 (2)	1 (2)	1 (3)	0.93	0 (1.5)	1 (3)	0 (2)	0.39
Psychosocial and Behavioral Characteristics																
Motivation	7.6 (1.6)	7.9 (1.4)	7.5 (1.7)	0.48	7.9 (1.6)	7.6 (1.6)	7.4 (1.6)	0.57	7.5 (1.6)	7.8 (1.6)	7.7 (1.5)	0.91	7.9 (1.7)	7.5 (1.6)	7.9 (1.4)	0.43
Intention	9.0 (1.0)	9.1 (0.9)	9.0 (1.1)	0.676	9.2 (0.9)	9.1 (10.0)	8.8 (1.2)	0.48	9.0 (1.1)	9.1 (0.9)	9.1 (1.1)	0.95	9.2 (1.1)	8.9 (1.1)	9.3 (0.8)	0.54
Problem solving (SPSI)	106.0 (13.0)	105.8 (12.7)	106.1 (13.1)	0.97	104.7 (13.6)	10.7 (12.7)	105.9 (12.8)	0.68	105.1 (1.1)	107.0 (11.7)	106.2 (15.1)	0.93	106.2 (16.1)	105.1 (12.2)	108.8 (11.3)	0.60
Diabetes self-management (DSMP)	55.7 (11.4)	56.9 (10.6)	54.6 (12.0)	0.97	53.6 (11.6)	56.8 (12.1)	55.3 (10.0)	0.57	55.5 (19.7)	56.1 (11.9)	55.1 (12.2)	0.95	55.3 (13.2)	55.3 (11.1)	56.8 (11.)	0.89
Depression symptoms (CES-D)	8.9 (8.1)	8.8 (8.1)	9.1 (8.2)	0.97	10.2 (10.6)	8.8 (6.8)	8.1 (7.6)	0.57	9.8 (8.5)	6.9 (5.4)	10.0 (9.4)	0.52[†]	9.3 (11.0)	9.3 (7.5)	7.2 (6.0)	0.60

© 2019 Kahkoska AR et al. *JAMA Network Open.*

eTable 8. Baseline Characteristics of FLEX Participants by Reinforcement Learning Trees (RLT), Individualized Treatment Rule (ITR)-Assigned Subgroups for the Composite Outcome and Univariate Outcomes (HbA_{1c}, quality of life, and BMIz)

	All	A. Composite Outcome			B. Univariate Outcome: HbA1c				C. Univariate Outcome: Quality of Life				D. Univariate Outcome: BMIz			
Baseline characteristics, n (%) or mean (SD)		Intervention Group, n=91 (42.1%)	Control Group, n= 125 (57.9%)	p-value	Intervention Group, n=54 (25.0%)	Muted Group, n=105 (48.6%)	Control Group, n=57 (26.4%)	p-value	Intervention Group, n=89 (41.2%)	Muted Group, n=63 (29.2%)	Control Group, n=64 (29.6%)	p-value	Intervention Group, n=44 (20.4%)	Muted Group, n=136 (63.0%)	Control Group, n=36 (16.7%)	p-value
Generic QOL (PedsQOL)	81.3 (12.4)	81.2 (12.1)	81.2 (12.7)	0.97	79.5 (14.6)	82.4 (10.8)	80.7 (13.0)	0.57	82.3 (11.8)	81.5 (11.0)	79.3 (14.4)	0.91	81.2 (16.3)	81.7 (11.7)	79.3 (9.4)	0.77
Fear of hypoglycemia,																
Maintain High BG	1.2 (0.9)	1.2 (0.9)	1.2 (0.9)	0.97	1.1 (0.9)	1.3 (0.9)	1.1 (0.8)	0.57	1.3 (0.9)	1.2 (0.9)	1.0 (0.8)	0.78	1.1 (0.9)	1.3 (0.9)	1.0 (0.9)	0.54
Helplessness/ Worry	1.1 (0.5)	1.1 (0.6)	1.1 (0.5)	0.97	1.1 (0.6)	1.1 (0.5)	1.2 (0.7)	0.57	1.2 (0.6)	1.1 (0.6)	1.0 (0.7)	0.65	1.1 (0.7)	1.1 (0.5)	1.0 (0.6)	0.86
Worry about negative social consequences	1.1 (0.7)	1.1 0.6)	1.1 (0.8)	0.73	1.2 (0.7)	1.1 (0.7)	1.1 (0.7)	0.57	1.1 (0.8)	1.1 (0.7)	1.1 (0.7)	0.95	1.1 (0.9)	1.1 (0.7)	1.1 (0.6)	0.98
Diabetes Family Conflict	1.4 (0.3)	1.3 (0.3)	1.4 (0.4)	0.48	1.4 (0.4)	1.3 (0.3)	1.4 (0.4)	0.66	1.3 (0.3)	1.4 (0.4)	1.4 (0.4)	0.93	1.3 (0.35)	1.4 (0.3)	1.4 (0.5)	0.89

P values are from Chi squared or Fisher exact test for categorical variables, and t-tests or Kruskal-Wallis Test for continuous variables. Benjamini-Hochberg procedure was used to control for the false positive rate in multiple comparisons *denotes p<0.05. †denotes p-values that were nominally significant but lost statistical significance after adjustment for multiple comparisons. **denotes significant pairwise comparison, compared to Intervention Group (p<0.05).
‡Data were right skewed, data reported are median and IQR, p-value is from Kruskal-Wallis Test.
0 individuals were muted for the composite outcome.
Abbreviations: HbA1c – hemoglobin A1c; QOL = Quality of Life (PedsQL); BMIz – body mass index z-score; CGM – continuous glucose monitoring; SPSI – Social Problem Solving Inventory; DSMP – Diabetes Self Management Profile; CES-D – Centers for Epidemiologic Studies – Depression scale.

© 2019 Kahkoska AR et al. *JAMA Network Open.*

eTable 9. Baseline Characteristics of FLEX Participants by Outcome Weighted Learning (OWL) Individualized Treatment Rule (ITR)-Assigned Subgroups for the Composite Outcome and Univariate Outcomes (HbA_{1c}, quality of life, and BMIz)

	All (n=216)	A. Composite Outcome			B. Univariate Outcome: HbA1c			C. Univariate Outcome: Quality of Life			D. Univariate Outcome: BMIz		
**Baseline characteristics, n (%) or mean (SD)**		Intervention Group, n=101 (46.8)	Assigned to Control Group, n= 115 (53.2%)	p-value	Intervention Group, n=118 (54.6%)	Control Group, n=98 (45.4%)	p-value	Intervention Group, n=123 (56.9%)	Control Group, n=93 (43.1%)	p-value	Intervention Group, n=116 (53.7%)	Control Group, n=100 (46.3%)	p-value
**Demographic Characteristics**													
Age (years)	14.9 (1.1)	14.8 (1.2)	14.9 (1.1)	0.70	15.0 (1.1)	14.8 (1.1)	0.36	14.8 (1.1)	14.9 (1.2)	0.81	14.9 (1.1)	14.8 (1.1)	0.96
Female sex	108 (50)	58 (57.4)	50 (43.5)	0.17†	63 (54.2)	44 (44.9)	0.36	61 (49.6)	47 (50.5)	0.94	59 (50.9)	49 (49.0)	0.96
Race and Ethnicity				0.19†			**<0.001***			0.94			0.96
Non-Hispanic white	166 (76.9)	86 (85.2)	80 (69.6)		**107 (90.7)**	**59 (60.2)**		94 (76.4)	72 (77.4)		86 (74.1)	80 (80.0)	
Black	10 (4.6)	4 (4.0)	6 (5.2)		**4 (3.4)**	**6 (6.1)**		5 (4.1)	5 (5.4)		6 (5.2)	4 (4.0)	
Hispanic	29 (13.4)	8 (7.9)	21 (18.3)		**4 (3.4)**	**25 (25.5)**		18 (14.6)	11 (11.8)		19 (16.4)	10 (10.0)	
Other	11 (5.1)	**3 (3.0)**	**8 (7.0)**		**3 (2.5)**	**8 (8.2)**		6 (4.9)	5 (5.4)		5 (4.3)	6 (6.0)	
Parental Education				0.28			0.42			0.94			0.23
Graduate degree	38 (17.7)	20 (19.8)	18 (15.8)		22 (18.6)	16 (16.5)		20 (16.4)	18 (19.4)		18 (15.7)	20 (20.0)	
College Degree	91 (42.3)	39 (38.6)	52 (45.6)		49 (41.5)	42 (43.3)		53 (43.4)	38 (40.9)		43 (37.4)	48 (48.0)	
Some College	62 (28.8)	26 (25.7)	36 (31.6)		30 (25.4)	32 (33.0)		35 (28.7)	27 (29.0)		36 (31.3)	26 (26.0)	
High School or less	24 (11.2)	16 (15.8)	8 (7.0)		17 (14.4)	7 (7.2)		14 (11.5)	10 (10.8)		18 (15.7)	6 (6.0)	
Private Health Insurance	152 (70.4)	**58 (57.4)**	**94 (81.7)**	**0.001***	**71 (60.2)**	**81 (82.7)**	**0.04***	82 (66.7)	70 (75.3)	0.40	75 (64.7)	77 (77.0)	0.22
Single adult home	27 (12.8)	10 (10.10	17 *15.2)	0.44	14 (12.2)	13 (13.5)	0.78	12 (10.0)	15 (16.5)	0.40	15 (13.0)	12 (12.5)	0.96

© 2019 Kahkoska AR et al. *JAMA Network Open.*

eTable 9. Baseline Characteristics of FLEX Participants by Outcome Weighted Learning (OWL) Individualized Treatment Rule (ITR)-Assigned Subgroups for the Composite Outcome and Univariate Outcomes (HbA_{1c}, quality of life, and BMIz)

	All (n=216)	A. Composite Outcome			B. Univariate Outcome: HbA1c			C. Univariate Outcome: Quality of Life			D. Univariate Outcome: BMIz		
**Baseline characteristics, n (%) or mean (SD)**		Intervention Group, n=101 (46.8)	Assigned to Control Group, n= 115 (53.2%)	p-value	Intervention Group, n=118 (54.6%)	Control Group, n=98 (45.4%)	p-value	Intervention Group, n=123 (56.9%)	Control Group, n=93 (43.1%)	p-value	Intervention Group, n=116 (53.7%)	Control Group, n=100 (46.3%)	p-value
**Clinical Characteristics**													
Duration of diabetes (years)	6.3 (3.7)	**7.3 (3.7)**	**5.5 (3.5)**	**0.02***	5.8 (3.5)	6.8 (3.9)	0.15	6.1 (3.5)	6.7 (4.0)	0.52	**7.8 (3.8)**	**4.7 (3.0)**	**<0.001***
HbA1c (%)	9.6 (1.2)	9.8 (1.2)	9.5 (1.2)	0.24	9.6 (1.2)	9.6 (1.3)	0.78	9.5 (1.2)	9.7 (1.3)	0.94	9.6 (1.3)	9.6 (1.2)	0.96
HbA1c above 9.0%	140 (64.8)	71 (70.3)	69 (60.0)	0.24	80 (67.8)	60 (61.2)	0.42[†]	76 (61.8)	64 (68.8)	0.52	75 (64.7)	65 (65.0)	0.96
Insulin Regimen				**<0.001***			0.09			0.67			0.96
Multiple daily injection	62 (28.8)	**43 (43.0)**	**19 (16.5)**		43 (36.4)	19 (19.6)		28 (30.9)	24 (26.1)		25 (30.2)	27 (27.3)	
Pump	153 (71.2)	**57 (57.0)**	**96 (83.5)**		75 (63.6)	78 (80.4)		85 (69.1)	68 (73.9)		81 (69.8)	72 (72.7)	
Used CGM in past month	43 (21.8)	23 (22.8)	39 (33.9)	0.23	31 (26.3)	31 (31.6)	0.51	31 (25.2)	31 (33.)	0.41	34 (29.3)	28 (28.0)	0.96
BMI z-score	0.73 (0.91)	0.84 (0.81)	0.63 (0.99)	0.24	0.80 (0.78)	0.64 (1.05)	0.36	1.0 (0.90)	0.71 (0.75)	0.94	0.74 (0.90)	0.72 (0.93)	0.96
Weight Status				0.52			0.77			0.30			0.96
Under- or normal weight	130 (60.2)	56 (55.5)	74 (64.4)		70 (59.3)	60 (61.2)		66 (53.7)	64 (68.8)		69 (59.5)	61 (61.0)	
Overweight	54 (25.0)	29 (28.7)	25 (21.7)		32 (27.1)	22 (22.5)		36 (29.3)	18 (19.4)		29 (25.0)	25 (25.0)	
Obese	32 (14.8)	16 (15.8)	16 (13.9)		16 (13.6)	16 (16.3)		21 (17.1)	11 (11.8)		18 (15.5)	14 (14.0)	
CGM-measures													
Glycemic Variability (coefficient of variation, %)	29.9 (7.9)	40.5 (8.3)	39.5 (7.5)	0.52	40.6 (8.3)	39.2 (7.4)	0.36	**41.5 (8.0)**	**37.9 (7.4)**	**0.01***	39.3 (7.4)	40.7 (8.4)	0.50

© 2019 Kahkoska AR et al. *JAMA Network Open.*

eTable 9. Baseline Characteristics of FLEX Participants by Outcome Weighted Learning (OWL) Individualized Treatment Rule (ITR)-Assigned Subgroups for the Composite Outcome and Univariate Outcomes (HbA_{1c}, quality of life, and BMIz)

	All (n=216)	A. Composite Outcome			B. Univariate Outcome: HbA1c			C. Univariate Outcome: Quality of Life			D. Univariate Outcome: BMIz		
**Baseline characteristics, n (%) or mean (SD)**		Intervention Group, n=101 (46.8)	Assigned to Control Group, n= 115 (53.2%)	p-value	Intervention Group, n=118 (54.6%)	Control Group, n=98 (45.4%)	p-value	Intervention Group, n=123 (56.9%)	Control Group, n=93 (43.1%)	p-value	Intervention Group, n=116 (53.7%)	Control Group, n=100 (46.3%)	p-value
Average number of hypoglycemic (<70 mg/dL) episodes lasting 15 or more minutes per 7-day period‡	2 (5)	3 (5)	2 (4)	0.44	3 (6)	2 (5)	0.36	**4 (6)**	**1 (4)**	**<0.001***	1 (3)	4 (6)	0.09[†]
Average number of hypoglycemic (<54 mg/dL) episodes lasting 15 or more minutes per 7-day period‡	1 (2)	1 (4)	1 (2)	0.44	1 (3)	0 (2)	0.15[†]	**1 (4)**	**0 (1)**	**0.003***	1 (2)	1 (3)	0.96
**Psychosocial and Behavioral Characteristics**													
Motivation	7.6 (1.6)	7.7 (1.5)	7.5 (1.7)	0.52	7.9 (1.4)	7.4 (1.7)	0.15[†]	7.5 (1.7)	7.8 (1.5)	0.36	7.8 (1.5)	7.4 (1.7)	0.26
Intention	9.0 (1.0)	9.0 (1.0)	9.1 (1.)	0.73	9.1 (0.9)	9.0 (1.2)	0.37	9.0 (1.1)	9.2 (0.9)	0.40	9.1 (1.0)	9.0 (1.1)	0.96
Problem solving (SPSI)	106.0 (13.0)	**103.2 (13.0)**	**108.4 (12.5)**	**0.02***	104.7 (13.2)	107.5 (12.5)	0.726	**101.8 (12.7)**	**111.6 (11.0)**	**<0.001***	105.8 (13.4)	106.2 (12.5)	0.96
Diabetes self-management (DSMP)	55.7 (11.4)	56.2 (11.0)	55.0 (11.9)	0.52	56.5 (10.5)	545 (12.5)	0.36	**57.9 (11.3)**	**52.5 (10.9)**	**0.04***	57.0 (12.0)	53.9 (10.7)	0.22[†]
Depression symptoms (CES-D)	8.9 (8.1)	9.5 (8.1)	8.6 (8.1)	0.52	9.4 (8.0)	8.5 (8.3)	0.52	**10.3 (8.7)**	**7.3 (7.0)**	**0.04***	9.3 (8.9)	8.6 (7.2)	0.96
Generic QOL (PedsQOL)	81.3 (12.4)	79.8 (12.7)	82.5 (12.1)	0.24	82.2 (12.7)	82.4 (12.1)	0.36	81.3 (13.0)	81.1 (11.7)	0.94	82.3 (12.6)	79.9 (12.2)	0.39

© 2019 Kahkoska AR et al. *JAMA Network Open.*

eTable 9. Baseline Characteristics of FLEX Participants by Outcome Weighted Learning (OWL) Individualized Treatment Rule (ITR)-Assigned Subgroups for the Composite Outcome and Univariate Outcomes (HbA_{1c}, quality of life, and BMIz)

	All (n=216)	A. Composite Outcome			B. Univariate Outcome: HbA1c			C. Univariate Outcome: Quality of Life			D. Univariate Outcome: BMIz		
**Baseline characteristics, n (%) or mean (SD)**		Intervention Group, n=101 (46.8)	Assigned to Control Group, n= 115 (53.2%)	p-value	Intervention Group, n=118 (54.6%)	Control Group, n=98 (45.4%)	p-value	Intervention Group, n=123 (56.9%)	Control Group, n=93 (43.1%)	p-value	Intervention Group, n=116 (53.7%)	Control Group, n=100 (46.3%)	p-value
Fear of hypoglycemia,										0.66			0.23
Maintain High BG	1.2 (0.9)	1.3 (0.9)	1.1 (0.9)	0.52	1.2 (0.8)	1.2 (1.0)	0.36	1.2 (0.9)	1.1 (0.9)		1.1 (09)	1.3 (0.9)	
Helplessness/Worry	1.1 (0.5)	1.1 (0.5)	1.1 (0.6)	0.96	1.2 (0.6)	1.0 (0.5)	0.36	1.1 (0.6)	1.1 (0.5)	0.67	1.0 (0.5)	1.2 (0.5)	0.09†
Worry about negative social consequences	1.1 (0.7)	1.1 (0.6)	1.1 (0.8)	0.82	1.1 (0.6)	1.1 (0.8)	0.78	1.1 (0.7)	1.1 (0.7)	0.74	1.0 (0.7)	1.2 (0.7)	0.13†
Diabetes Family Conflict	1.4 (0.3)	1.3 (0.3)	1.4 (0.4)	0.10	1.3 (0.3)	1.4 (0.4)	0.15	1.4 (0.4)	1.3 (0.3)	0.69	1.4 (0.4)	1.4 (0.3)	0.96

P values are from Chi squared or fisher exact test for categorical variables, and t-tests or Kruskal-Wallis Test for continuous variables. Benjamini-Hochberg procedure was used to control for the false positive rate in multiple comparisons. *denotes $p<0.05$. †denotes p-values that were nominally significant but lost statistical significance after adjustment for multiple comparisons.
Data are mean (SD), n (%) or median (interquartile range).
1 person muted for the composite outcome and quality of life univariate outcome.
‡ Data were right skewed, data reported are median and IQR, p-value is from Kruskal-Wallis Test.
Missing data: Fear of hypoglycemia (participants) is missing 4 responses in the control group and 1 in the intervention group due to an administration error; the remaining psychosocial scales are missing up to 2 participants per intervention group due to missing responses. 115 control and 119 intervention participants provided CGM data.
CGM – continuous glucose monitoring; SPSI – Social Problem Solving Inventory; DSMP – Diabetes Self Management Profile; QOL – quality of life; CES-D – Centers for Epidemiologic Studies – Depression scale.

© 2019 Kahkoska AR et al. *JAMA Network Open.*

eFigure 1. Summary of Simple Numerical Experiment, Synergistic Setting

(A-C). RLT-predicted values of R by true treatment effect, intervention status, and true splitting variable X for the simple numerical experiment, synergistic setting. The true treatment effects are set to $\delta_1 = \delta_2 = 1,3,10$ in A, B, and C, respectively. (D-F). Differences in predicted R between intervention and control by true treatment effect, RLT ITR assignment, and true splitting variable X, as well as the true treatment effect for R, for the same numerical experiment. ITR=2 denotes the true treatment effect for R. The true treatment effects are set to $\delta_1 = \delta_2 = 1,3,10$ in D, E, and F, respectively.

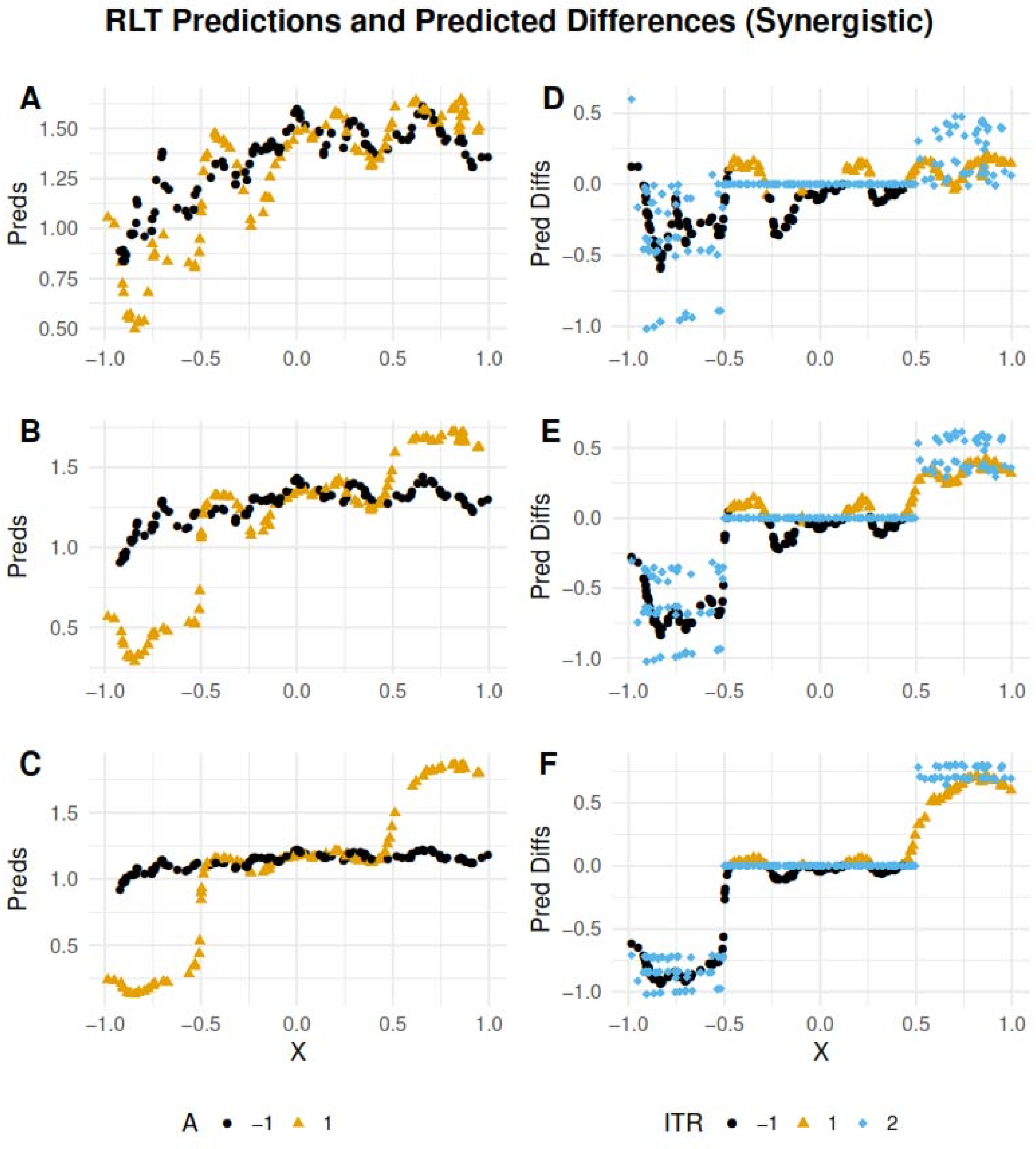

© 2019 Kahkoska AR et al. *JAMA Network Open.*

eFigure 2. Summary of Simple Numerical Experiment, Antagonistic Setting
(A-C). RLT-predicted values of R by true treatment effect, intervention status, and true splitting variable X for the simple numerical experiment, synergistic setting. The true treatment effects are set to $\delta_1 = \delta_2 = 1,3,10$ in A, B, and C, respectively. (D-F). Differences in predicted R between intervention and control by true treatment effect, RLT ITR assignment, and true splitting variable X, as well as the true treatment effect for R, for the same numerical experiment. ITR=2 denotes the true treatment effect for R. The true treatment effects are set to $\delta_1 = \delta_2 = 1,3,10$ in D, E, and F, respectively. Note the differences in shape between this panel and eFigure 1.

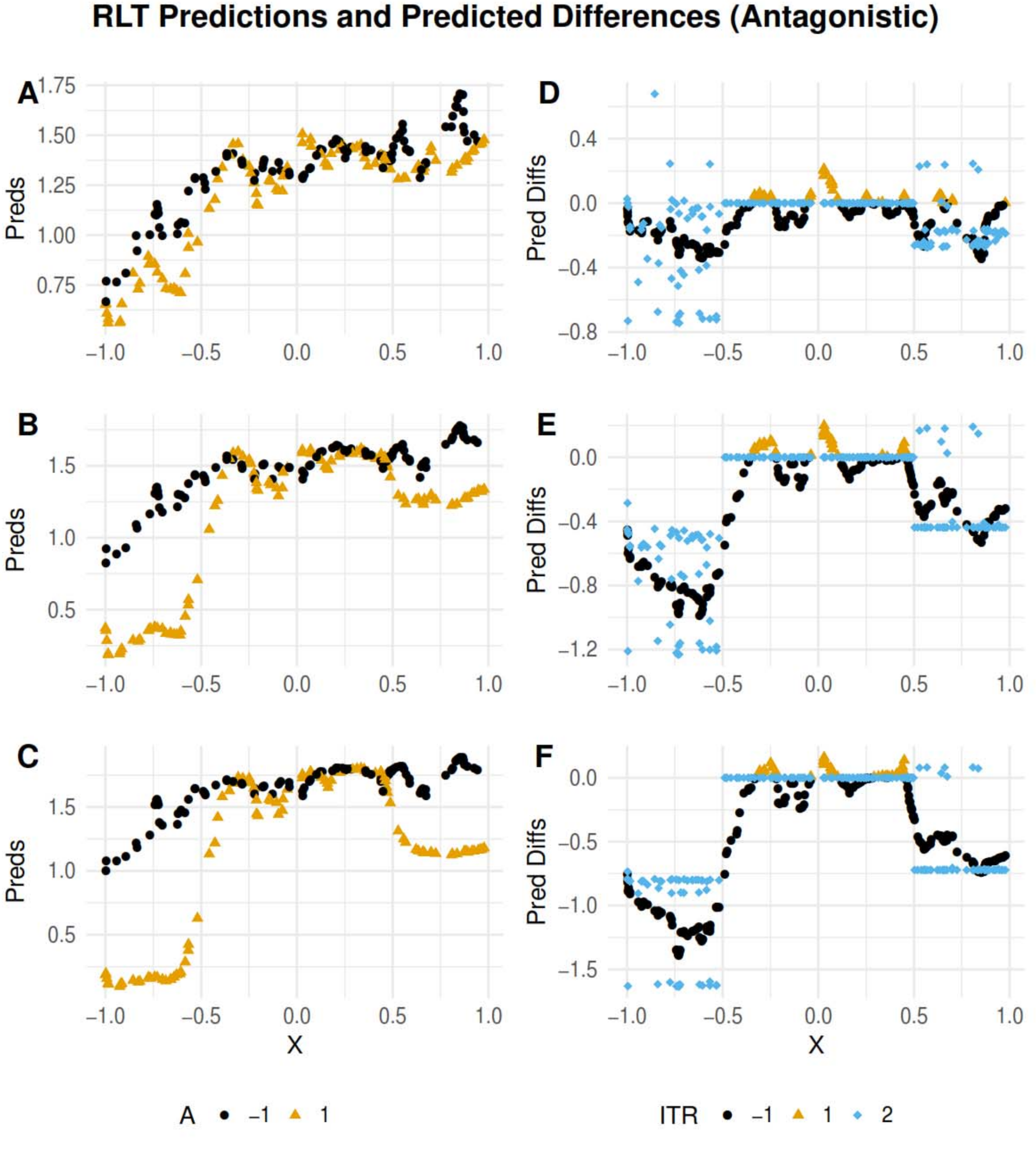

© 2019 Kahkoska AR et al. *JAMA Network Open.*

eFigure 3. Comparison to Univariate Outcome, Simple Numerical Experiment, Antagonistic Setting

(A-C). RLT-predicted values of R_1 by true treatment effect, intervention status, and true splitting variable X for the simple numerical experiment, synergistic setting. The true treatment effect is set to $\delta_1 = 1,3,10$ in A, B, and C, respectively. (D-F). Differences in predicted R_1 between intervention and control by true treatment effect, RLT ITR assignment, and true splitting variable X, as well as the true treatment effect for R_1, for the same numerical experiment. ITR=2 denotes the true treatment effect for R_1. The true treatment effects are set to $\delta_1 = 1,3,10$ in D, E, and F, respectively. Note the similarities in shape between this panel and eFigure 1.

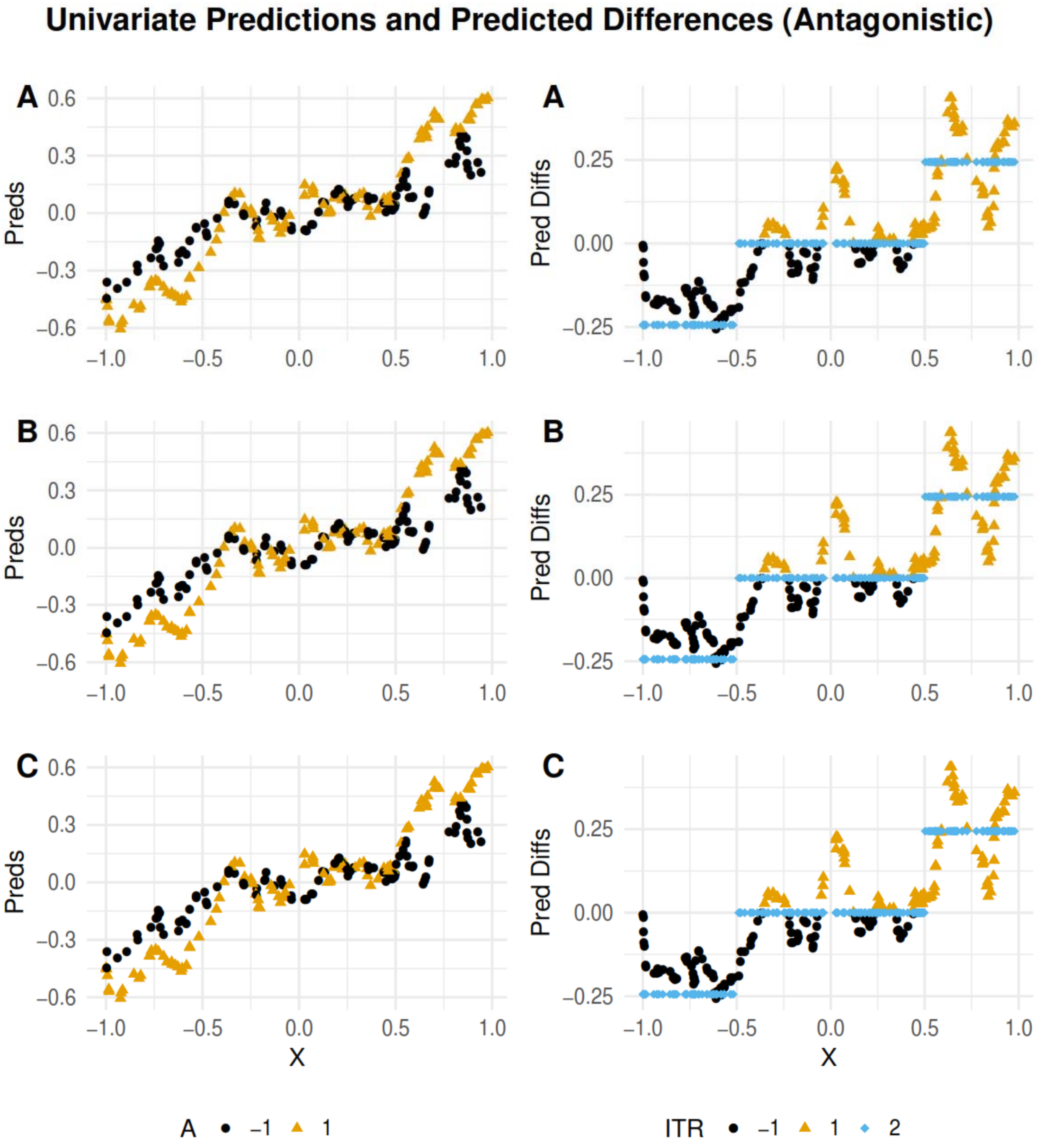

© 2019 Kahkoska AR et al. *JAMA Network Open.*

eReferences

1. Mayer-Davis EJ, Maahs DM, Seid M, et al. Efficacy of the Flexible Lifestyles Empowering Change intervention on metabolic and psychosocial outcomes in adolescents with type 1 diabetes (FLEX): a randomised controlled trial. *The Lancet Child & Adolescent Health.* 2018;2(9):635-646.
2. Danne T, Nimri R, Battelino T, Bergenstal RM, Close KL, DeVries JH, Garg S, Heinemann L, Hirsch I, Amiel SA, Beck R. International consensus on use of continuous glucose monitoring. *Diabetes care.* 2017 Dec 1;40(12):1631-40.
3. Wysocki T, Buckloh LM, Antal H, Lochrie A, Taylor A. Validation of a self-report version of the diabetes self-management profile. *Pediatric diabetes.* 2012 Aug;13(5):438-43.
4. Radloff LS. The CES-D scale: A self-report depression scale for research in the general population. *Applied psychological measurement.* 1977 Jun;1(3):385-401.
5. Varni JW, Seid M, Kurtin PS. PedsQL™ 4.0: Reliability and validity of the Pediatric Quality of Life Inventory™ Version 4.0 Generic Core Scales in healthy and patient populations. *Med Care.* 2001 Aug 1;39(8):800-12.
6. Shepard JA, Vajda K, Nyer M, Clarke W, Gonder-Frederick L. Understanding the construct of fear of hypoglycemia in pediatric type 1 diabetes. *Journal of pediatric psychology.* 2014 Sep 11;39(10):1115-25.
7. Nathan DM, DCCT/Edic Research Group. The diabetes control and complications trial/epidemiology of diabetes interventions and complications study at 30 years: overview. *Diabetes Care.* 2014 Jan 1;37(1):9-16.
8. DCCT Research Group. Weight gain associated with intensive therapy in the diabetes control and complications trial. *Diabetes Care.* 1988 Jul 1;11(7):567-73.
9. Carlson MG, Campbell PJ. Intensive insulin therapy and weight gain in IDDM. *Diabetes.* 1993 Dec 1;42(12):1700-7.
10. American Diabetes Association. 11. Children and adolescents. *Diabetes Care.* 2016 Jan 1;39(Supplement 1):S86-93.
11. Zhao Y, Zeng D, Rush AJ, Kosorok MR. Estimating individualized treatment rules using outcome weighted learning. *Journal of the American Statistical Association.* 2012;107(499):1106-1118.
12. Zhou X, Mayer-Hamblett N, Khan U, Kosorok MR. Residual weighted learning for estimating individualized treatment rules. *Journal of the American Statistical Association.* 2017 Jan 2;112(517):169-87.
13. Fu H, Zhou J, Faries DE. Estimating optimal treatment regimes via subgroup identification in randomized control trials and observational studies. *Statistics in medicine.* 2016 Aug 30;35(19):3285-302.
14. Chen J, Fu H, He X, Kosorok MR, Liu Y. Estimating individualized treatment rules for ordinal treatments. *Biometrics.* 2018 Sep;74(3):924-33.
15. Chen G, Zeng D, Kosorok MR. Personalized dose finding using outcome weighted learning. *Journal of the American Statistical Association.* 2016 Oct 1;111(516):1509-21.
16. Baum A, Scarpa J, Bruzelius E, Tamler R, Basu S, Faghmous J. Targeting weight loss interventions to reduce cardiovascular complications of type 2 diabetes: a machine learning-based post-hoc analysis of heterogeneous treatment effects in the Look AHEAD trial. *The Lancet Diabetes & Endocrinology.* 2017;5(10):808-815.
17. Chakraborty B, Murphy SA. Dynamic treatment regimes. *Annual review of statistics and its application.* 2014;1:447-464.
18. VanderWeele TJ, Knol MJ. Interpretation of subgroup analyses in randomized trials: heterogeneity versus secondary interventions. *Annals of internal medicine.* 2011;154(10):680-683.
19. Gueorguieva R, Mallinckrodt C, Krystal JH. Trajectories of depression severity in clinical trials of duloxetine: insights into antidepressant and placebo responses. *Archives of general psychiatry.* 2011;68(12):1227-1237.
20. Kahkoska AR CJ DK, Hunter C, Seid M, Mayer-Davis EJ, Maahs DM. Effect Modifiers of a Behavioral Intervention in Adolescents with Type 1 Diabetes: The FLEX study. Oral Presentations. *Pediatric Diabetes.* 2018;19(S26):14-40.
21. Burton H, Sagoo GS, Pharoah P, Zimmern RL. Time to revisit Geoffrey Rose: strategies for prevention in the genomic era? *Italian Journal of Public Health.* 2012;9(4).
22. Trusheim MR, Berndt ER, Douglas FL. Stratified medicine: strategic and economic implications of combining drugs and clinical biomarkers. *Nature reviews Drug discovery.* 2007;6(4):287-293.

© 2019 Kahkoska AR et al. *JAMA Network Open.*

23. Kichler JC, Seid M, Crandell J, et al. The Flexible Lifestyle Empowering Change (FLEX) intervention for self-management in adolescents with type 1 diabetes: Trial design and baseline characteristics. *Contemporary Clinical Trials.* 2017.
24. White IR, Royston P, Wood AM. Multiple imputation using chained equations: issues and guidance for practice. *Statistics in medicine.* 2011;30(4):377-399.
25. Stekhoven DJ, Bühlmann P. MissForest—non-parametric missing value imputation for mixed-type data. *Bioinformatics.* 2011;28(1):112-118.
26. Zhu R, Zeng D, Kosorok MR. Reinforcement Learning Trees. *J Am Stat Assoc.* 2015;110(512):1770-1784.
27. Breiman L. Random forests. *Machine learning.* 2001;45(1):5-32.
28. Thissen D, Steinberg L, Kuang D. Quick and easy implementation of the Benjamini-Hochberg procedure for controlling the false positive rate in multiple comparisons. *Journal of Educational and Behavioral Statistics.* 2002;27(1):77-83.
29. Hampson SE, Skinner TC, Hart J, et al. Behavioral interventions for adolescents with type 1 diabetes: how effective are they? *Diabetes Care.* 2000;23(9):1416-1422.
30. Channon SJ, Huws-Thomas MV, Rollnick S, et al. A multicenter randomized controlled trial of motivational interviewing in teenagers with diabetes. *Diabetes Care.* 2007;30(6):1390-1395.
31. Wang YC, Stewart SM, Mackenzie M, Nakonezny PA, Edwards D, White PC. A randomized controlled trial comparing motivational interviewing in education to structured diabetes education in teens with type 1 diabetes. *Diabetes Care.* 2010;33(8):1741-1743.
32. Cole TJ, Faith MS, Pietrobelli A, Heo M. What is the best measure of adiposity change in growing children: BMI, BMI%, BMI z-score or BMI centile? *European journal of clinical nutrition.* 2005;59(3):419.
33. Khoury MJ, Gwinn ML, Glasgow RE, Kramer BS. A population approach to precision medicine. *American journal of preventive medicine.* 2012;42(6):639-645.
34. Ioannidis JP. Why most published research findings are false. *PLoS medicine.* 2005;2(8):e124.
35. Simmons JP, Nelson LD, Simonsohn U. False-positive psychology: Undisclosed flexibility in data collection and analysis allows presenting anything as significant. *Psychological science.* 2011;22(11):1359-1366.
36. Kosorok MR. Precision Medicine. *Annual Reviews of Statistics and its Application.* Vol In Press. 2018.

© 2019 Kahkoska AR et al. *JAMA Network Open.*
